# SARM1 loss protects retinal ganglion cells in a mouse model of autosomal dominant optic atrophy

**DOI:** 10.1172/JCI191315

**Published:** 2025-05-09

**Authors:** Chen Ding, Papa S. Ndiaye, Sydney R. Campbell, Michelle Y. Fry, Jincheng Gong, Sophia R. Wienbar, Whitney Gibbs, Philippe Morquette, Luke H. Chao, Michael Tri H. Do, Thomas L. Schwarz

**Affiliations:** 1 F.M. Kirby Neurobiology Center, Boston Children’s Hospital, Boston, Massachusetts, USA.; 2 Department of Neurobiology, and; 3 Department of Neurology, Harvard Medical School, Boston, Massachusetts, USA.; 4 Department of Molecular Biology, Massachusetts General Hospital, Boston, Massachusetts, USA.; 5 Department of Genetics, Blavatnik Institute, Harvard Medical School, Boston, Massachusetts, USA.

**Keywords:** Cell biology, Neuroscience, Therapeutics, Mitochondria, Neurodegeneration

## Abstract

Autosomal dominant optic atrophy (ADOA), the most prevalent hereditary optic neuropathy, leads to retinal ganglion cell (RGC) degeneration and vision loss. ADOA is primarily caused by mutations in the optic atrophy type 1 (*OPA1*) gene, which encodes a conserved GTPase important for mitochondrial inner membrane dynamics. To date, the disease mechanism remains unclear, and no therapies are available. We generated a mouse model carrying the pathogenic *Opa1^R290Q/+^* allele that recapitulated key features of human ADOA, including mitochondrial defects, age-related RGC loss, optic nerve degeneration, and reduced RGC functions. We identified sterile alpha and TIR motif containing 1 (SARM1), a neurodegeneration switch, as a key driver of RGC degeneration in these mice. *Sarm1* KO nearly completely suppressed all the degeneration phenotypes without reversing mitochondrial fragmentation. Additionally, we show that a portion of SARM1 localized within the mitochondrial intermembrane space. These findings indicated that SARM1 was activated downstream of mitochondrial dysfunction in ADOA, highlighting it as a promising therapeutic target.

## Abstract 2

The researchers built a novel mouse model of Autosomal Dominant Optic Atrophy and showed that knocking out SARM1 strongly suppresses Retinal Ganglion Cell degeneration.

## Introduction

Autosomal dominant optic atrophy (ADOA) is one of the most common hereditary optic neuropathies, affecting approximately 1 in 30,000 individuals worldwide (1, 2). ADOA primarily causes degeneration of retinal ganglion cells (RGCs) and their axons, which form the optic nerve (3). Vision loss in patients with ADOA typically begins during the first decade of life, with symptoms that usually progress slowly and can lead to legal blindness in severe cases (4). At least 70% of ADOA cases are caused by mutations in optic atrophy type 1 (*OPA1*) (2, 5–8), a nuclear gene encoding a conserved GTPase essential for inner mitochondrial membrane fusion and cristae maintenance (9, 10). Homozygous loss of *OPA1* is embryonically lethal, and most patients with ADOA are heterozygous carriers exhibiting haploinsufficiency (11). However, certain missense mutations in the GTPase domain can act in a dominant-negative (DN) fashion (12, 13) and are often associated with a higher risk of developing a more severe syndromic disorder known as ADOA-plus and characterized by additional symptoms such as deafness, ataxia, myopathy, peripheral neuropathy, and progressive external ophthalmoplegia (12, 13). Previous ADOA mouse models, including those with a Q285stop mutation (14), a c.1065+5G splice site mutation (15), and a c.2708_2711delTTAG mutation (16), reflect OPA1 haploinsufficiency and exhibit mitochondrial fragmentation and cristae abnormalities. Consequently, these mice show varying degrees of age-dependent RGC loss, optic nerve degeneration, dendritic loss, demyelination, and reduced visual responses (14–17). In contrast, mouse models addressing missense mutations, which comprise over a quarter of pathogenic human *OPA1* variants (18), have not been established. Knocking out both copies of OPA1 selectively in RGCs, though unlikely to reflect real patient conditions, appears to cause even more severe visual defects at a younger age (19). Despite our understanding of the genetic underpinnings, there are currently no treatments available, and our comprehension of the pathological mechanisms of ADOA remains limited.

*OPA1* is tightly regulated at both the RNA and protein levels. Alternative splicing of exons 4, 4b, and 5b produces 8 *OPA1* isoforms in humans and 4 in mice. The full-length OPA1 protein, which contains an N-terminal transmembrane domain, is embedded in the inner mitochondrial membrane. It undergoes proteolytic processing, with cleavage at the S1 site by the OMA1 protease (20, 21) and at the S2 and S3 sites by the YME1L protease (22–24), producing shorter forms that are released into the mitochondrial intermembrane space (IMS). Regulation of these protease activities (25, 26) maintains a balance between long and short OPA1 forms that cooperate to mediate membrane fusion and preserve cristae structure (27–30). Disruption of OPA1 function can cause severe mitochondrial damage, including reduced respiratory efficiency, increased oxidative stress, and loss of mitochondrial DNA (mtDNA) (31, 32).

*SARM1*, a major prodegenerative factor in neurons, encodes a conserved NADase that cleaves nicotinamide adenine dinucleotide (NAD^+^) into nicotinamide and cyclic ADP-ribose (cADPR) (33). Sterile alpha and TIR motif containing 1 (SARM1) has a mitochondrial targeting sequence, an N-terminal ARM domain followed by 2 SAM domains, and a C-terminal TIR domain. The TIR domain possesses intrinsic NAD^+^ cleavage activity but in healthy neurons is autoinhibited by the ARM domains (34). Under certain degenerative conditions, the autoinhibition is released by an elevated NMN/NAD^+^ ratio and triggers a positive feedback loop that rapidly consumes cytosolic NAD^+^ (35). NAD^+^ depletion initiates a cascade of cellular events including ATP loss, calcium influx, and calpain activation that culminates in axon degeneration and neuronal cell death (36). *Sarm1* KO can prevent degeneration in several neurodegenerative conditions, including glaucoma, traumatic brain injury, and peripheral neuropathy (37–40). Of particular interest is the role of SARM1 in degeneration induced by mitochondrial dysfunction, such as in Charcot-Marie-Tooth disease type 2A (CMT2A), which is caused by *mitofusin* mutations that disrupt mitochondrial outer membrane fusion (41–43). Because SARM1 inhibition has little or no effect in normal tissues and appears to be activated only to trigger neurodegeneration, it has been identified as a promising therapeutic target (44). The connection between SARM1, mitochondrial dysfunction, and retinal degeneration prompted us to investigate whether SARM1 drives RGC degeneration in ADOA.

In this study, we developed and characterized a mouse model of ADOA by introducing the pathogenic *Opa1^R290Q/+^* missense mutation, which has been reported in several ADOA families (11, 45, 46). We then studied the effect of *Sarm1* deletion on the progression of optic atrophy in this mouse model.

## Results

### Generation of the Opa1^R290Q/+^ mouse and allele characterization.

**Figure 1:**
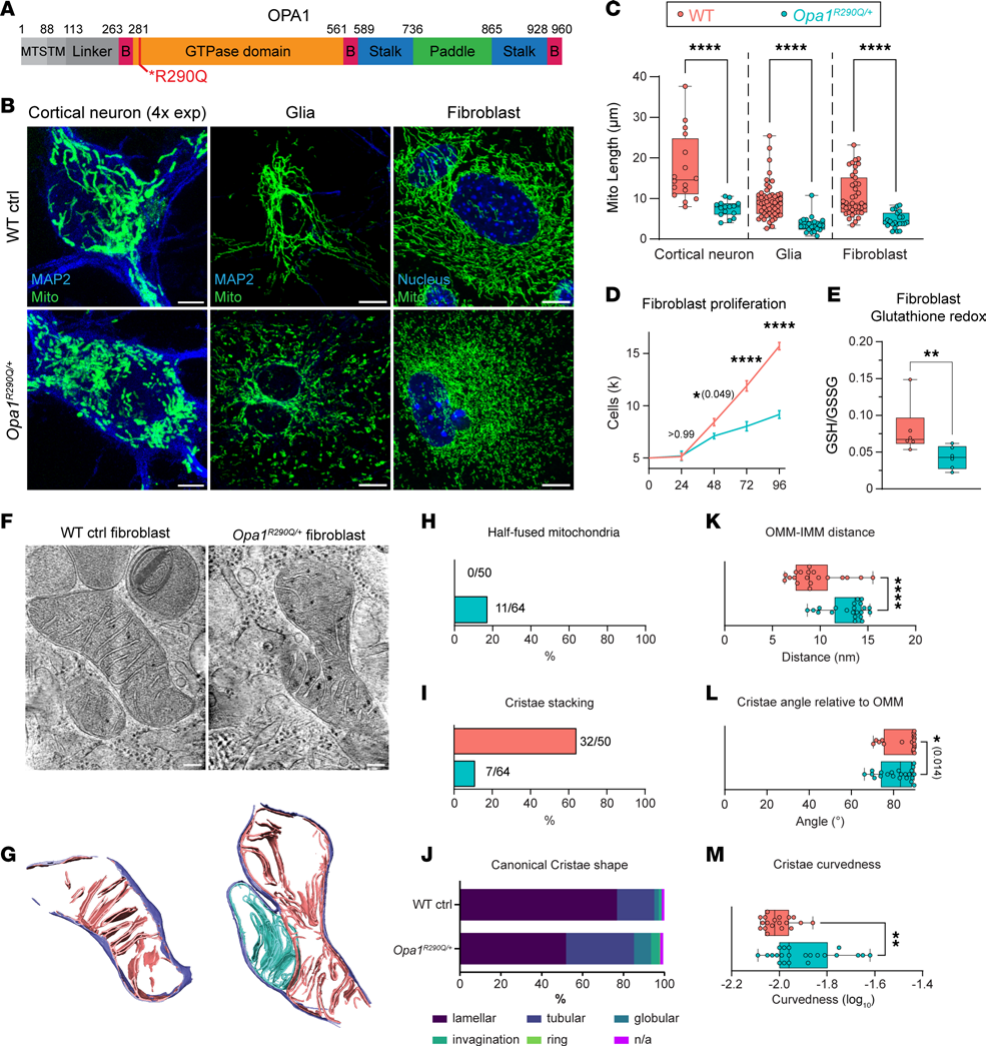
The *Opa1^R290Q/+^* mutation impairs mitochondrial morphology and cristae structure. (**A**) Mouse OPA1 domains (90). MTS, mitochondrial targeting sequence; TM, transmembrane domain; B, bundle signaling element. Schematic was created with BioRender. (**B**) Representative images of mitochondria in cortical neurons, glial cells, and fibroblasts isolated from *Opa1*^R290Q/+^ mice and WT littermate controls. Scale bars: 10 μm. (**C**) Quantification of mitochondrial length in cortical neurons (*n* = 16 WT neurons and 15 *Opa1^R290Q/+^* neurons from 2 cultures), glia (*n* = 51 WT glia and 23 *Opa1^R290Q/+^* glia from 1 culture), and fibroblasts (*n* = 39 WT cells from 2 experiments and 23 *Opa1^R290Q/+^* cells from 3 experiments). (**D**) *Opa1^R290Q/+^* fibroblasts grew at a slower rate than did the WT control (*n* = 6 wells per genotype). Data indicate the mean ± SEM. (**E**) Fibroblasts GSH/GSSG ratios measured by LC-MS/MS. *n* = 6 samples per genotype from 2 experiments. redox, reduction-oxidation. (**F** and **G**) Representative summed projections of the central slices of cryo-electron tomograms of mitochondria (**F**) and corresponding 3D segmentations of the entire stack (**G**). In the *Opa1^R290Q/+^* cell, the OMM (purple in **G**) has fused, while the IMMs (blue and pink in **G**) remains separate. Scale bars: 100 nm. (**H**) Percentage of mitochondria with fused OMM but unfused IMM. The numbers of stalled-fusion/total mitochondria are indicated. (**I**) Percentage of WT mitochondria (*n* = 50 WT and 64 *Opa1^R290Q/+^*) displaying stacked cristae. (**J**) Classification of cristae morphology (*n* = 362 WT and *n* = 337 *Opa1^R290Q/+^* cristae). (**K**–**M**) The highest-frequency values for OMM-IMM distance (**K**), cristae angle relative to the OMM (**L**), and cristae curvedness (**M**) (*n* = 19 WT and 22 *Opa1^R290Q/+^* mitochondria). Box plots denote minimum, first quartile, median, third quartile, and maximum values. *P < 0.05, **P < 0.01, and ****P < 0.0001 by Mann-Whitney *U* test (**C**, **E**, and **K**–**M**) and 2-way ANOVA with Šidák’s multiple-comparison test (**D**). ctrl, control; mito, mitochondria.

We used the CRISPR/Cas9 technique to introduce R290Q, a pathogenic mutation identified in patients (11, 45, 46), into the *Opa1* locus of C57BL/6J mice (Supplemental Figure 1A; supplemental material available online with this article; https://doi.org/10.1172/JCI191315DS1). Homozygous mice are embryonically lethal, but heterozygotes are viable and fertile, as has been seen with other OPA1 mutations (14–16). The R290Q mutation is located in the first β-strand of the dynamin-type GTPase domain and included in all *Opa1* isoforms and cleavage products (Figure 1A and Supplemental Figure 1B). This mutation is distal from the nucleotide binding site and potentially alters the folding of the GTPase domain core while leaving the C-terminal stalk and paddle regions intact (47). Unlike heterozygous alleles with early-stop codons that reduce OPA1 protein levels by half (14–16), the R290Q mutation appeared to alter OPA1 protein processing: lower-molecular-weight isoforms were reduced and 2 higher-molecular-weight bands appeared (Supplemental Figure 1, B and C). Similar changes were observed in human *OPA1^R290Q/+^* fibroblasts (46). The new bands likely correspond to full-length isoforms 5 and 8, which are completely cleaved into short forms and thus absent in WT controls (22, 24). PCR analysis of *Opa1* isoforms in reverse-transcribed cDNAs from cultured *Opa1^R290Q/+^* fibroblasts and primary cortical neurons showed no detectable changes in *Opa1* transcript abundance (Supplemental Figure 1D).

### The Opa1^R290Q/+^ mutation causes mitochondrial fragmentation, impairs mitochondrial function, and exhibits a DN effect.

We first examined the effect of the *Opa1^R290Q/+^* mutation on mitochondrial morphology in primary cells isolated from *Opa1^R290Q/+^* mice and their WT littermate controls. In cultured 9 days-in-vitro (DIV9) cortical neurons, mitochondria were labeled via lentiviral transduction of MitoDsRed, and the network in the somata was resolved using a hydrogel-based expansion technique (48) (Figure 1B). *Opa1^R290Q/+^* neurons exhibited mitochondrial fragmentation, with the average mitochondrial length reduced by 57% compared with WT (Figure 1, B and C). We observed a similar reduction (62%) in microtubule-associated protein 2 (MAP2^–^) glial cells (Figure 1, B and C). In cultures of *Opa1^R290Q/+^* embryonic fibroblasts stained for endogenous ATP5α, we observed that mitochondria were also fragmented, with average length reduced to 43% of WT lengths (Figure 1, B and C). These phenotypes are consistent with severe impairment of the fusion activity of OPA1 by the R290Q mutation, as previously reported in human fibroblasts (46), and with the significance of OPA1 function for all cells.

The *OPA1^R290Q/+^* allele has DN properties that can cause the ADOA-plus phenotype in some patients (45, 46). To test whether the R290Q mutation acts dominantly in mice, we induced overexpression of WT OPA1 or the R290Q OPA1 isoform 1 as a GFP fusion protein in WT fibroblasts (Supplemental Figure 2A). While WT OPA1 overexpression did not significantly alter mitochondrial length, the OPA1 R290Q transgene reduced it by 40% (Supplemental Figure 2B), indicating a DN effect stronger than a simple loss-of-function mutation.

In addition to morphological changes, the R290Q mutation also induced cellular changes consistent with mitochondrial dysfunction. *Opa1^R290Q/+^* fibroblasts proliferated at a slower rate than did WT control fibroblasts (Figure 1D) and had a significantly lower ratio of reduced glutathione to oxidized glutathione disulfide (GSH/GSSG), as measured by liquid chromatography tandem mass spectrometry (LC-MS/MS) (49), indicating cellular oxidative stress (Figure 1E) (50). We observed the same change in glutathione redox in whole brain metabolites (Supplemental Figure 3A). In the Agilent Seahorse assay of mitochondrial respiration, mutant fibroblasts showed slightly reduced baseline oxygen consumption rates and proton leakage, with minimal changes in maximum respiration (Supplemental Figure 3, C and D). Additionally, mtDNA copy numbers remained unchanged, as measured by reverse transcription quantitative PCR (RT-qPCR) using 3 mitochondrial genes (Supplemental Figure 3B), consistent with findings from 2 previous *Opa1* haploinsufficiency mouse models (15, 16). In summary, the *Opa1^R290Q/+^* mutation induced mitochondrial fragmentation and oxidative stress but had little effect on respiration and mtDNA copy numbers.

### Mitochondria in Opa1^R290Q/+^ fibroblasts have aberrant cristae.

In addition to mediating membrane fusion, OPA1 plays an important role in shaping mitochondrial cristae (10). We performed cryo–electron tomography (cryo-ET) to examine 3D cristae ultrastructure on cryo-focused ion beam–milled (cryo-FIB–milled) fibroblasts. We focused on the fibroblasts, given their homogeneity, abundant mitochondria, and ability to be grown on the appropriate support grids. WT and *Opa1^R290Q/+^* fibroblasts were deposited on grids, back-blotted, and vitrified in liquid ethane. Windows in the cells were milled with a cryo-focused ion beam scanning electron microscope (cryo-FIB-SEM) to generate 150–200 nm thick lamellae, where mitochondria were targeted and tilt series were collected in a 300 keV Titan Krios and processed to generate 3D tomograms (Figure 1, F and G).

When examining gross mitochondrial morphology, we observed a notable fusion defect: 11 of 64 *Opa1^R290Q/+^* mitochondria exhibited a fused outer mitochondrial membrane (OMM) but separated inner mitochondrial membrane (IMM). This half-fused state was not observed in WT mitochondria (Figure 1, G and H), and this defect, specifically lacking inner membrane fusion, is consistent with impaired OPA1 function. We interpret these mitochondria as having stalled IMM fusion, rather than being in the process of fission, based on 2 observations: (a) the absence of endoplasmic reticulum (ER), actin, or septin filaments proximal to the contact site of the 2 mitochondria (51, 52); and (b) the lack of any potential dynamin-related protein 1 (DRP1) densities corresponding to a constriction ring at the OMM (53, 54). Furthermore, IMM severing likely occurs either simultaneously or shortly after OMM severing during fission, making visualization of intermediates unlikely. We propose that the observation of 2 distinct IMMs with a fused OMM in the *Opa1^R290Q/+^* mitochondria represents an intermediate state in the fusion process, since fusion of the outer and inner membranes occurs sequentially and has been previously shown to be able to be decoupled in cells and in vitro (55, 56).

We also noted differences in cristae architecture in the *Opa1^R290Q/+^* mitochondria. A reduction in 3 or more parallel stacked cristae was observed in *Opa1^R290Q/+^* mitochondria (Figure 1I). Cristae were classified into the following canonical shape categories: lamellar (sheet-like), tubular, globular (balloon-like), ring, invagination (short projections into the mitochondria matrix), or undetermined (Figure 1J). We observed a reduction in lamellar cristae and increase in tubular cristae in *Opa1^R290Q/+^* mitochondria, consistent with the observed decrease in stacked cristae.

Cristae architectural differences were further quantified by applying a morphometrics toolkit to analyze segmented membranes from mitochondria in WT (*n* = 19 mitochondria) and *Opa1^R290Q/+^* (*n* = 22 mitochondria) cells (57). One notable change was that the distance between the OMM and IMM was larger in *Opa1^R290Q/+^* mitochondria compared with WT (Figure 1K). This suggests that, in addition to impairing fusion, the mutation disrupted the machinery that maintains the spacing between inner and outer membranes. In mutant mitochondria, cristae were less perpendicular relative to the OMM and more curved compared with WT mitochondria (Figure 1, L and M). Thus, these Cryo-ET data reveal that the *Opa1^R290Q/+^* mutation impaired cristae ultrastructure in addition to its adverse effects on mitochondrial networks and morphology.

### Age-related RGC death and optic nerve degeneration in Opa1^R290Q/+^ mice.

**Figure 2:**
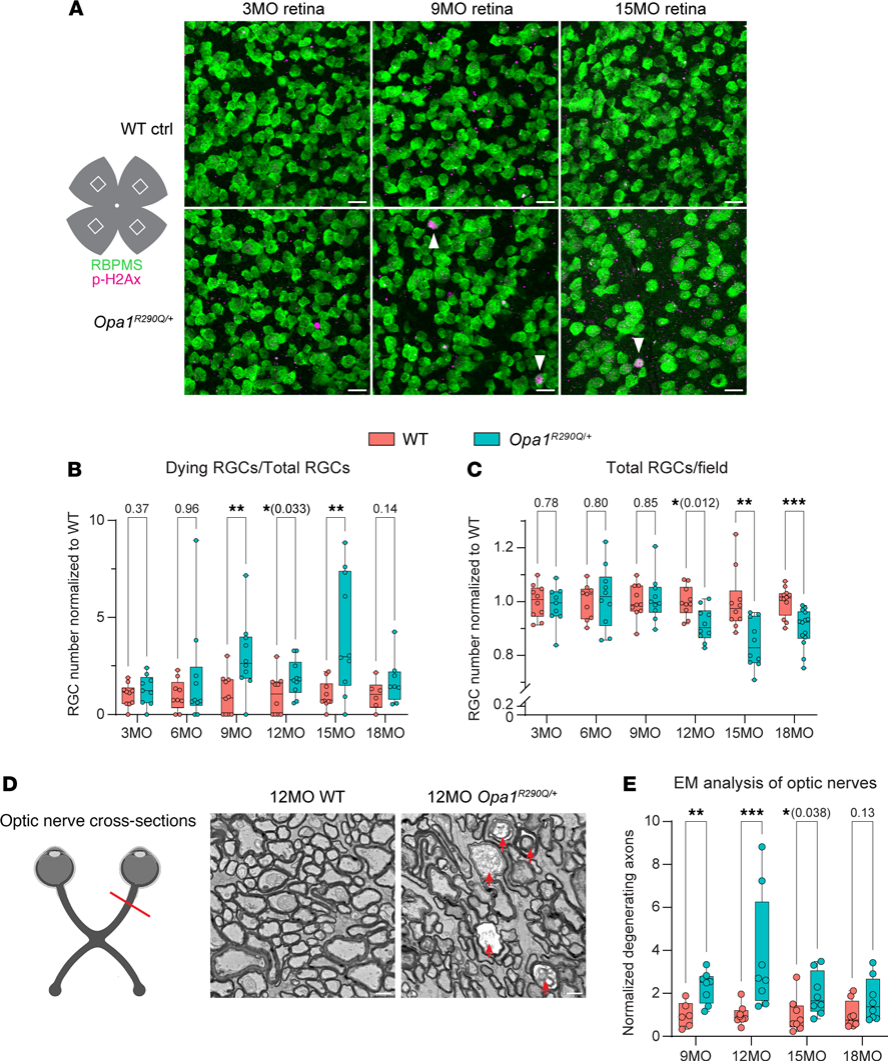
Age-related RGC and optic nerve degeneration in *Opa1^R290Q/+^* mice. (**A**) Representative images of retinal whole mounts from WT and *Opa1^R290Q/+^* mice. Each retina was imaged in every quadrant, approximately 1.2 mm from the optic nerve head. RBPMS (green) marks all RGCs and p-H2Ax (magenta) labels RGCs undergoing cell death (marked by arrowheads). Scale bars: 20 μm. (**B**) Quantification of dying RGCs over time. Each dot represents 1 retina. The counts of dying RGCs were averaged across the 4 quadrants of each retina and divided by the average RGC number of those quadrants. This value was then normalized to the WT average at each age. *n* = 9–12 WT and *n* = 9–14 *Opa1^R290Q/+^* retinas per age group. (**C**) Quantification of total RGCs over time. Each dot represents 1 retina. RGC counts were first averaged across the 4 quadrants of each retina and then normalized to the WT average at each age. *n* = 6–10 WT and *n* = 8–10 *Opa1^R290Q/+^* retinas per age group. (**D**) Diagram of optic nerve cross-sections and representative EM images from 12MO animals. Arrows indicate degenerating axons. Scale bars: 1 μm. Diagram was created with BioRender. (**E**) Quantification of degenerating axons. Each dot represents 1 optic nerve. The counts of degenerating axons were averaged across the 9 fields of each cross-section of an optic nerve and divided by the average axon number of those fields. This value was then normalized to the WT average at each age. *n* = 6–8 WT and *n* = 8 *Opa1^R290Q/+^* retinas per age group. Box plots denote minimum, first quartile, median, third quartile, and maximum values. *P < 0.05, **P < 0.01, and ***P < 0.001, by Mann-Whitney *U* test for each age group.

RGCs are the primary cells affected by ADOA. To study RGC death and survival, we examined retinal whole mounts from a large cohort of *Opa1^R290Q/+^* mice and their littermate WT controls, with equal representation of the sexes, and ranging from 3–18 months of age at 3-month intervals. Retinas were stained for RNA-binding protein with multiple splicing (RBPMS), a pan-RGC marker (58), and phosphorylated H2Ax (p-H2Ax), a histone marker for DNA double-stranded breaks, which labels cells undergoing cell death (59) (Figure 2A, arrowheads highlight p-H2Ax^+^ RGCs). Quantifying p-H2Ax^+^ RGCs provides a robust readout of ongoing degeneration, as RGC death is very rare in control retinas. In 3- and 6-month-old (MO) animals, WT and *Opa1^R290Q/+^* retinas had similar numbers of RGCs and percentages of dying RGCs (Figure 2, A–C). However, by 9 months of age, *Opa1^R290Q/+^* mice displayed a 3-fold increase in dying RGCs compared with age-matched WT controls, although this was not yet reflected in a significant decrease in the total RGC count (Figure 2, A–C). At 12 months, a reduction in total RGCs was apparent in *Opa1^R290Q/+^* mice alongside an increase in dying RGCs. By 15 months, the mean total RGC number had decreased by 16% compared with WT, with dying RGCs rising to 4-fold of the WT level (Figure 2, A–C). At 18 months, the difference in dying RGCs was largely diminished, and there was no further decrease in the total RGC number (Figure 2, B and C, also see Supplemental Figure 4, A and B, for un-normalized quantifications). Taken together, these findings indicate that retinas developed normally in *Opa1^R290Q/+^* mice and that subsequent RGC death predominantly occurred in mice between 9 and 15 months of age. The speed and degree of RGC degeneration were consistent with observations in patients with ADOA, in whom vision decline is typically slow, heterogeneous, and does not involve massive RGC loss across the entire retina, but rather predominantly affects subsets of RGCs, such as those with small fibers in the papillomacular bundle (2, 60, 61).

Electron microscopy (EM) images of optic nerve cross-sections indicated progressive degeneration of RGC axons. Healthy axons are characterized by well-aligned microtubules and a compact myelin sheath (Figure 2D and Supplemental Figure 5A). In *Opa1^R290Q/+^* mutants, we observed that a fraction of the axons were undergoing recognizable stages of degeneration: early-stage degeneration included axon swelling and accumulation of organelles or neurofilaments, followed by axoplasm darkening in the late stage, and eventually leading to fully degenerated axons with only empty myelin sheaths remaining (Figure 2D and Supplemental Figure 5A). Between 9 and 15 months, the fraction of degenerating axons was significantly higher in mutants compared with WT (Figure 2E and Supplemental Figure 4C). By 18 months, the difference was no longer significant, consistent with the observed pattern of RGC cell death (Figure 2B).

We also assessed mitochondrial density in RGC axons by EM in 12MO optic nerves and observed no appreciable changes (Supplemental Figure 5, B and C), suggesting that mitochondria transport from the soma to the axon was not impaired by the *Opa1^R290Q/+^* mutation.

### Visual evoked potentials decline with age in Opa1^R290Q/+^ mice.

**Figure 3:**
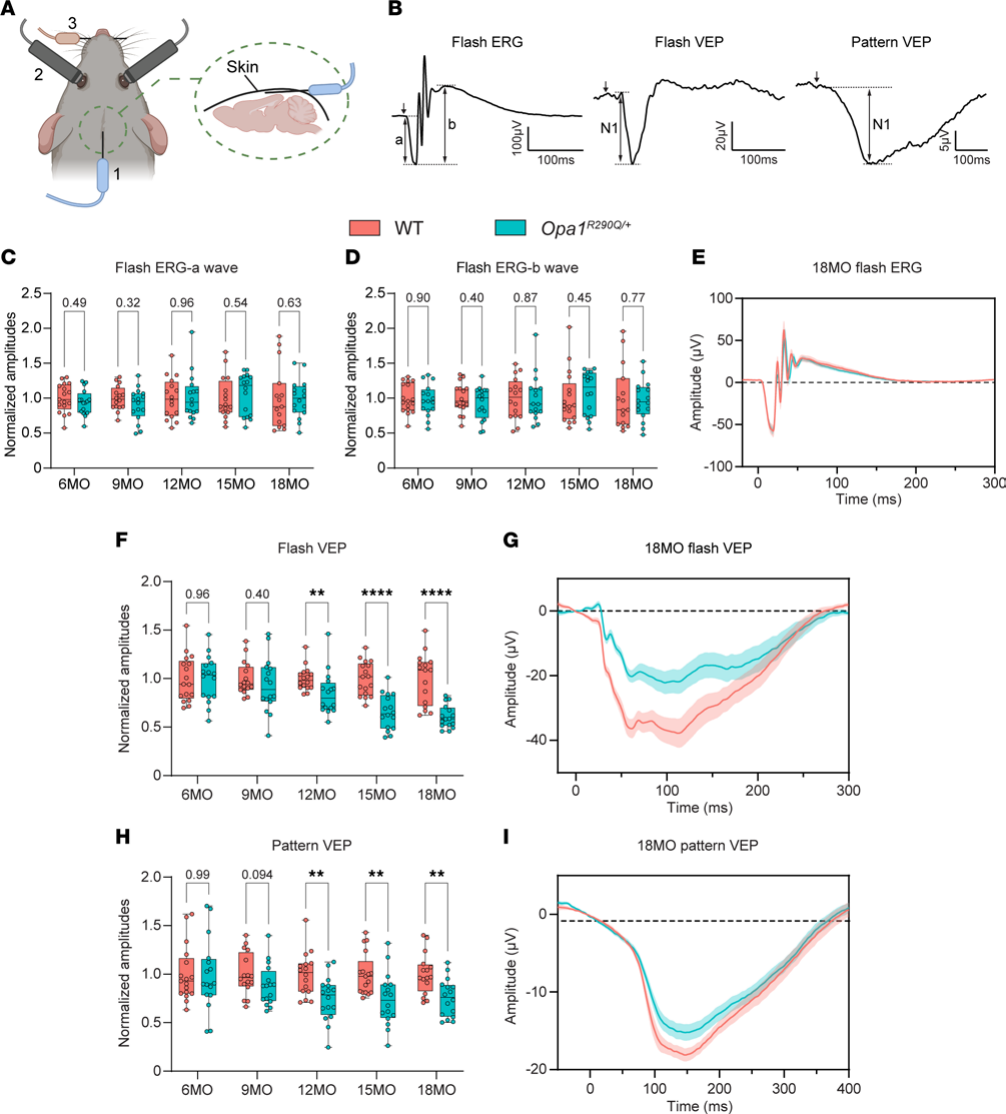
Impaired RGC function in *Opa1^R290Q/+^* mice as measured by ERG and VEP. (**A**) Diagram of recording setup: (1) subcutaneous recording electrode for VEP; (2) integrated LED stimulator and recording electrode for ERG; (3) snout reference electrode for VEPs. A ground electrode was inserted subcutaneously next to the tail (not shown). Diagram was created with BioRender. (**B**) Representative ERG and VEP traces from WT control mice. For flash ERG, the amplitudes of a-waves and b-waves were measured. For flash VEPs and pattern VEPs, the amplitude of N1 was measured. (**C** and **D**) Amplitudes of a-waves (**C**) and b-waves (**D**) of flash ERGs across ages. Each dot represents 1 animal. Amplitudes were normalized to WT average at each age. *n* = 15–17 WT and *n* = 16 *Opa1^R290Q/+^* mice per age group. (**E**) Average flash ERG traces in 18MO animals. *n* = 15 WT and *n* = 16 *Opa1^R290Q/+^* mice. (**F**) N1 amplitudes of flash VEP across age. Each dot represents 1 animal. Amplitudes were normalized to WT average at each age. *n* = 16–17 WT and *n* = 16 *Opa1^R290Q/+^* mice per age group. (**G**) Average flash VEP traces in 18MO animals. *n* = 16 WT and *n* = 16 *Opa1^R290Q/+^* mice. (**H**) N1 amplitudes of pattern VEP traces across ages. Each dot represents 1 animal. Amplitudes were normalized to the WT average at each age. *n* = 16–17 WT and *n* = 16 *Opa1^R290Q/+^* mice per age group. (**I**) Average pattern VEP traces in 18MO animals. *n* = 16 WT and 16 *Opa1^R290Q/+^* mice. Box plots (**C**, **D**, **F**, and **H**) denote minimum, first quartile, median, third quartile, and maximum values. Data in (**E**, **G**, and **I**) indicate the mean ± SEM. **P < 0.01 and ****P < 0.0001, by Mann-Whitney *U* test for each age group. All data indicate the mean ± SEM.

To assess whether RGC degeneration leads to vision deficits in *Opa1^R290Q/+^* mice, we performed longitudinal recordings of dark-adapted electroretinograms (ERGs) and visual evoked potentials (VEPs) in a large cohort of mice (*n* = 16 per genotype: 8 males and 8 females). Two LED stimulators with built-in electrodes were placed in close contact with the eyes of anesthetized mice to deliver light stimuli and record ERGs from the cornea. A subcutaneous needle electrode was positioned along the midline above the visual cortex to capture VEPs, while a reference electrode was placed in the snout and a ground electrode was inserted under the skin near the tail (Figure 3A). This setup allowed for noninvasive, simultaneous recordings of ERGs and VEPs in the same cohort of mice as they aged (example responses are shown in Figure 3B).

For ERGs, light flashes evoke responses from a mixed neuronal cell population in the retina, excluding RGCs. The response waveform is characterized by a negative a-wave, followed by oscillations and a positive b-wave (62, 63). The a-wave is generated by photoreceptors, whereas the b-wave, measured from trough to peak, is primarily produced by bipolar cells, with potential contributions from Müller glia (63, 64) (Figure 3B). The amplitudes of both the a-wave and b-wave of the flash ERG did not differ between WT and *Opa1^R290Q/+^* mice at any of the ages examined (Figure 3, C and D, and Supplemental Figure 4, D and E). The average flash ERG traces in 18MO animals were nearly identical in the 2 genotypes (Figure 3E), consistent with the selective degeneration of RGCs in ADOA.

VEPs originate from the visual cortex and depend on the ability of RGCs to transmit electrical signals to the cortex via the geniculo-cortical pathway. As such, VEPs are widely used to assess RGC connections to the brain (65–67). We measured VEP responses using full-field flash stimuli (flash VEP) and patterned stimuli (pattern VEP) consisting of alternating horizontal bars, which is more sensitive than flash VEP to lesions in the optic nerve (68). Each type of stimulus produces a stereotypical waveform with a negative peak N1 (Figure 3B). Quantification of the N1 amplitudes revealed no differences between WT and *Opa1^R290Q/+^* mice at or before 9 months of age, suggesting normal visual development and function in the *Opa1*-mutant mice. However, starting at 12 months, the mutant mice showed decreased responses (Figure 3, F and H). By 18 months, flash VEP N1 amplitudes were reduced to 61% of WT levels, and pattern VEP N1 amplitudes were 75% of WT levels (Figure 3, F and H, and Supplemental Figure 4, F and G). The average response traces also showed clear separation between 18MO WT and *Opa1^R290Q/+^* mice (Figure 3, G and I). The timing of VEP decline closely aligned with the trajectory of RGC loss and optic nerve degeneration (Figure 2, C and E). Collectively, these data suggest that RGCs in *Opa1^R290Q/+^* mice began to degenerate detectably around 9 months of age, leading to vision defects starting at 12 months.

To determine whether RGC degeneration leads to behavioral deficits, we assessed visual function using the optomotor reflex (OMR) assay. Mice were placed in a computer-monitored arena surrounded by screens displaying moving sine wave gratings at varying spatial frequencies and contrasts, while reflexive head tracking of the gratings was automatically quantified (qMOR, PhenoSys). However, we did not detect significant changes in OMR responses to varying spatial frequencies or contrasts regardless of the age of the mice, even in 18MO *Opa1^R290Q/+^* mice (Supplemental Figure 6). OMR is primarily mediated by ON direction-selective RGCs (ON DSGCs), which project to the accessory optic system (AOS) (69–71) and may constitute only approximately 10% of the total RGC population in the mouse retina (72). Given that OMR in response to vertical gratings likely depends on an even smaller subset of ON DSGCs that respond to temporal-to-nasal motion, it is plausible that this subset is not part of the degenerating population of RGCs, or that too few of them are lost to alter the behavioral response.

### Opa1^R290Q/+^ mice exhibit altered compound action potentials in the optic nerve.

**Figure 4:**
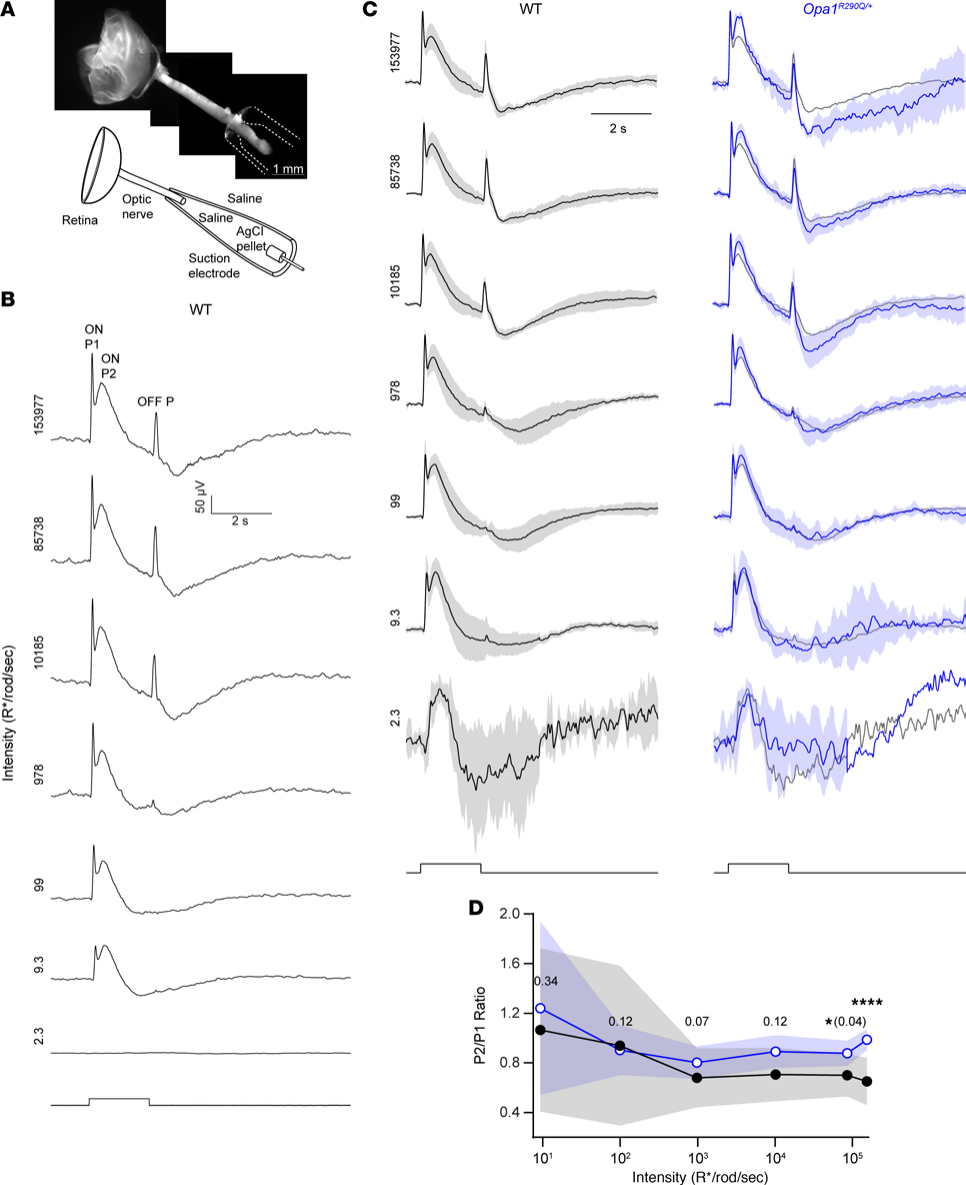
Abnormal CAPs in the optic nerve of *Opa1^R290Q/+^* mice. (**A**) Top: The recording configuration contains the retina (left) and optic nerve (center), with the latter drawn into an electrode. Bottom: Schematic showing the retina, nerve and electrode. (**B**) Example CAPs from a WT retina. For lower intensities (<10 R*/rod/s), at least 3 trials were typically averaged to increase signal/noise; for brighter intensities, 1 trial sufficed. The stimulus monitor trace is shown at the bottom, with light intensities increasing from bottom to top (indicated on the left in units of R*/rod/s). (**C**) CAPs (data indicate the mean ± SD) of WT (left, black) and *Opa1^R290Q/+^* (right, blue) retinas. Responses of each retina were normalized to its maximum value at each light intensity. The normalized *Opa1^R290Q/+^* traces (blue) were superimposed on the mean of the normalized WT traces (gray) for comparison. Shorter trials were used for dimmer stimuli, leading to a lack of error bars in some intervals. *n* = 7 WT and *n* = 7 *Opa1^R290Q/+^* retinas per age group, except for *n* = 3 *Opa1^R290Q/+^* retinas at the highest intensity. (**D**) Ratio of second and first ON peaks (ON P2/P1) from **C**. The mean ± SD is shown for WT (black) and *Opa1^R290Q/+^* (blue). For the dimmest intensity, distinct peaks were not evident (average *z* score <10) and were therefore not included in the analyses. *P < 0.05 and ****P < 0.0001, by Mann-Whitney *U* test with bootstrapping (see Methods).

To examine RGC function in a more direct and controlled manner, we isolated the retina and attached optic nerve from 20–21MO animals, delivered light to the retina, and recorded signals from the cut end of the nerve using a suction electrode (Figure 4A). These signals are understood to reflect the summed action potentials of RGCs: the compound action potential (CAP) (Supplemental Figure 7A) (73). To our knowledge, light-evoked CAPs have not been reported for the ex vivo optic nerve of the mouse. We verified by pharmacology that the CAPs originated from action potentials of RGCs driven by rods and cones (Supplemental Figure 7B).

To evaluate the effect of *Opa1^R290Q/+^* on the generation and propagation of CAPs, we delivered pulses of light (2 s duration) that evoked activity during light onset (ON responses) and offset (OFF responses) across a range of light intensities (Figure 4B, see also Methods). In WT mice, the response to low intensity light generally showed a positive peak at the onset of the light pulse (ON P1), followed by an undershoot that outlasted the step and then returned to baseline (Figure 4B). Responses to higher intensities showed 2 successive ON response peaks (ON P1 and ON P2), which developed into an undershoot during the pulse (see Methods for details). The pulse offset triggered a positive peak (OFF P) that was often followed by an undershoot before returning to baseline. The peaks were consistent with elevated action potential firing of ON, OFF, and/or ON/OFF RGCs.

To compare responses between WT and *Opa1^R290Q/+^* mice (Figure 4C), we focused on relative rather than absolute response amplitudes to control for variations caused mainly by differences in the seal between the electrode and the nerve (73). The genotypes differed at higher light intensities in the relative amplitude of the first and second ON peaks (ON P1 and P2). At the highest intensity tested, the ON P2/P1 ratios were 0.65 ± 0.19 and 0.99 ± 0.083 for WT and *Opa1^R290Q/+^* mice, respectively (Figure 4D, mean ± SD, *n* = 7 and 3 retinas, *P* < 10^–5^, effect size of 1.96 by Cohen’s *d*). The larger ratio in *Opa1^R290Q/+^* retinas could be due to a number of factors, including RGCs having slower, more dispersed responses (e.g., from poorer conduction in axonal degeneration), RGCs having more sustained responses (e.g., changes in excitability may accompany degeneration), fewer RGCs with faster kinetics, and/or more RGCs with slower kinetics (74, 75). Therefore, the altered CAPs in *Opa1^R290Q/+^* retinas were consistent with the observed RGC loss and optic nerve degeneration in these mice.

### Sarm1 KO prevents RGC degeneration in Opa1^R290Q/+^ mice.

SARM1, the key executor of Wallerian degeneration, has been shown to mediate neurodegeneration in response to mitochondrial damage (41–43, 76). *Sarm1* KO has also been demonstrated to provide protection against retinal degeneration in glaucoma (77). We therefore speculated that SARM1 might also drive RGC degeneration in *Opa1^R290Q/+^* mice and sought to determine whether *Sarm1* KO (78) could protect against RGC death in *Opa1^R290Q/+^* mice. To this end, we built a large mouse cohort consisting of 3 genotypes: (a) *Opa1^+/+^ Sarm1^–/+^* mice as controls to establish baselines; (b) *Opa1^R290Q/+^ Sarm1^–/+^* mice, expected to exhibit RGC degeneration similar to *Opa1^R290Q/+^* single mutants; and (c) *Opa1^R290Q/+^ Sarm1^–/–^* mice, in which potential rescue effects could be assessed. We followed these mice from 9 to 21 months of age, dissected retinal whole mounts every 3 months, and stained for the RGC marker RBPMS and the cell death marker p-H2Ax, as in Figure 2, A–C.

**Figure 5:**
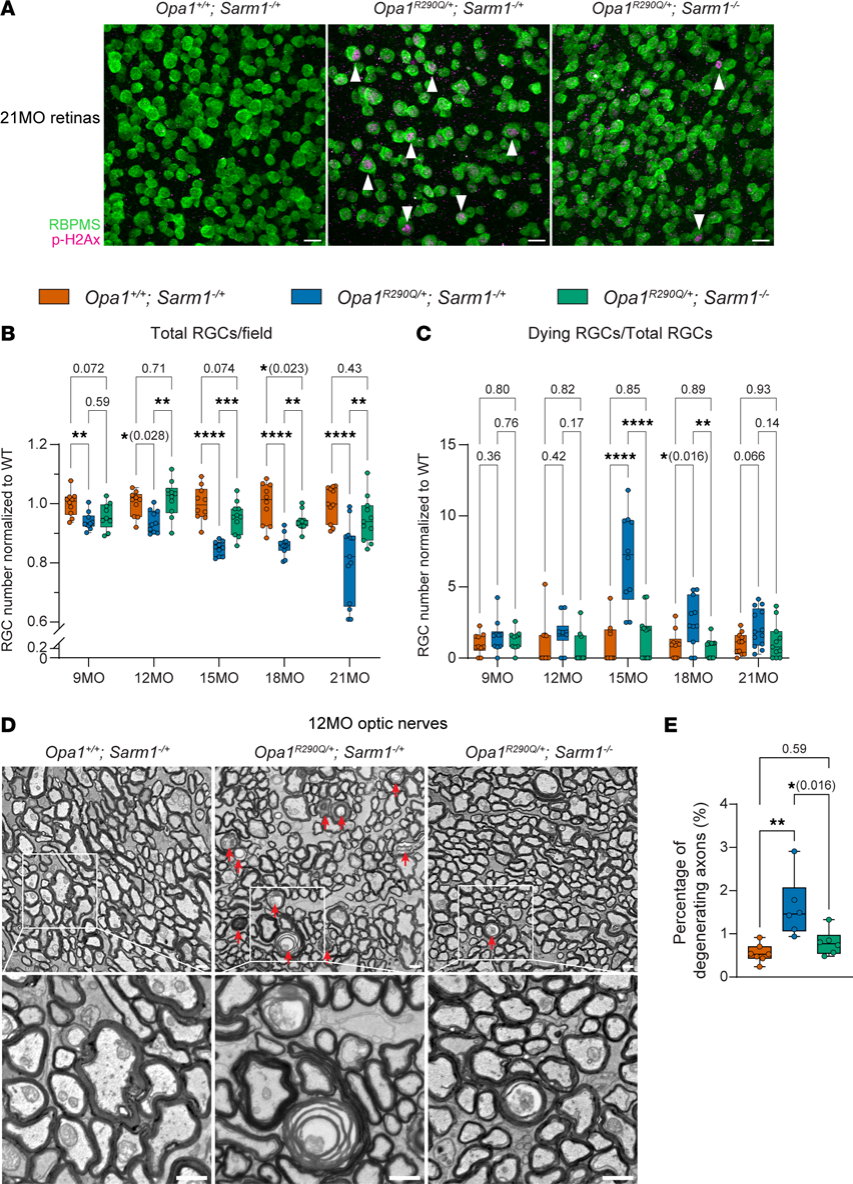
*Sarm1* KO rescues RGC and optic nerve degeneration in *Opa1^R290Q/+^* mice. (**A**) Representative images of retinal whole mounts. Arrowheads indicate p-H2Ax^+^ dying RGCs. Scale bars: 20 μm. (**B**) Quantification of total RGCs across ages. Each dot represents 1 retina. RGC counts were first averaged across the 4 quadrants of each retina and then normalized to the WT average at each age. *n* = 10–11 *Opa1^+/+^ Sarm1^–/+^*, *n* = 10–13 *Opa1^R290Q/+^ Sarm1^–/+^*, and *n* = 9–11 *Opa1^R290Q/+^ Sarm1^–/–^* retinas per age group. (**C**) Quantification of degenerating RGCs across ages in the same cohort as in **B**. Each dot represents 1 retina. The counts of dying RGCs were averaged across the 4 quadrants of each retina and divided by the average RGC number of those quadrants. This value was then normalized to the WT average at each age. (**D**) Representative EM images of cross-sections of optic nerves. Arrows indicate degenerating RGC axons. Boxed areas are enlarged in lower panels. Scale bars: 1 μm. (**E**) Percentage of degenerating RGC axons quantified from EM images from 12MO animals. *n* = 7 *Opa1^+/+^ Sarm1^–/+^*, *n* = 6 *Opa1^R290Q/+^ Sarm1^–/+^*, and *n* = 6 *Opa1^R290Q/+^ Sarm1^–/–^* optic nerves. Box plots denote minimum, first quartile, median, third quartile, and maximum values. *P < 0.05, **P < 0.01, and ****P < 0.0001, by 1-way ANOVA followed by Tukey’s multiple-comparison test for each age group.

In 21MO *Opa1^R290Q/+^ Sarm1^–/+^* mice, we found that total RGC numbers were decreased and that dying RGCs were more abundant than in controls. Both of these changes were rescued to a remarkable extent by *Sarm1* KO, even at 21 months of age (Figure 5A), and the rescuing effect was apparent throughout the longitudinal study (Figure 5, B and C). Even at 15 months, when dying cells were most apparent in the *Opa1^R290Q/+^ Sarm1^–/+^* group, the *Opa1^R290Q/+^ Sarm1^–/–^* group remained indistinguishable from the control group (Figure 5C). We also examined the optic nerve by EM in 12MO animals, when axon degeneration was prominent in *Opa1* mutants (Figure 2E and Figure 5D). Whereas the percentage of degenerating axons in *Opa1^R290Q/+^ Sarm1^–/+^* mice was increased compared with the controls, axonal degeneration was largely rescued by *Sarm1* KO (Figure 5E).

### Sarm1 KO rescues the age-dependent decline in RGC function.

To examine whether the preservation of RGCs in *Sarm1*-KO mice also preserved RGC function, we performed longitudinal ERG and VEP recordings in a separate cohort of mice of the same 3 genotypes. At all ages examined (4, 12, 15, and 18 months), we observed no differences in the amplitudes of the a-wave and b-wave in flash ERGs (Supplemental Figure 8, A–C), confirming that photoreceptors, bipolar cells, and Müller glial cells were unaffected by the *Opa1^R290Q/+^* mutation.

**Figure 6:**
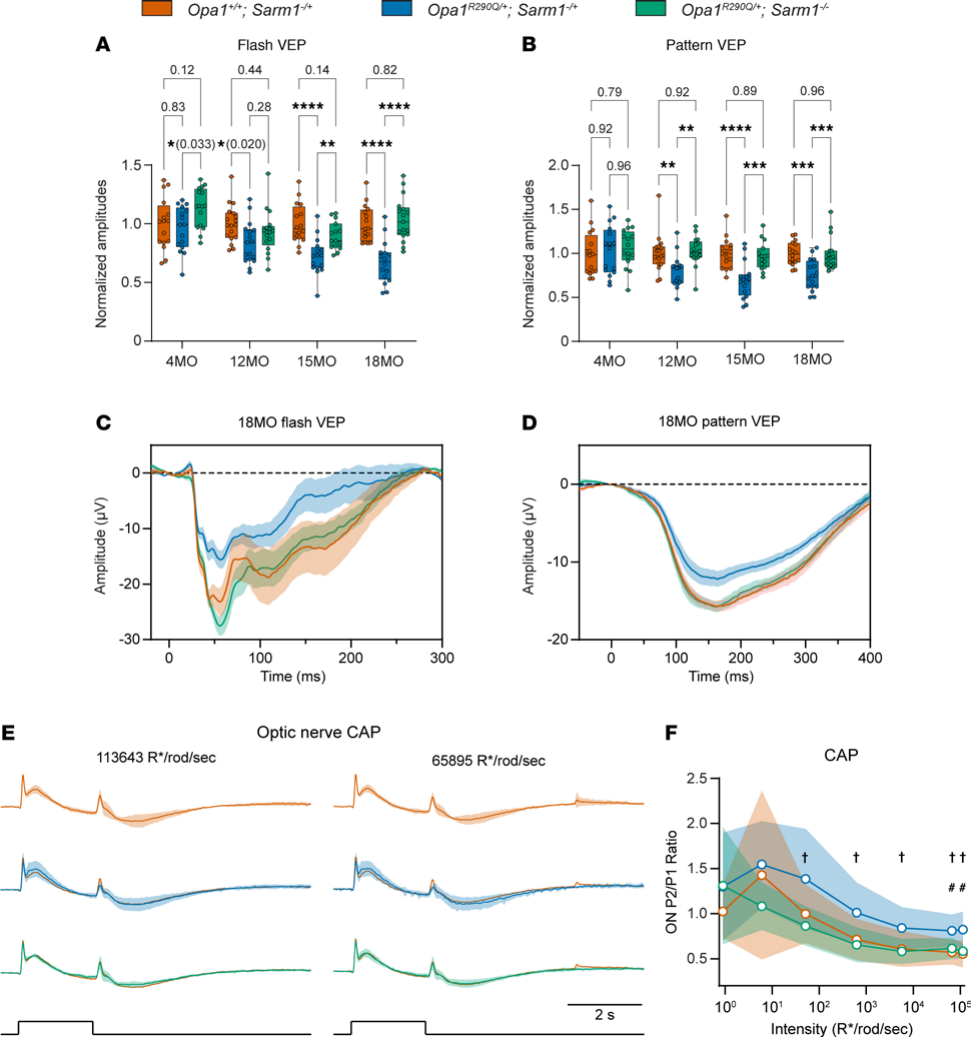
*Sarm1* KO rescues the decline in RGC function in *Opa1^R290Q/+^* mice. (**A** and **B**) Quantification of N1 amplitudes of flash VEPs and pattern VEPs measured at the indicated ages of the cohorts. Each dot represents 1 animal. Amplitudes were normalized to the WT average at each age. *n* = 15–18 *Opa1^+/+^ Sarm1^–/+^*, *n* = 16–17 *Opa1^R290Q/+^ Sarm1^–/+^*, and *n* = 15–17 *Opa1^R290Q/+^ Sarm1^–/–^* mice per age group. One-way ANOVA followed by Tukey’s multiple-comparison test. Box plots denote minimum, first quartile, median, third quartile, and maximum values. (**C** and **D**) Average flash VEP and pattern VEP traces in 18MO animals. Data indicate the mean ± SEM. (**E**) Normalized CAP traces from the 3 genotypes at the 2 highest light intensities. Data indicate the mean ± SD. Stimulus monitor traces are shown at the bottom. The *Opa1^+/+^ Sarm1^–/+^* traces were superimposed onto the other 2 groups for comparisons. *n* = 4–5 *Opa1^+/+^ Sarm1^–/+^*, *n* = 8–9 *Opa1^R290Q/+^ Sarm1^–/+^*, and *n* = 7 *Opa1^R290Q/+^ Sarm1^–/–^* retinas. (**F**) Ratio of the second and first ON peaks (ON P2/P1) as a function of light intensities from **E**. Data indicate the mean ± SD. Mann-Whitney *U* test with bootstrapping (see Methods). *P* values for all comparisons are presented in the Supporting Data Values file. *P < 0.05, **P < 0.01, ***P < 0.001, and ****P < 0.0001; ^#^*P* < 0.05 between *Opa1^+/+^ Sarm1^–/+^* and *Opa1^R290Q/+^ Sarm1^–/+^* retinas; and ^†^*P* < 0.05 between *Opa1^R290Q/+^ Sarm1^–/+^* and *Opa1^R290Q/+^ Sarm1^–/–^* retinas.

At 4 months of age, prior to any signs of RGC degeneration and consistent with normal retinal development in these mice, the 3 groups did not differ significantly in VEP responses, except for a slightly higher response in the *Opa1^R290Q/+^ Sarm1^–/–^* group compared with the *Opa1^R290Q/+^ Sarm1^–/+^* group (Figure 6, A and B). Starting at 12 months and persisting until 18 months, we found that flash VEP and pattern VEP N1 amplitudes decreased in the *Opa1^R290Q/+^ Sarm1^–/+^* mice. Remarkably, *Sarm1* KO rescued the VEP responses to control levels at nearly all ages examined (Figure 6, A and B). Even at 18 months when the R290Q mutation produced the strongest decrease in this functional assay, the flash VEP and pattern VEP response traces in the *Opa1^R290Q/+^ Sarm1^–/–^* group were indistinguishable from those in the *Opa1^+/+^ Sarm1^–/+^* group (Figure 6, C and D).

We also recorded CAPs in a randomly chosen subset of this mouse cohort at 19–21 months of age. To enhance signal resolution, we designed and used a new electrode that increases the resistance between the segment of recorded nerve and the bath (see Methods). As expected, the ON P2/P1 ratio differed between *Opa1^+/+^ Sarm1^–/+^* and *Opa1^R290Q/+^ Sarm1^–/+^* mice at high intensities (at the highest intensity, the ratio was 0.55 ± 0.15 and 0.82 ± 0.20, respectively *P* = 0.028, effect size = 1.46, *n* = 4 and 8 retinas) (Figure 6E shows the 2 highest intensities, and Supplemental Figure 8D shows the lower intensities). The ON P2/P1 ratio was rescued in the *Opa1^R290Q/+^ Sarm1*^–/–^ mice (0.58 ± 0.08, *P* = 0.014, effect size = 1.54, *n* = 7 and 8 retinas), and the average response in *Opa1^R290Q/+^ Sarm1*^–/–^ mice was indistinguishable from that of the control mice (Figure 6, E and F). Thus, the CAP data support a protective effect from loss of *Sarm1*.

### SARM1 is present in the IMS and IMM.

**Figure 7:**
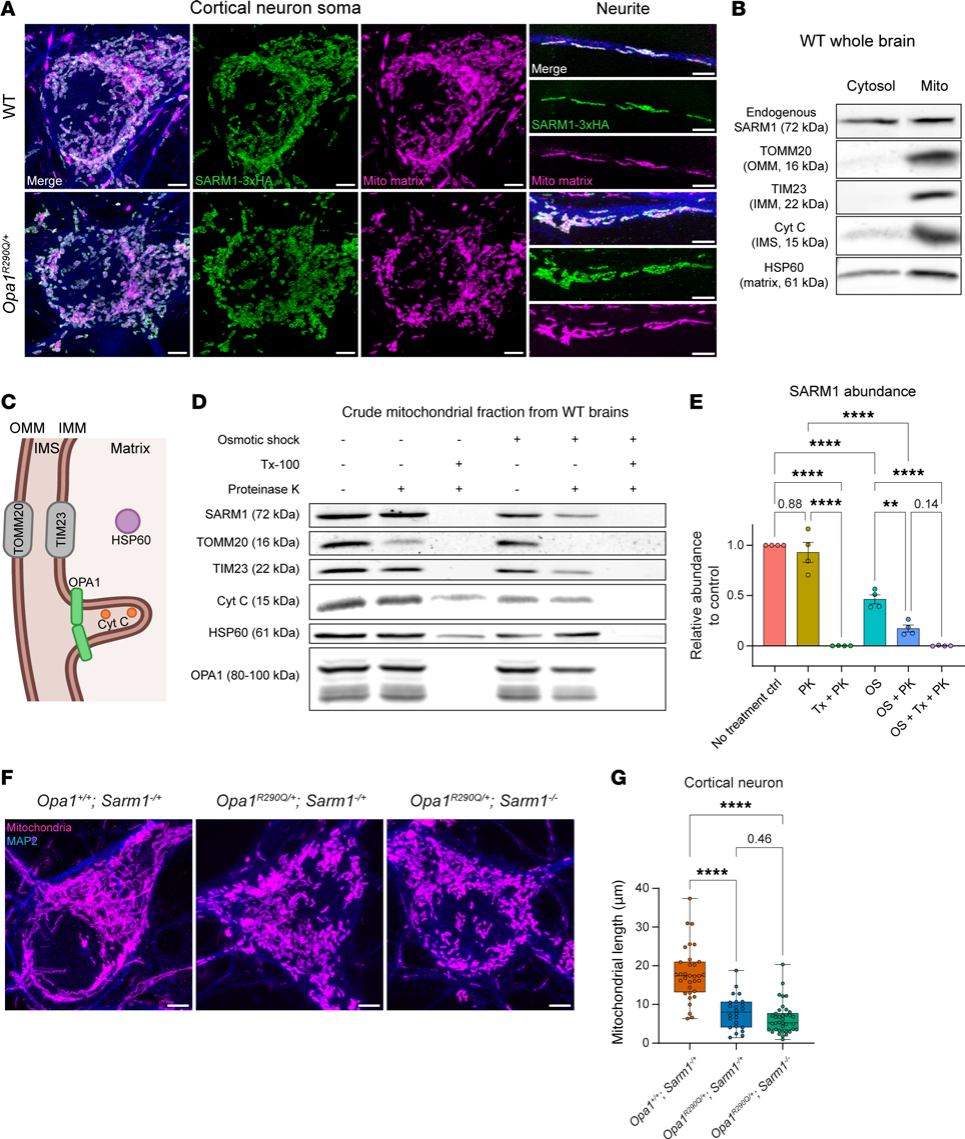
SARM1 is present in the mitochondrial IMS and IMM, and its KO does not rescue mitochondrial fragmentation. (**A**) Confocal images of overexpressed SARM1 and the mitochondrial matrix marker MitoDsRed in cortical neurons show mitochondrial localization of SARM1 in both WT and *Opa1^R290Q/+^* neurons. MAP2 staining is shown in blue. Scale bars: 10 μm. (**B**) Western blot of SARM1, TOMM20, TIM23, cytochrome C (Cyt C), and HSP60 in the cytosolic and crude mitochondrial fractions from WT whole brain tissues. (**C**) Diagram depicting localization of the marker proteins across different mitochondrial compartments. (**D**) PK protection assay on crude mitochondrial fractions from WT whole brain samples. (**E**) Quantification of SARM1 abundance across 6 conditions. *n* = 4 mice from 4 experiments. Protein levels were normalized to the control condition in each experiment. Data indicate the mean ± SEM. (**F**) Representative confocal images of mitochondria in expanded cortical neurons (DIV8–DIV9) isolated from *Opa1^+/+^ Sarm1^–/+^*, *Opa1^R290Q/+^ Sarm1^–/+^*, and *Opa1^R290Q/+^ Sarm1^–/–^* mice. Mitochondria are labeled with MitoDsRed. Scale bars: 10 μm. (**G**) Quantification of mitochondria length in *OPA1 Sarm1* cortical neurons shows no rescue of fragmentation by *Sarm1* KO (*n* = 33 *Opa1^+/+^ Sarm1^–/+^*, 24 *Opa1^R290Q/+^ Sarm1^–/+^*, and 38 *Opa1^R290Q/+^ Sarm1^–/–^* neurons from 4 experiments). Box plots denote minimum, first quartile, median, third quartile, and maximum values. **P < 0.01 and ****P < 0.0001, by 1-way ANOVA with Tukey’s multiple-comparison test (**E** and **G**). Tx, Triton X-100.

Our data suggest a model in which SARM1 became activated in the *Opa1*-mutant mice to trigger RGC degeneration. SARM1 expression levels, measured by RT-qPCR and Western blotting, were comparable between WT and *Opa1^R290Q/+^* mice at both 5 and 24 months of age (Supplemental Figure 9), indicating that its activation was not driven by increased expression but rather by metabolic changes from mitochondrial damage that relieved its autoinhibition. In this ADOA model, as in other contexts of mitochondrial dysfunction (38–40), SARM1 activation may be facilitated by its mitochondrial localization. The N-27 amino acids (S27) of SARM1 form a noncanonical mitochondrial targeting sequence capable of lipid binding (79). In HEK 293T cells, S27 is sufficient to localize EGFP inside mitochondria (79, 80). To examine SARM1 localization in neurons, we first induced overexpression of SARM1-3×HA in cultured cortical neurons and visualized it using expansion microscopy. We observed that overexpressed SARM1 localized predominantly to mitochondria in both the soma and the neurite, with no other cellular compartments detectable above background (Figure 7A), consistent with previous reports (78, 79). This pattern remained unchanged in *Opa1^R290Q/+^* neurons, despite fragmented mitochondria (Figure 7A). To verify the mitochondrial localization of endogenous SARM1, we extracted crude mitochondrial fractions from WT mouse whole brain tissues and examined SARM1 localization using a specific monoclonal anti-SARM1 antibody (Supplemental Figure 10A) (81). While SARM1 was abundant in the cytosolic fraction, a portion (~26%) was present in the mitochondrial fraction (Figure 7B). Next, we investigated whether the activation state of SARM1 altered its mitochondrial localization. To inactivate SARM1, we induced overexpression of a DN-SARM1 construct (82) in WT cortical neurons; DN SARM1 still associated with mitochondria (Supplemental Figure 11A). To activate SARM1, we treated WT cortical neurons with carbonyl cyanide m-chlorophenyl hydrazone (CCCP) to induce SARM1-dependent neuronal death (42). Compared with the DMSO control, 60 minutes of 50 μM CCCP treatment led to severe neurite loss and soma rounding (Supplemental Figure 11B). We therefore fixed neurons after 30 minutes of CCCP treatment and found that SARM1 localization to mitochondria was unchanged, despite CCCP-induced mitochondrial fragmentation (Supplemental Figure 11, C and D). Together, these results indicate that a fraction of endogenous SARM1 was associated with mitochondria in neurons, and this localization was independent of its activation state.

Despite SARM1’s mitochondrial targeting sequence, it remains controversial whether endogenous SARM1 in neurons is on the OMM, within the IMS, associated with the IMM, or inside the matrix (79, 80, 83). To clarify this, we conducted a proteinase K (PK) protection assay on crude mitochondrial fractions extracted from WT whole brains (Figure 7, C and D) (84). In the untreated control condition, endogenous SARM1, along with for translocase of outer mitochondrial membrane 20 (TOMM20), translocase of inner mitochondrial membrane 23 (TIM23), OPA1 (IMM), cytochrome C (IMS), and HSP60 (matrix), were all enriched on mitochondria. After a 1-hour PK digestion at room temperature, TOMM20, located on the OMM facing the outside space, was largely degraded. Notably, SARM1 and the other markers were well preserved in the PK-alone condition, indicating that SARM1 was protected by the OMM (Figure 7, D and E, and Supplemental Figure 10, B–F). Consistent with this, the addition of Triton disrupted mitochondrial membranes, exposing all the marker proteins, including SARM1, to PK treatment.

To further pinpoint the localization of SARM1, we performed osmotic shock (OS) to rupture the OMM and collected the mitoplasts and broken OMM by centrifugation (85). As expected, OS treatment reduced cytochrome C, which was released from the IMS upon OMM rupture (Figure 7D and Supplemental Figure 10D). Notably, SARM1 levels were reduced by 54% with OS treatment alone (Figure 7E), indicating that much of the mitochondria-localized SARM1 was freely floating in the IMS. OS slightly, but not compellingly, decreased other markers, including TIM23 and OPA1, which are integral IMM proteins (Supplemental Figure 10, C and F). When PK was added after OS, SARM1, TIM23, and OPA1 levels were considerably degraded, while cytochrome C and HSP60 levels remained unchanged (Figure 7D and Supplemental Figure 10, C–F). The further reduction of SARM1 from OS alone to OS with PK, similar to TIM23 and OPA1, suggests that a fraction of SARM1 was probably anchored to the IMM. We observed a similar response pattern of SARM1 and these markers in mitochondria purified from *Opa1^R290Q/+^* brains (Supplemental Figure 12). These results place SARM1 in 2 mitochondrial pools: one unanchored pool in the IMS and another pool anchored on the IMM, similar to the localization pattern of OPA1. However, co-IP experiments on purified mitochondria from WT mouse brains using a SARM1 antibody showed no interaction between endogenous SARM1 and OPA1, indicating an indirect link between them (Supplemental Figure 10G). Thus, mitochondria-localized SARM1 may occupy a strategic position from which to monitor and respond to secondary damage induced by OPA1 mutations, such as oxidative stress (Figure 1E and Supplemental Figure 3A).

### Sarm1 KO confers protection downstream of mitochondrial fragmentation in Opa1^R290Q/+^ neurons.

SARM1 can be activated downstream of mitochondrial dysfunctions (42, 43), and a recent study showed that SARM1 activation further exacerbates certain mitochondrial phenotypes in distal parts of neurons in a rat model of CMT2A, implying the existence of a SARM1-mitochondrial feedback loop (41). We used expansion microscopy to analyze the mitochondrial network in the somata of cortical neurons isolated from the *OPA1 Sarm1* mouse cohort (Figure 7F). We found that mitochondrial lengths in *Opa1^R290Q/+^ Sarm1^–/+^* neurons were reduced to 44% of the *Opa1^+/+^ Sarm1^–/+^* control lengths, but this fragmentation phenotype was not rescued by *Sarm1* KO (Figure 7G). Therefore, although we do not know if the feedback mechanism also occurred in our ADOA model, we conclude that *Sarm1* KO likely conferred its protection against RGC degeneration downstream of mitochondrial fragmentation induced by the *OPA1^R290Q/+^* mutation.

## Discussion

ADOA is the most common type of hereditary optic neuropathy, posing a serious challenge in health care. Despite the impact of this disease, no therapies are currently available, highlighting a critical unmet need. In this study, we present a mouse model of ADOA that revealed robust phenotypes. We identified SARM1 as a key driver of RGC degeneration in this model. On the *Sarm1*-KO background, all the key features of RGC degeneration in the *Opa1^R290Q/+^* mouse were ameliorated*,* including the number of surviving RGCs (Figure 5B), the counts of dying RGCs (Figure 5C), the profiles of degenerating axons (Figure 5E), the reductions in VEPs (Figure 6, A–D), and aberrant CAPs in the optic nerve (Figure 6, E and F). The protective effect was long-lasting, as even at 21 months of age, the *Opa1^R290Q/+^ Sarm1^–/–^* group remained indistinguishable from the *Opa1^+/+^ Sarm1^–/+^* control group. With numerous SARM1-targeting approaches already demonstrating efficacy in animal and cell models, including antisense oligonucleotides, small-molecule inhibitors, and DN-SARM1 constructs (82, 86–89), our discovery paves the way for testing these therapies for treating ADOA.

### The Opa1^R290Q/+^ allele leads to robust mitochondrial and RGC phenotypes.

The mouse model of *Opa1^R290Q/+^* mutation differs from previously generated mouse models (14–16, 19), in that it is not a simple loss-of-function allele. The mutation did not reduce overall OPA1 protein levels, but it did alter which protein isoforms were present (Supplemental Figure 1C). Although some pathogenic *OPA1* mutations, including *OPA1*^Δ58/+^, the most frequent haploinsufficiency allele in humans, do not cause mitochondrial fragmentation, the *OPA1^R290Q/+^* mutation did (46). Therefore, the R290Q mutation appears to be a more severe allele than haploinsufficiency mutations (45). In addition, its overexpression induced mitochondrial fragmentation in fibroblasts (Supplemental Figure 2), whereas overexpression of the WT protein did not. These findings indicate that the R290Q mutation possesses a DN effect that may be explained by the location of the mutation. Structural studies of the soluble form of OPA1 have shown that it forms a helical lattice on the surface of membranes independent of OPA1 GTPase activity (90, 91). The R290Q mutation, which falls within the GTPase domain, likely permits the incorporation of the mutant protein into the lattice. If the mutation decreases GTP-stimulated reorganization of the lattice, however, it may “poison” the entire lattice in a manner that a protein-null (haploinsufficient) allele would not.

The DN properties of R290Q may explain why the heterozygous mice exhibited phenotypes at multiple levels, including changes in cristae ultrastructure (Figure 1, F–M) and mitochondrial morphology (Figure 1, B and C); alterations in the glutathione redox state and mitochondrial respiration (Figure 1E and Supplemental Figure 3, A, C, and D); RGC axon degeneration and cell death (Figure 2); as well as a decline in physiological responses to visual stimuli in the optic nerve (Figure 4) and visual cortex (Figure 3). By following these phenotypes, we could describe the progression of the pathology in detail. Previous ADOA mouse models, including those with a Q285stop mutation (14), a c.1065+5G splice site mutation (15), and a c.2708_2711delTTAG model (16), all reflect haploinsufficiency and generally show a milder phenotype. Therefore, the *OPA1^R290Q/+^* mouse model is valuable from a therapeutic development perspective: if a therapy proves effective in addressing the more severe DN scenario, it is likely to be effective in the haploinsufficiency cases as well.

Strong DN *OPA1* mutations have been associated with ADOA-plus in some patients, who experience additional symptoms (12, 13). However, aside from the RGC phenotypes, the *Opa1^R290Q/+^* mice appeared superficially normal and had normal lifespans, although the potential involvement of other organs cannot be excluded.

### Timing and identity of dying RGCs.

The relatively short lifespan of the mouse can pose a challenge for modeling a slow neurodegenerative disease. In some cases, mutations that are pathogenic in humans, such as those in the PINK1 and Parkin genes that cause Parkinson’s disease, fail to cause robust neurodegeneration in mice (92–94). In patients, ADOA is usually detected within the first decade of life and progresses slowly over the next 2 decades or more (61). We were fortunate, therefore, to be able to detect strong phenotypes in the *Opa1^R290Q/+^* mouse, with RGC death and axon degeneration detectable around 9 months of age, followed by a significant net loss of RGCs and electrophysiological consequences detectable starting at 12 months. The DN properties of the R290Q mutation compared with haploinsufficiency models may contribute to the robustness of the phenotypes. The degeneration progressed slowly, such that over 80% of RGCs were still preserved at 18 months, near the end of the normal lifespan of the mice. This observation aligns with the slow progressive nature of ADOA in humans (61). The slow degeneration in the mouse model, however, still provides a useful window in which to test potential interventions; any such trials should probably be initiated within the first 6 months to effectively ameliorate disease onset.

In the *Opa1^R290Q/+^* mouse, RGC degeneration predominantly occurs between 9 and 15 months, and tapers off by 18 months (Figure 2, B and E). This pattern might be explained by differential susceptibility of RGC subtypes to mitochondrial damage: certain subtypes may be more vulnerable and degenerate within this window, while others may be more resistant, dying at a slower rate or not at all. To date, at least 46 RGC subtypes have been identified in mice, with no single subtype comprising more than 10% of the total RGC population (95). These RGC subtypes can be grouped into subclasses with specific molecular markers (95). To a large extent, these subclasses appear evolutionarily conserved across species, including humans, although the distribution of each subtype can be different (96). It has not been determined in patients with ADOA which RGC subtypes are more vulnerable to degeneration. A future direction beyond the scope of this study will be to screen RGC subtypes and identify those that preferentially undergo degeneration in the *Opa1^R290Q/+^* mouse during aging. Given that the OMR assay did not reveal strong defects in the *Opa1^R290Q/+^* mouse, it is likely that the ON direction-selective RGCs do not undergo appreciable degeneration.

### SARM1 and mitochondrial dysfunction.

SARM1 was initially identified as the executioner of Wallerian degeneration and was later shown to play an active role in the pathology of various neurodegenerative conditions, as evidenced by the protection conferred by *Sarm1* KO in mouse models (83, 97, 98). Notably, mitochondrial damage activates SARM1, although the precise mechanisms remain elusive (42). In this study, we demonstrated that SARM1 is also a key driver of age-related RGC degeneration induced by defective mitochondria in the *Opa1* mutant in vivo. Our data indicate that SARM1 is likely activated downstream of mitochondrial defects, as *Sarm1* KO did not rescue the mitochondrial fragmentation phenotype in the *Opa1*-mutant neurons (Figure 7, F and G).

SARM1 has a mitochondrial targeting sequence (S27) (79), but the significance of a mitochondrial pool of SARM1 is unclear; SARM1 can still drive degeneration even without this sequence (83). Where SARM1 resides in mitochondria has also been ambiguous. Overexpressed S27-EGFP or SARM1-EGFP localizes inside mitochondria in HEK 293T cells, as confirmed by immunogold labeling and transmission EM (79, 80). In contrast, another study reported that overexpressed SARM1-Venus associates peripherally with the mitochondrial outer membrane in cultured rat neurons (83). Using immunocytochemistry (ICC), we observed that overexpressed SARM1 predominantly localized to mitochondria, with no obvious signals in other cellular compartments. Fractionation experiments revealed that a fraction of endogenous SARM1 copurified with the mitochondrial fraction, although most remained cytosolic. This discrepancy may arise from the cytosolic portion being too diffuse to detect by ICC or from leakage of SARM1 from mitochondria during fractionation, leaving the exact size of the mitochondrial pool of SARM1 uncertain. Nevertheless, our PK protection assay clearly indicated that mitochondrial SARM1 localized to the IMS and may also have associated with the IMM (Figure 7D), where OPA1 also resides. Although SARM1 and OPA1 do not coprecipitate (Supplemental Figure 10G), we speculate that localization of SARM1 in the IMS positions it at an advantageous position to detect defects in the electron transport chain, such as the production of ROS caused by *OPA1* mutations.

Is there a specific aspect of mitochondrial damage that activates SARM1? Our data suggest that oxidative stress might play a key role. We observed a change in the glutathione redox in both fibroblasts and whole brain metabolites, which is a hallmark of oxidative stress, while ATP production and mtDNA copy number were only minimally affected. Notably, oxidative stress caused by mitochondrial toxins, rather than defective ATP production, has been proposed to activate SARM1 in cell culture models (42). This activation likely depends on the loss of (nicotinamide nucleotide adenylyltransferase 2) NMNAT2, a NAD^+^ synthase that inhibits SARM1 (43). It would be informative to determine whether SARM1 is activated through a similar mechanism downstream of oxidative stress in ADOA, e.g., by chronic administration of antioxidants and examination of NMNAT2 levels during aging.

While we demonstrated the comprehensive and enduring protection of *Sarm1* KO in ADOA, it is important to note that mice in the degeneration control group also carried a heterozygous *Sarm1* mutation. Recent studies have shown that loss of 1 copy of *Sarm1* reduces its activity by half and provides partial protection against neurodegeneration (87, 99). Since we did not include a littermate control of *Opa1^R290Q/+^ Sarm1^+/+^* in the same cohort, we could not definitively determine whether the heterozygous *Sarm1* mutation also provided protective effects against RGC degeneration in ADOA. However, RGC death in the *Opa1^R290Q/+^ Sarm1^–/+^* mice appeared to be delayed compared with RGCs in the *Opa1^R290Q/+^* single mutants in a separate cohort (Figure 2B and Figure 5C), which supports this possibility. This consideration is particularly relevant for therapy development: small-molecule inhibitors or antisense oligonucleotides, which hold great potential as SARM1-targeting therapies, are unlikely to completely inhibit SARM1 activity. Therefore, determining the dose dependence of RGC degeneration on SARM1 activity will be crucial in the drug development process.

Since the discovery of SARM1 (83, 97), there has been tremendous interest in investigating its involvement in various neurodegenerative diseases. Recent studies have provided compelling evidence that mitochondrial damage is a robust trigger for SARM1 activation and subsequent degeneration (41–43). Early research utilized cell culture systems using mitochondrial toxins (42, 43), while more recent efforts have expanded to in vivo models of mitochondrial neurodegeneration (41). Our discovery that *Sarm1* KO suppressed RGC degeneration in the ADOA mouse model further supports the mitochondrial/SARM1 axis as a critical mechanism in mitochondrial neurodegeneration. Although our work does not exclude the involvement of other degenerative pathways, particularly in the context of aging in patients with ADOA, it underscores the critical role of SARM1 in this process. Given this, we propose that SARM1’s role should also be investigated in other types of mitochondrial neurodegenerative disorders, such as Leber hereditary optic neuropathy (LHON), which is caused by mitochondrial DNA mutations and leads to RGC degeneration similar to that seen in ADOA (61, 100, 101).

## Methods

### Sex as a biological variable.

Our study examined male and female animals, and similar findings are reported for both sexes.

### Statistics.

Data points represent biological replicates. Box plots denote minimum, first quartile, median, third quartile, and maximum values. Statistical analyses were conducted using GraphPad Prism 10 (GraphPad Software). : For comparisons between 2 groups, Mann-Whitney U test was used. For comparisons between 3 groups, 1-way ANOVA followed by Tukey’s multiple-comparison test was used. For fibroblast proliferation, 2-way ANOVA with Šidák’s multiple-comparison test was used. *P* values are listed in graphs and the Supporting Data Values file. A *P* value of less than 0.05 was considered statistically significant. Data in the figures are represented as box plots, mean ± SEM, or mean ± SD, as specified in each legend.

### Study approval.

All mouse procedures were approved by the IACUC of Boston Children’s Hospital (BCH) and were conducted in accordance with NIH guidelines. Animals were group-housed at the Animal Resources at the Children’s Hospital (ARCH) facility and maintained in the environmental conditions recommended by the Association for Assessment and Accreditation of Laboratory Animal Care – International (AAALAC). Other procedures involving cell cultures, bacteria, viruses, and recombinant DNAs were approved by the BCH Institutional Biosafety Committee (BCH IBC).

### Data availability.

Values for all data points in graphs can be found in the Supporting Data Values file. Additional data related to this work may be requested from the corresponding author.

Detailed methods are included in the Supplemental Methods.

## Author contributions

CD, MTHD, and TLS conceptualized the project. CD wrote the manuscript, and TLS revised the manuscript. CD conducted the experiments, except where otherwise noted. PSN built and maintained the mouse colony, performed the OMR assay, and conducted part of the EM analyses. JG contributed to neuronal culture experiments, expansion microscopy, and EM analyses. WG contributed to generating the *Opa1^R290Q/+^* mouse. MYF conducted the cryo-ET experiments, performed data analyses, and drafted the cryo-ET results. MYF and LHC conceived of the cryo-ET experiment and developed the methodology. SRC conducted the CAP experiments, and SRW analyzed the CAP experiments. PM conceptualized and developed the CAP technique. MTHD conceptualized the new CAP electrode, designed the CAP experiments, and drafted the results of these experiments.

## Supplementary Material

Supplemental data

Unedited blot and gel images

Supporting data values

## Version 1 05/09/2025

In-Press Preview

## Version 2 06/16/2025

Electronic publication
