# Supplementary material for: SARM1 loss protects retinal ganglion cells in a mouse model of autosomal dominant optic atrophy
Source: J Clin Invest. 2025 May 9;135(12):e191315. doi: 10.1172/JCI191315 (PMC12165793; doi:10.1172/JCI191315)
Supplement: Supplemental data [file jci-135-191315-s127.pdf]

METHOD DETAILS

Mouse lines

The *Opa1*$^{R290Q/+}$ mice were generated by CRIPSR/Cas9 genome editing on the C57BL/6J background obtained from the Jackson Laboratory (JAX #000664) by the Mouse Gene Manipulation Core at BCH (see Generation of the *Opa1*$^{R290Q/+}$ mouse). All mice were used at the ages specified in the results and figures. All experiments included a balanced mixture of male and female mice in approximately equal numbers.

Primary cell cultures

HEK 293T cells (ATCC, CRL-11268) were cultured in Dulbecco's Modified Eagle Medium (DMEM) with 10% (v/v) fetal bovine serum (FBS) and 1% (v/v) penicillin-streptomycin (P/S) at 37°C with 5% CO_2. Primary fibroblasts were isolated as described (see method details) and cultured in DMEM containing 20% FBS and 1% P/S at 37°C with 5% CO_2. Both HEK 293T cells and fibroblasts were passaged at 90% confluency. Primary neurons were dissected (see **Isolation of primary neurons**) and cultured in Neurobasal (NB) medium supplemented with B27 and Penicillin-Streptomycin-Glutamine (PSG) at 37°C with 5% CO_2. The neuronal culture medium was refreshed every two days with a half-volume change.

Generation of the *Opa1*$^{R290Q/+}$ mouse

All animal research and care procedures were approved by the Boston Children's Hospital IACUC. Mice were maintained on a 12-hour light/dark cycle with food and water provided *ad libitum*. To prepare the ribonucleoprotein complex (RNP), 0.61 pmol each of *Opa1*-R290Q-crRNA and tracrRNA was conjugated and incubated with Cas9 protein (30ng/ul), according to a published protocol (102). The *Opa1*-R290Q single strand oligonucleotide donor (IDT) was then mixed (10ng/ul) with RNP to prepare the microinjection cocktail. For knock-in mouse generation, the microinjection cocktail was injected into 0.5 dpc embryos harvested after mating C57BL/6J

mice (Jackson Laboratory). Post-injection embryos were reimplanted into pseudo-pregnant CD1 foster females (Envigo) and allowed to reach term. Tail snip biopsies were collected from pups at P7 to prepare genomic DNA for screening to identify founders. Genomic DNA was amplified using NEB Q5 Hotstart DNA polymerase with the *Opa1* Genomic PCR screening primers. The PCR products were digested with EcoNI, a site induced by the R290Q mutation. Positive PCR samples were then sequenced with the *Opa1* Genomic PCR sequencing primers. Positive samples were then further validated by sequencing PCR amplicons with the *Opa1* Validation PCR primers. One female founder was obtained, and subsequent genotyping was performed using PCR with the *Opa1* genotyping primers followed by EcoNI digestion. The WT allele produces a 964 bp band, while the R290Q mutation introduces an EcoNI site, resulting in two bands at 356 bp and 608 bp. The female founder was initially outcrossed to wildtype (WT) C57Bl/6J obtained from the Jackson Laboratory (JAX #000664), followed by successive backcrossing to C57Bl/6J. The resulting $Opa1^{R290Q/+}$ mice were inbred to generate WT and $Opa1^{R290Q/+}$ littermates. The sequences of the crRNA, HDR template, screening and genotyping primers are listed in Supplemental Table 1.

Isolation of primary fibroblasts

Primary fibroblasts were isolated from mouse embryos from the same litter between embryonic day 15 (E15) and E18. Briefly, the pregnant females were euthanized by CO2, and the embryos were extracted from the uterus. After removing the heads and discarding the viscera, the remaining tissues were finely chopped with a razor blade and digested with 2.5 mL of trypsin for 5 minutes at 37°C. The tissues were then triturated with a P1000 pipette. An additional 2.5 mL of trypsin was added, and the digestion continued for another 5 minutes. 8 mL DMEM containing 20% FBS and penicillin-streptomycin (P/S) was added to terminate digestion. The tissues were then thoroughly triturated with a pipette. After allowing the suspension to settle for 2 minutes, 10 mL of the cell suspension was transferred to a 75cm^2 tissue culture flask. Fibroblasts were

cultured in DMEM containing 20% FBS and P/S, and were passaged upon reaching confluence. Cells were cryopreserved in culture media containing 10% DMSO and stored in liquid nitrogen. Low-passage cells were used for subsequent experiments.

Isolation of primary neurons

Buffers and plates

10X dissection media (DM): 100 mM MgCl2, 10 mM kynurenic acid, 100 mM HEPES in 1X HBSS, pH 7.2.

Papain Solution (10 mL): 10 mL of 1X DM, 200 µL papain, and 8 grains of L-cysteine were mixed and warmed at 37°C until the solution became clear.

Heavy Inhibitor (HI) Solution (10 mL): 8 mL of 1X DM, 80 mg BSA, and 80 mg trypsin inhibitor were combined.

Light Inhibitor (LI) Solution (20 mL): 18 mL of 1X DM was mixed with 2 mL of HI.

Culture media: NB supplemented with B27 and PSG

Plates: 12mm #1.5 coverslips were placed in 24-well plates and coated with 3.5 µg/mL laminin and 20 µg/mL poly-L-lysin dissolved in autoclaved ddH_2O for 1 hour at 37°C. The plates were then washed three times with water before plating neurons.

Dissection and culture

Cortical neurons were isolated from E18-P0 mice from the same litter. Mice were decapitated with scissors and placed in dishes containing 1X DM (10X DM diluted in HBSS), followed by removal of the skin and skull with forceps. The brains were extracted, and meninges were carefully removed. Cortices were dissected and transferred to a 15 mL Falcon tube containing 1 mL of papain solution followed by incubation at 37°C for 5min. The papain solution was then removed, and the tissue was washed with 0.5 mL of LI, repeated twice to thoroughly remove papain. This was followed by a single wash with 0.5 mL of HI. The tissue was then washed three times with 1 mL of culture medium. After the final wash, 1 mL of the culture medium was

added, and the tissue was gently triturated ten times with a P1000 pipette. The suspension was allowed to settle for 2 minutes before the cell suspension was transferred to a new tube. Cell density was determined using a hemocytometer, and 100,000 cells in 500 μL were plated per well of a 24-well plate. The media were completely replaced two hours after plating.

Plasmid construction

Plasmids were constructed using Gibson Assembly or restriction enzyme digestion followed by ligation. For lentiviral constructs, the coding sequences of mouse OPA1, MitoDsRed, mouse SARM1 were cloned into the pLVX backbone. OPA1 was also cloned into the pEGFP-N1 backbone (Addgene #54767), and the R290Q mutation was introduced using the Q5 site-directed mutagenesis kit. Templates for OPA1, MitoDsRed, SARM1, and EGFP were obtained from Addgene (plasmid #62845, #174541, #50707, and #54767 respectively). Lentiviral constructs were transformed into NEB stable competent cells, and pEGFP-N1 constructs were transformed into DH5α Competent Cells.

Lentiviral packaging

HEK 293T cells were seeded into five 15 cm dishes 24 to 48 hours prior to transfection. PEI transfection was done when cells reached 90% confluence. For one dish, 12 μg of psPax2, 5 μg of pMD2.G and 15 μg of transfer plasmid were mixed with 1 mL of DMEM (without phenol red) and 90 μL of PEI. The transfection mixture was then added to the dishes. The following day, the media were replaced with standard HEK culture media. Two days later, the media from all five dishes were combined and filtered through a 0.45 μm filter. The filtered media were centrifuged at 18,000 rpm for 2 hours at 4°C in a Beckman Coulter ultracentrifuge using a SW32Ti rotor. The supernatant was carefully removed, and the tubes were drained for 5 minutes on a paper towel. The resulting pellet was resuspended in 100 μL of PBS containing 0.001% F68. The resuspended viruses were aliquoted, flash frozen in liquid nitrogen, and stored at -80°C.

Cell transfection/transduction

Fibroblasts transfection

Fibroblasts (60,000 cells per well in 600 µL of culture media) were seeded into 24-well glass bottom plates. The following day, maxipreped plasmids were transfected using the TransIT-LT1 Transfection Reagent. For each well, 0.2–0.5 µg of DNA (OPA1 iso1-WT, OPA1 iso1-R290Q or MitoDsRed) was mixed with 50 µL of Opti-MEM serum-free media and 1.5 µL of the TransIT-LT1 reagent. The mixture was incubated at room temperature for 15 minutes before being added dropwise to each well. Cells were fixed 1 to 2 days post-transfection for immunocytochemistry.

Viral transduction

Cultured neurons (100,000 cells in 500 µL of culture media per well) were transduced with lentiviruses encoding MitoDsRed or SARM1-3×HA. 1.5 to 2 µL of viruses were added per well in 24-well plates on DIV6-7. Neurons were fixed 3-4 days post-transduction for immunocytochemistry and expansion microscopy.

Immunocytochemistry (ICC)

For regular ICC, transfected or transduced cells were fixed in 4% PFA for 12 minutes, followed by three PBS washes. Cells were then permeabilized with 0.5% Triton X-100 for 10 minutes, followed by two PBS washes. Blocking was performed using Superblock for 30 minutes. Cells were incubated with primary antibody in Superblock at 4°C overnight. The following day, Cells were washed with PBS three times and incubated with secondary antibody in Superblock for 1 hour at room temperature. After three additional PBS washes, a few drops of Vectashield mounting medium were added, and a #1.5 coverslip was placed over the cells. The cells were imaged on a Zeiss LSM 700 laser scanning confocal microscope with a 63× oil objective.

Expansion microscopy

Staining

Expansion was done following the published glutaraldehyde (GA) method (48). The staining

protocol was similar to the regular ICC protocol with the following modifications: 1) 0.1% GA was included in the fixation medium during the initial fixation step; 2) after fixation, samples were reduced in 10mM sodium borohydride for 30 minutes, followed by three PBS washes before proceeding to permeabilization; 3) after incubation with secondary antibody and subsequent PBS washes, the cells were treated with 0.25% GA for 10 minutes. Following three additional PBS washes, the cells were prepared for expansion.

Buffers for expansion

Monomer solution (10 mL): 2.25 mL of 33% w/v Sodium acrylate, 0.625 mL of 40% w/v Acrylamide, 0.75 mL of 2% w/v N, N'-Methylenebisacrylamide, 4 mL of 29.2% w/v Sodium chloride, 1mL of 10X PBS and 1.375 mL of water.

Gelling solution (200 μL): 188 μL of monomer solution, 4 μL of 10% TEMED, 4 μL of 10% Ammonium Persulfate (APS) (add just before gelation) and 4 μL of water.

Digestion buffer (50 mL): 42.5 mL of TAE buffer, 2.5 mL of 10% Triton X-100, 5 mL of 8M guanidine HCl and 1:100 dilution of Proteinase K (20mg/mL) added before use

Expansion

Immediately before expansion, the gelling solution was prepared without APS. PBS was aspirated from the wells, and 200 μL of monomer solution was added to each well for equilibration. After 1 minute, the coverslip with cells was transferred to a glass-bottom dish with cell side facing up. APS was then added to the gelling solution, and 100 μL of gelling solution was applied onto the coverslip. A 25 mm coverslip was placed on top of the gelling, creating a coverslip-gel-coverslip sandwich. After 30 minutes of gelation at room temperature, the top coverslip was carefully removed with forceps. The sample was then incubated with 1.5 mL of digestion buffer with Proteinase K for 45 minutes at 37°C. Following digestion, the gel was transferred to a 15 cm dish and allowed to expand in ddH_2O for 30 minutes, with the expansion process repeated four times (2 hours total). After expansion, the gel was trimmed into a small piece, which was placed in a glass-bottom dish with cell side facing down. The gel was then

sealed with 4 mL of 1% low-meting point agarose gel. The sample was imaged using an inverted Leica TCS SP8 laser scanning confocal microscope.

Seahorse

24,000 fibroblasts were seeded in each well of a seahorse 96-well plate and grown overnight. The next day, the culture medium was replaced with the Seahorse XF DMEM Medium pH 7.4, supplemented with 10 mM glucose, 2 mM GlutaMax and 1 mM pyruvate before the assay. Seahorse was performed on the XFe96/XF96 Analyzer (Agilent) using the default mito stress protocol, following the manufacturer's instructions. 1.5 µM Oligomycin, 2µM FCCP and 0.5 µM Rotenone and Antimycin A were added sequentially. After the seahorse assay, 80 µL of RIPA buffer with protease inhibitors was added to the wells to lyse the cells. Protein concentration was then measured using the BCA assay kit. Oxygen consumption rates were normalized by total protein levels.

Cryo-Electron Tomography

Sample preparation

Fibroblasts were detached from flasks and counted manually. Quantifoil Au 200 mesh holey carbon R2/2 grids were glow discharged for 90 s at 15 mA using a PELCO easiGlow glow discharge system (Ted 44 Pella). At room temperature, 3 µL of sample at a concentration of $1*10^6$ cells/mL were deposited onto grids. After 30 seconds, grids were back-blotted with filter paper for 15 seconds before being plunged into liquid ethane. Subsequent grid handling and transfers were done under liquid nitrogen conditions. Grids were clipped using cryo-FIB autogrids (Thermo Fisher Scientific).

Preparation of lamellas for cryo-ET

Grids were loaded two at a time into an Aquilos2 (TFS). After loading, grids were sputter coated with inorganic platinum in a cryo-FIB chamber, then using a GIS were coated in organometallic platinum and then grids were sputter coated with inorganic platinum again to protect and

prevent uneven thinning of the sample. Lamella were milled using two rectangular patterns with a gallium ion-beam. Trenches were milled to create macro-expansion joints to improve the stability of the lamella. Tilt angles used for milling ranged from 7°-10°. Lamellas were milled at decreasing ion beam currents from 1 nA to 10 pA resulting in lamellae that were 150-200 nm thick.

Cryo-ET data collection and processing

Data were collected using an automated data collection software, Thermo Fisher Scientific Tomography 4, on a FEI Titan Krios equipped with an energy filter (20 eV slit width) at 300 keV and a Gatan K3 direct detector. Images were collected using a defocus range of -3.5 to 4.5 μm using super resolution mode at a calibrated pixel size of 1.375 Å/pixel. Tilt range and dose rate varied depending on prepared lamella, but, generally, collections ranged from -65° to 65° in 2° step increments following the Hagen dose-symmetric scheme (103). Using counting mode, 910 to 960 ms tilt images were collected with a frame rate of 152-160 ms and dosage of 0.556-0.602 e/Å per frame accumulated to a target total dose of 170-200 e/Å. Acquired tilted movies were motion corrected and Fourier cropped to a pixel size of 2.75Å/pixel using Relion v.5.0b-3_cu11.8 (104) to generate dose-weighted micrographs. These micrographs were subjected to contrast transfer function (CTF) refinement (CTFFIND4 (105)) and tilt-series were aligned using AreTomo (106). CTF-corrected tomograms were reconstructed in Relion5 and denoised with either IsoNET (107) or cryoCARE (108,109). Summed projection images of cryo-tomogram slices were performed in *Dynamo* (110).

Segmentation of tomograms

For segmentation purposes, tomograms were first denoised with cryoCARE (108,109) and then membranes were boosted using with IsoNet (107). In general, the following inputs were used for IsoNet: snrfalloff 0.7; deconvstrength 0.7; density_percentage 50; std_percentage 50. Membranes were then segmented with Membrain-seg (111) using the provided pretrained model. Segmentations were cleaned up and membrane labels were assigned in Amira (TFS).

Quantification of cryo-electron tomograms

Quantitative and qualitative analysis of cristae morphology: Cristae were classified as lamellar (sheet or flat), tubular (straw), invagination (short crista with a CJ), ring, globular (balloon), and undetermined (shape could not be determined).

Analysis of membrane architecture: Segmentations of wild-type (n=19) and mutant (n=22) mitochondria from Membrain-seg (111) with assigned labels were used as input for surface morphometrics (57). Features extracted were OMM-IMM distance, cristae angle relative to the OMM, and membrane curvedness.

Tissue preparation

Mice at the desired age were euthanized by isoflurane overdose in a bell jar. Transcardial perfusion was immediately performed using a peristaltic pump. Mice were perfused with 25 mL of ice-chilled PBS, followed by 25 mL of 4% PFA in PBS for retinal histology or 2.5% formaldehyde and 2.5% glutaraldehyde in 0.1M sodium cacodylate (pH 7.4) for electron microscopy (EM) at a low rate of 5 mL/min. For retinal histology, the eyeballs were enucleated, a hole was made in the center of the cornea with a needle, and the eyeballs were post-fixed with 4% PFA for one hour before retinal dissection. For EM, the skull and brain were removed, and the optic nerves were dissected and postfixed in 2.5% formaldehyde and 2.5% glutaraldehyde in 0.1M sodium cacodylate (pH 7.4) overnight before proceeding to EM processing.

Retinal histology

Fixed eyeballs were transferred to a dish containing TBS. The cornea was removed with dissection scissors, and the lens and vitreous were discarded. The sclera was then peeled away from the retina. Four evenly spaced cuts were made on the isolated retina to allow it to flatten. The retinas were blocked overnight in a 96 well plate with TBS (pH 7.6) containing 5% normal donkey serum (NDS), 1% BSA and 1% Triton X100. The next day, the blocking solution was

replaced with the primary antibody solution containing 5% NDS, 0.2% BSA, 0.5% Triton X-100, guinea pig anti-RBPMS antibody (Novus Biologicals or PhosphoSolutions), and rabbit anti-phospho-H2Ax (Cell Signaling) in TBS. The retinas were incubated with the primary solution for three days on a shaker at 4°C. After incubation, the retinas were washed four times with TBS for 30 minutes each. The retinas were then incubated overnight with the secondary antibody solution, which had the same buffer composition as the primary antibody solution, and included Hoechst, Alexa 488-conjugated donkey anti-guinea pig antibody (Jackson ImmunoResearch) and Alexa 568-conjugated goat anti-rabbit antibody (Invitrogen). The following day, the retinas were washed four times with TBS, mounted on slides with 50 µL of Vectashield mounting media, covered by a #1.5 cover glass, and sealed with nail polish. The retina samples were imaged using a Zeiss LSM 700 laser scanning confocal microscope with a 25× oil objective. For each retina, z stacks of images (256 µm x 256 µm) were captured from each quadrant approximately 1.2 mm from the optic nerve head.

Electron microscopy of optic nerves

Mice were perfused as previously described with 2.5% formaldehyde and 2.5% glutaraldehyde in 0.1M sodium cacodylate (pH 7.4). The optic nerves, prior to the chiasm, were dissected, cut into two pieces, and post-fixed overnight in the same fixation buffer. The next day, the optic nerves were processed at the EM core at Harvard Medical School. The optic nerves were washed in 0.1M cacodylate buffer and post-fixed with 1% Osmium tetroxide (OsO_4)/1.5% potassium ferrocyanide ($KFeCN_6$) for 1 hour, followed by two washes in water. The samples were then washed once in 50mM maleate buffer (pH 5.15, MB) and incubated in 1% uranyl acetate in MB for 1hr. After incubation, the samples were washed once in MB, followed by two washes in water, and subsequently dehydrated in graded alcohols (10 minutes each; 50%, 70%, 90%, 2 X 100%). The samples were then incubated in propylene oxide for 1 hour and infiltrated overnight in a 1:1 mixture of propylene oxide and TAAB Epon (TAAB Laboratories Equipment

Ltd). The following day, the samples were embedded in TAAB Epon and polymerized at 60°C for 48 hours.

Ultrathin sections (~80nm) were cut using a Reichert Ultracut-S microtome, mounted onto copper grids, stained with lead citrate, and examined with a JEOL 1200EX transmission electron microscope. Nine fields were imaged for each cross-section at 2,000X magnification (33.9 μm x 22.2 μm). 10,000X mag images were also taken to show axon degeneration in detail. Images were recorded with an AMT 2k CCD camera and saved as TIFF files.

ERG/VEP recording

Recordings were performed using the Celeris system (Diagnosys) in a dark room. Mice at the desired age were dark-adapted overnight. The next day, mice were deeply anesthetized with intraperitoneal (IP) injections of 100-120mg/kg ketamine IP and 10 mg/kg xylazine. The anesthetized mouse was placed on the platform heated to 37.4°C, gently stretched to its full length, with its head positioned on a stand. Care was taken to ensure that the mouse's head remained still during breathing.

For flash ERG and flash VEP, a flash LED stimulator with a built-in Ag/AgCl electrode was placed in close contact with each eye of the animal, and eye gel was applied as a lubricant. A platinum recording electrode for VEP was inserted through the skin and placed subcutaneously along the midline above the visual cortex. A reference electrode for VEP was inserted into the snout, and a grounding electrode was placed subcutaneously near the tail. ERG and fVEP were recorded simultaneously, alternating between eyes, with one electrode serving as the recording electrode and the other as the reference electrode. The recording parameters were as follows: 1 $cd \cdot s/m^2$ luminance, 5-second intervals, 60 sweeps per run, a 20-ms baseline, a 0.125-300 Hz bandpass filter for ERG, and a 1-100 Hz bandpass filter for fVEP. The ERG a-wave marker was placed at the lowest peak between 10-40 ms, and the b-wave marker at highest peak between 25-85 ms. The fVEP N1 marker was placed at the lowest peak between 30-50 ms.

Pattern VEP was recorded only from the right eye of each animal. A pattern stimulator with a built-in electrode was placed on the right eye, while the other setups remained the same as for flash recording. The protocol parameters were as follows: horizontal bars at 0.0589 cycles per degree, 340 mm viewing distance, 100% contrast, a view angle of 61°H and 48°V, 2 reversals per sec, 400 ms recording time with a 50 ms pre-stimulus baseline, 450 sweeps per result, 2 results per run, 50 cd/m^2 luminance, and a 1-100 Hz bandpass filter for pVEP.

Optomotor reflex assay

The optomotor reflex (OMR) was assessed using the qOMR system (PhenoSys). The setup consisted of a virtual cylinder displaying vertical sine wave gratings, projected in two-dimensional coordinate space on computer monitors arranged in a quadrangle around the testing arena, which was enclosed in a soundproof box. A video camera was positioned directly above the animal to record its behavior. When a rotating grating perceptible to the mouse was projected onto the cylinder wall, the mouse tracked the grating with reflexive head movements aligned with the rotation. The OmrStudio software automatically scored whether animals tracked the cylinder. The OMR index was calculated as the number of frames with correct tracking divided by the number of frames in which the head moved in the opposite direction to the gratings.

For the visual acuity test, moving sine wave gratings were presented at a fixed contrast of 100% and a fixed speed of 12 deg/sec, with varying spatial frequencies (0.025, 0.05, 0.1, 0.15, 0.2, 0.25, 0.3, 0.35, 0.4, 0.45, 0.5, 0.6, and 0.9 cpd), with the direction of movement changing every 5 seconds. Four sets of stimuli were used, differing only in the order of the spatial frequencies. The animal’s tracking behavior was assessed for 60 seconds at each spatial frequency, with a homogeneous gray stimulus with the same mean luminance for 10 seconds between gratings. These short testing epochs minimized the likelihood of the mouse adapting to the stimulus and confirmed that each animal was capable of tracking when a salient stimulus

was present. Each mouse was tested by two of the four stimulus sets on day 1 and the remaining two sets on day 2. The average response to each spatial frequency across the four tests was calculated and plotted. The detection threshold was set at 30% of the maximal OMR index above 1. Visual acuity was determined as the intersection between the response curve with the threshold.

For contrast sensitivity testing, a single stimulus pattern was used: sine wave gratings fixed at 0.2 cpd and 12 deg/sec, with decreasing contrasts (1-0.1, in 0.1 steps). Similar to the visual acuity test, the animal's tracking behavior was assessed for 60 seconds while the moving gratings were displayed, followed by a homogeneous gray stimulus of the same mean luminance for 10 seconds. Each mouse was tested twice on day 1 and twice again on day 2. The average response to each contrast across the four tests was calculated and plotted. The detection threshold was set at 30% of the maximal OMR index above 1. Contrast sensitivity was then determined as the intersection between the response curve with the threshold.

Compound action potential recording

Animals

Mice were dark-adapted for 1.5 hours to overnight. Both males and females were used, with ages ranging from P606 to P658 for WT and *Opa1*$^{R290Q/+}$ mice, and from P580 to P653 for *OPA1*$^{+/+}$*; Sarm1*$^{-/-}$, *Opa1*$^{R290Q/+}$*; Sarm1*$^{-/+}$, and *Opa1*$^{R290Q/+}$*; Sarm1*$^{-/-}$ mice. We analyzed 5-9 retinas from 3-6 mice of each genotype. For initial development and validation of the CAP recording method, including the CAP's sensitivity to synaptic antagonists and tetrodotoxin, animals of various genotypes were used. No overt variation with circadian time, sex, or age was observed.

Electrophysiology

Following dark adaptation, mice were anesthetized with Avertin. Each retina and its attached optic nerve (severed at the optic chiasm) was then carefully dissected from associated tissues

in Ames' medium (United States Biological or Sigma-Aldrich, supplemented with $NaHCO_3$ and equilibrated with 95% O_2/5% CO_2). The vitreous humor was preserved, with only debris (e.g., retinal pigment epithelium) removed to minimize the chance of mechanically damaging the retina and RGC axons. The optic nerve's dural sheath was also preserved. The retina was placed on the untreated glass surface of the recording chamber, and was superfused continuously with Ames' medium (~5-8 ml/min, 23 °C). The tissue was visualized on an upright microscope using infrared transillumination (850- or 940-nm center wavelength and 30-nm width at half-maximum) and differential interference contrast optics.

The recording electrode was a fire-polished, glass capillary (PG10165-4, World Precision Instruments) containing an AgCl pellet. It was back-filled with Ames' medium from the recording chamber. A minimal length of the optic nerve (<500 μm) was drawn into the electrode to ensure mechanical stability. The nerve was sometimes repositioned in the electrode to optimize signal-to-noise, making the use of bath solution more practical than a dedicated electrode solution.

In the *OPA1; Sarm1* cohort, a new electrode design was used to enhance signal amplitude. Essentially, this entailed ensheathing the suction electrode (now made with 2-000-210 glass, Drummond Scientific) within a shell that could be filled with air. This shell was cut from a glass capillary (TW100-6, World Precision Instruments), fire-polished to size, and connected with the suction electrode at its base with epoxy. The signal electrode was an AgCl pellet inside a compartment continuous with the suction electrode. The ground and reference electrodes were combined in an AgCl pellet in the bath. This air electrode's larger dimensions required a longer segment of the optic nerve to be drawn in. This design increased the maximum response amplitude by 1.5- to 2-fold across genotypes.

All pharmacological agents were added to the bath. Tetrodotoxin was applied at 300 nM in Ames' medium. The synaptic antagonists were 3 mM kynurenate, 100 μM picrotoxin, 100 μM D,L-AP4, and 10 μM strychnine in Ames' medium (112,113).

A differential amplifier (A-M Systems Model 3000 with headstage) was used for recording.

The signal was filtered between 0.1 Hz and 10 kHz. Sampling always met or exceeded the Nyquist minimum.

Optical Stimulation

Light from a 75-W xenon arc lamp was filtered to deplete heat while selecting intensity and wavelength, then focused through a 4× objective to produce a spatially uniform disc, centered on the retina. An electromechanical shutter was used to control stimulus timing. Stimuli were measured at the site of the preparation using a calibrated radiometer and spectrometer. 410 nm light was used (10-nm bandpass), which is near the isosbestic point of the mouse visual pigments, and light intensity is expressed as photoisomerized rhodopsin molecules per rod per second (R*/rod/s), using an effective collecting area of 0.5 μm^2, a wavelength of peak sensitivity for rhodopsin of 500 nm, and Govardovskii's spectral template (114). We began at an intensity that is suprathreshold for rod-driven responses in many RGC types (1 R*/rod/s) (115) or below. We increased the intensity until reaching saturation or near-saturation of the response (~1.5×10^5 R*/rod/s). We set the inter-pulse interval to avoid cumulative desensitization (i.e., responses at a given intensity showed no progressive change; <0.75% rhodopsin bleached in any given retina). The biphasic ON peaks in response to higher light intensities may reflect ON RGC types whose action potentials exhibit different latencies due to variations in the speed of retinal processing and axonal propagation; it also may reflect response dynamics of individual RGCs. The undershoot may reflect a reduction in firing below the spontaneous rate in darkness, as would be expected from adaptational mechanisms in the retina.

Crude mitochondrial preparation

Mice were euthanized with CO_2 for 5 minutes. Whole brains were extracted and homogenized on ice using a Dounce homogenizer with 30 strokes in 5 mL of isolation buffer (250mM Sucrose, 20mM HEPES (pH7.4), 2mM EGTA in H_2O). The brain homogenates were then drawn into a syringe through an 18G needle and expelled through a 25G needle, repeated 15 times. The

homogenates were centrifuged at 2,000 g for 10 minutes at 4°C, and the nuclei and cell debris were discarded. The supernatant was transferred to a new tube and centrifuged again at 2,000 g for 10 minutes. The resulting supernatant (S1) was collected and centrifuged at 10,000 g for 10 minutes. The supernatant (S2), containing the cytosolic fraction, was harvested, and the pellet, representing the crude mitochondrial fraction, was resuspended in 1 mL of isolation buffer. The resuspended mitochondria were transferred to a 1.5 mL Eppendorf tube and centrifuged at 10,000 g for 10 minutes as a wash step. The resulting mitochondrial pellet was then resuspended in 1 mL of isolation buffer to prepare the crude mitochondrial fraction. 25 µL of the resuspended mitochondria or the cytosolic fraction was mixed with 25 µL of RIPA buffer containing protease inhibitors (Millipore Sigma) and incubated for 10 minutes on ice. The lysates were then diluted 1:5 with RIPA buffer and used for BCA analysis to determine protein concentration. The crude mitochondrial preparation was then diluted to 2 mg/mL and aliquoted into six tubes (200 µL each) for the Proteinase K protection assay. The remaining mitochondria were stored in -80°C. The cytosolic fraction was diluted to 1 mg/mL with RIPA buffer and 4X Laemmli buffer, boiled at 95°C for 5 minutes, aliquoted, and stored at -80°C for western blotting.

Proteinase K protection assay

Buffers and reagents

Hypotonic solution: 5 mM Sucrose, 5 mM HEPES (pH7.4), 1 mM EGTA in H_2O

Hypertonic solution: 750 mM KCl, 80 mM HEPES (pH7.4), 1 mM EGTA in H_2O

Proteinase K solution (PK): 30ug/mL proteinase K in isolation buffer

Triton X-100 and Proteinase K solution (Tx-PK): 1% (v/v) Triton X-100, 30 µg/mL proteinase K in isolation buffer

PMSF solution: 200 mM PMSF dissolved in isopropanol

Assay

The six tubes containing aliquoted mitochondria (200 µL each) were centrifuged at 16,000 g for

30 minutes at 4°C. The supernatant was discarded, and the mitochondrial pellets in the first three tubes were incubated with 300 µL of the following: 1) isolation buffer; 2) PK solution; or 3) Tx-PK solution, for one hour at room temperature. After incubation, 3 µL of 200 mM PMSF was added to each tube to terminate digestion, followed by the addition of 100 µL of 4X Laemmli buffer. The samples were then boiled at 95°C for 5 minutes, aliquoted, and stored at -80°C for western blotting. For the remaining three tubes, the mitochondrial pellets were resuspended in 200 µL of hypotonic solution and incubated on ice for 15 minutes to induce osmotic shock. Subsequently, 200 µL of hypertonic solution was added to re-establish isotonic conditions. The samples were then centrifuged at 3,000g for 15min at 4°C to obtain mitoplasts. The mitoplasts were then treated in the same manner as the first three samples.

Co-immunoprecipitation on crude mitochondria

Crude mitochondria was prepared from one mouse brain as mentioned above. After the wash step, the mitochondria pellet was resuspended and lysed in 1mL of digitonin lysis buffer (10 mM Tris/Cl pH 7.5, 50 mM NaCl, 0.5 mM EDTA, 1% digitonin, protease inhibitor cocktail 1:200) on ice for 30 minutes. Lysates were centrifuged at 13,200 rpm for 15min at 4°C. The supernatant was collected and separated in equal volumes in two tubes. 30 µL of lysates from each tube was set aside as the input fraction. 10 µL of 4× Laemmli buffer was added to the input fraction, followed by boiling at 95°C for 5 minutes. For co-immunoprecipitation, 10 µL of rat anti-SARM1 antibody (5 µg, BioLegend) or the corresponding rat igG1 control (5 µg, BioLegend) was added to the remaining lysates and incubated overnight at 4°C. The next day, 50 µL of magnetic protein A beads was added to the lysates and incubated for 4 hours at 4°C. Then the beads were washed three times with 1 mL of wash buffer (10 mM Tris/Cl pH 7.5, 150 mM NaCl, 0.1% digitonin, 0.5 mM EDTA, protease inhibitor cocktail 1:200) at 4°C. The samples were then eluted with 55 µL of 2× Laemmli buffer and boiled at 95°C for 5 minutes. The samples were stored at -80°C until western blot analysis.

Western blotting

Protein samples were run on SDS-PAGE gels in Tris-Glycine buffer (25 mM Tris, 192 mM glycine, 0.1% SDS). Proteins were transferred to 0.4 µm nitrocellulose membranes in transfer buffer (25 mM Tris, 192 mM glycine, 10% methanol) at 300 mA for 90 minutes in a refrigerator. The membranes were trimmed to the desired size and blocked with 5% (w/v) milk in TBST for 30 minutes. Following blocking, the membranes were incubated overnight at 4°C in TBST containing primary antibodies. The following day, the membranes were washed three times with TBST, 10 minutes each, followed by a one-hour incubation at room temperature in TBST containing fluorescent secondary antibodies (LiCor). After three additional 5-minute washes with TBST, the membranes were imaged using the LiCor Odyssey CLx imager.

Reverse transcription and qPCR

Fibroblasts were seeded in 24-well plates at a density of 60,000 cells per well. The next day, RNA was extracted from each well as separate samples using the RNeasy kit (Qiagen). RNA concentrations were measured using a Nanodrop. For cDNA synthesis, 800 ng of fibroblast RNAs was used with the iScript Reverse Transcription kit (Bio-Rad). Cortical neurons were dissected from mouse brains and seeded at 10,000 cells per well in 24-well plates. On DIV8, RNA was extracted, and 200 ng of RNA was used for cDNA synthesis. 5MO and 24MO mouse brains (20mg) were used for RNA extraction using the same RNeasy kit, and 1000ng of RNA was used for cDNA synthesis.

PCR of *Opa1* isoforms in fibroblasts and cortical neurons was performed using Q5 Hot Start DNA Polymerase, *Opa1*-specific primers (see Supplemental Table 2), and 2 µL of cDNA, with an annealing temperature of 67°C, a one-minute extension time, and 28 cycles. PCR of the *ActB* control was performed with the same cDNA templates, DNA Polymerase, and *ActB* specific primers, using a 67°C annealing temperature, a one-minute extension time and 32 cycles. The PCR products were resolved on a 1% agarose gel stained with EtBr.

RT-qPCR of *Sarm1* in brain samples was performed using the PowerUp™ SYBR™ Green Master Mix (Thermo Fisher Scientific). *Gapdh* was used as the control (primers listed in Supplemental Table 2). Relative expression was determined using the delta delta Ct method.

mtDNA content measurements

WT and *Opa1*$^{R290Q/+}$ MEFs were collected from flasks during passaging, and 1.5-2.5 million cells were used for DNA extraction using the DNeasy kit (Qiagen). DNA concentrations were measured with a Nanodrop and diluted to 40 ng/µL. qPCR was performed using the PowerUp™ SYBR™ Green Master Mix (Thermo Fisher Scientific), gene-specific primers (see Supplemental Table 1), and 120ng DNA templates. Triplicates were used for each gene and sample. C_T values were obtained for each sample, and fold changes were calculated using the $\Delta\Delta C_T$ method.

Metabolomics

Fibroblasts

<u>Metabolite extraction.</u> Fibroblasts were seeded in 15 cm dishes and cultured until 90% confluence. Prior to extraction, the cells were gently washed once with 5 mL of regular culture media and incubated with 10 mL of culture media for 2 hours. The media was then completely aspirated, and 4 mL of 80% methanol (-80°C) was immediately added. The dishes were then transferred to -80°C on dry ice and incubated for 15 minutes. After incubation, the cells were scraped with cell scrapers, and the cell lysate/methanol mixture was transferred to 15 mL Falcon tubes on dry ice. The lysates were centrifuged at full speed for minutes at 4°C to pellet cell debris and proteins. The supernatant was collected into 50 mL Falcon tubes on dry ice. 500 µL of 80% methanol (-80°C) was added to the 15 mL tube to resuspend the pellet. The mixture was transferred to1.5 mL Eppendorf tubes and centrifuged at full speed for 5 minutes at 4°C. The supernatant was transferred to the 50 mL Falcon tubes on dry ice containing the supernatants collected before. The extraction was repeated one more time and all three extractions were pooled in the 50 mL tube. The samples were then completely dried using a

speedVac for 5 hours.

Polar metabolomics profiling was performed at the Beth Israel Deaconess Mass Spectrometry Core as previously described (49).

Brains

Metabolite extraction. Mice were euthanized by CO_2 for 5 minutes, after which whole brains were immediately extracted, weighed, and flash-frozen in liquid nitrogen. Metabolites were extracted from bead-homogenized flash-frozen brains using 7 mL of 70% of 75ºC ethanol/water. Extraction was repeated once on the cell debris. The extraction solvent was spiked with 0.1% labelled amino acid mix (Cambridge Isotope Laboratories Inc., MSK-A2-1.2). The extracts were dried in a centrifugal vacuum unit, resuspended water and diluted in 80/20 acetonitrile/water with 1% formic acid to final volume of 400 µL for brain samples, and cleaned using a pass-through plate (Ostro Protein Precipitation & Phospholipid removal plate, Waters, SKU 186005518), dried in a centrifugal vacuum unit, and reconstituted in 80/20 acetonitrile/water.

LC/MS analysis. For metabolite analysis, a mass spectrometer (QExactive HF-X) was equipped with HESI II probe and coupled to a Vanquish binary UPLC system (Thermo Fisher Scientific, San Jose, CA). 5 µL of each metabolite extract was injected onto a BEH Z-HILIC column (100 mm, 1.7 µM particle size, 2.1 mm internal diameter, Waters). Mobile phase A was 15 mM ammonium bicarbonate in 90% water and 10% acetonitrile, and mobile phase B was 15 mM ammonium bicarbonate in 95% acetonitrile and 5% water. The column oven was held at 45°C and autosampler at 4°C. The chromatographic gradient was carried out at a flow rate of 0.5 mL/min as follows: 0.75 min initial hold at 95% B; 0.75-3.00 min linear gradient from 95% to 30% B, 1.00 min isocratic hold at 30% B. B was brought back to 95% over 0.50 minutes, after which the column was re-equilibrated under initial conditions. The analysis was performed in negative mode for each sample and gradient. The mass spectrometer was operated in full-scan mode (m/z = 70–1,050), with the spray voltage set to 3 kV, the capillary temperature to 320 °C, and the HESI probe to 300 °C. The sheath gas flow was 50U, aux gas 10U, and the sweep gas

was 1U, resolving power was 120,000. tSIM experiments were set up for GSH and GSSG using authentic standards. Water, formic acid, and acetonitrile were purchased from Fisher and were Optima LC/MS grade. Ammonium bicarbonate powder was purchased from Merck, and ethanol from Decon laboratories.

QUANTIFICATION AND STATISTICAL ANALYSIS

Mitochondrial length measurements

Mitochondrial length was measured in Fiji (ImageJ, NIH) (116). Maximum intensity projections were generated from Z stacks of mitochondrial images and analyzed with the Mitochondrial Network Analysis (MiNA) (117) plugin. Parameters were carefully adjusted to prevent over-fragmentation or over-fusion artifacts. The “Summed branch lengths mean” outputs were extracted and used for quantification.

RGC counts

Maximum projections of retinal stacks were generated using Fiji, and quantified in a blind manner. The total number of RGCs per retina was calculated as the average RGC count across the four quadrants. Dying RGCs were identified by phospho-H2Ax positive nuclei, and the percentage of dying RGCs was determined by dividing the average number of dying RGCs by the average total RGC number for that retina. Both the total number of RGCs and the percentage of dying RGCs were normalized to the WT means for each age group and displayed as box plots showing median, quantiles, minimum, maximum, and individual data points. This normalization facilitates a clearer comparison of the RGC degeneration patterns between WT and mutant mice across different ages, accounting for age-specific variations in WT RGC counts. With this normalization, different age groups were treated as independent datasets, and statistical analyses were performed using Mann-Whitney tests for comparisons within each age

group containing two genotypes, and one-way ANOVA with Tukey's multiple comparisons for each age group with three genotypes.

EM analyses

EM images were quantified using Fiji. Numbers of total axons and degenerating axons were counted and averaged across the nine fields of each optic nerve section. The percentage of degenerating axons was calculated by dividing the average number of degenerating axons by the average total number of axons for each optic nerve. These values were then normalized to the WT means for each age group, similar to the RGC counts, and presented as box plots. Mann-Whitney tests were performed within each age group containing two genotypes, while one-way ANOVA with Tukey's multiple comparisons was used for each age group with three genotypes.

ERG/VEP quantifications

ERG/VEP results were exported from the Celeris system as .csv files. The following values were extracted for each animal: a/b wave amplitudes of flash ERGs from both eyes, N1 amplitudes of flash VEPs from both eyes, and N1 amplitudes of pattern VEPs from the right eye. The a/b wave amplitudes of ERGs and N1 amplitudes of fVEPs were averaged between the two eyes for each animal. These values were then normalized to the WT means for each age group and presented as box plots. Mann-Whitney tests were performed within each age group containing two genotypes, while one-way ANOVA with Tukey's multiple comparisons was used for each age group with three genotypes. For the response traces in 18 MO animals, raw values were averaged across all animals and plotted as Mean ± SEM.

Metabolomics data analysis

Target metabolites and internal standards were analyzed using emzed (118). Peak areas were normalized by dividing each peak area by the measured biomass in each sample and

subsequently by the average of internal standard peak areas detected in the data file. Normalized peak areas were used for quantification.

CAP analyses

Responses from a retina at a given intensity were baseline-subtracted, averaged, and low-pass filtered (10 Hz) prior to analysis. A permutation test was used to estimate the likelihood that observed differences between samples arose by chance. Data values were resampled using a bootstrapping algorithm, randomly assigned to two groups, and compared using the Mann-Whitney U test. Repeating this operation 100,000 times yielded a distribution of U test statistics, to which the U test statistic of the actual datasets was compared. Effect sizes are given as Cohen's d. Responses with an average Z-score [(response peak - avg baseline)/ baseline SD] of <10 were not used for statistical analysis.

Immunoblot quantifications

Western blots were imaged on the Odyssey CLx imager. Protein intensities were quantified using the Image Studio Light software (LiCor). For the mitochondrial PK assay, signals for each protein were normalized to the untreated control. Results were presented as bar graphs. Statistical analyses were conducted using one-way ANOVA followed by Tukey's multiple comparisons.

Statistical analyses

Datapoints represent biological replicates. Box plots denote minimum, first quartile, median, third quartile, and maximum values. Statistical analyses were conducted using Prism (v10; GraphPad Software). Specific statistical analyses used are described in figure legends and methods. P-values are listed in graphs and Supplemental Table 1.

Resources table

REAGENT or RESOURCE	SOURCE	IDENTIFIER
Antibodies		
BD Transduction Laboratories™ Purified Mouse Anti-OPA1	BD Biosciences	Cat#612606; RRID: AB_399888
Chicken anti-MAP2 Antibody	Novus Biologicals	Cat#NB300-213; RRID: AB_2138178
Mouse anti-HA-Tag (6E2) mAb	Cell Signaling Technology	Cat#2367S; RRID: AB_10691311
Chicken anti-GFP antibody	Aves Labs	Cat#GFP-1020; RRID: AB_10000240
Guinea Pig anti-RBPMS Antibody	Novus Biologicals	Cat#NBP2-80389
Guinea Pig anti-Anti-RBPMS Antibody	PhosphoSolutions	Cat#1832-RBPMS; RRID: AB_2492226
Rabbit anti-Phospho-Histone H2A.X (Ser139) (20E3) mAb	Cell Signaling Technology	Cat#9718S; RRID: AB_2118009
Rabbit Living Colors® DsRed Polyclonal Antibody	Takara	Cat#632496; RRID: AB_10013483
Mouse anti-TOMM20 monoclonal antibody (M01), clone 4F3	Abnova	Cat#H00009804-M01; RRID: AB_519121
BD Pharmingen™ Purified Mouse Anti-Cytochrome C	BD Biosciences	Cat#556433; RRID: AB_396417
Rabbit anti-Tim23 Polyclonal antibody	Proteintech	Cat#1123-1-AP; RRID: AB_615045
Rabbit anti-HSP60 Antibody	Novus Biologicals	Cat#NBP1-77397; RRID: AB_11034890
Rabbit anti-Tubulin α antibody	Millipore Sigma	Cat#SAB4500087; RRID: AB_10743646
Mouse anti-SARM1 mAb	Yi-Ping Hsueh lab Chen et al. (81)	N/A
Goat Anti-Chicken IgY H&L (Alexa Fluor® 405)	Abcam	Cat#ab175674; RRID: AB_2890171
Goat anti-Mouse IgG (H+L) Cross-Adsorbed Secondary Antibody, Alexa Fluor™ 488	Invitrogen	Catalog#A-11001; RRID: AB_2534069
Alexa Fluor® 488-AffiniPure Donkey Anti-Guinea Pig IgG (H+L)	Jackson ImmunoResearch	Cat#706-545-148; RRID: AB_2340472
Goat anti-Rabbit IgG (H+L) Highly Cross-Adsorbed Secondary Antibody, Alexa Fluor™ 568	Invitrogen	Catalog#A-11036; RRID: AB_10563566
IRDye® 800CW Donkey anti-Mouse IgG Secondary Antibody	LiCor Biosciences	Cat#926-32212
IRDye® 680RD Donkey anti-Rabbit IgG Secondary Antibody	LiCor Biosciences	Cat#926-68073
Rat anti-SARM1 mAb	BioLegend	Cat#696602; RRID: AB_2687069
Purified Rat IgG1, κ Isotype Ctrl Antibody	BioLegend	Cat#400402; RRID: AB_326508
Bacterial and virus strains		
NEB® Stable Competent *E. coli*	NEB	Cat#C3040H
DH5α Competent Cells	Thermo Scientific	Cat#EC0112
Lentivirus: EF1a-MitoDsRed	This paper	N/A

Lentivirus: hSyn-MitoDsRed	This paper	N/A
Lentivirus: CMV-mouse SARM1-3×HA	This paper	N/A
Lentivirus: FCIV-DN SARM1-Venus	Geisler et al. (82)	N/A
Chemicals, peptides, and recombinant proteins		
DMEM, High Glucose, GlutaMAX	Life Technologies	Cat#10566-016
Fetal bovine serum (FBS)	R&D Systems	Cat#S11150H
Penicillin-Streptomycin	Sigma-Aldrich	Cat#P0781-20ML
$MgCl_2$	Sigma-Aldrich	Cat#M0250
Kynurenic acid	Sigma-Aldrich	Cat#K3375
HEPES	Sigma-Aldrich	Cat#H3375
HBSS	Gibco	Cat#14170161
BSA	Sigma-Aldrich	Cat#A3311
Papain	Worthington Biochemical	Cat#LS003126
DL-Cysteine hydrochloride	Sigma-Aldrich	Cat#C9768
Trypsin inhibitor	Worthington Biochemical	Cat#LS003086
Neurobasal™ Plus Medium	Gibco	Cat#A3582901
B-27™ Plus Supplement (50X)	Gibco	Cat#A3582801
Penicillin-Streptomycin-Glutamine (100X)	Gibco	Cat#10378016
Poly-L-lysine hydrobromide	Sigma-Aldrich	Cat#P2636-25MG
Laminin Mouse Protein, Natural	Gibco	Cat#23017015
DMEM without phenol red for lentiviral packaging	Gibco	Cat#31053036
PEI MAX®	Polysciences	Cat#24765
F68 Poloxamer 188 Non-ionic Surfactant (100X)	Gibco	Cat#24040032
EcoNI	NEB	Cat#R0521L
Opti-MEM™ I Reduced Serum Medium	Gibco	Cat#31985-062
Triton X-100	Sigma-Aldrich	Cat#T8787
Superblock	Thermo Scientific	Cat#PI37515
VECTASHIELD® Antifade Mounting Medium	Vector Laboratories	Cat#H-1000-10
Proteinase K, recombinant, PCR grade	Thermo Scientific	Cat#EO0491
Sodium acrylate	Fisher Scientific	Cat#NC2403321
40% acrylamide solution	Bio-Rad	Cat#1610140
N, N'-Methylenebisacrylamide	Sigma-Aldrich	Cat#M7279
Sodium chloride	VWR	Cat#X190-5KG
TEMED	Bio-Rad	Cat#1610800
Ammonium Persulfate (APS)	Bio-Rad	Cat#1610700
Guanidine hydrochloride solution	Sigma-Aldrich	Cat#G7294
Form/Glut 2.5% in 0.1M Sodium Cacodylate Buffer pH7.4	EMS	Cat#15949
Normal Donkey Serum	Jackson ImmunoResearch	Cat#017-000-121; RRID: AB_2337258
Invitrogen™ DAPI and Hoechst Nucleic Acid Stains	Invitrogen	Cat#PI62249
RIPA Buffer, Liquid, pH 7.4	Boston BioProducts	Cat#BP-115
cOmplete™ EDTA-free Protease Inhibitor Cocktail	Millipore Sigma	Cat#11836170001
TWEEN® 20	Sigma-Aldrich	Cat#9416
Glucose	Gibco	Cat#A2494001
Sodium pyruvate	Gibco	Cat#11360070

Oligomycin	Enzo Life Sciences	Cat#ALX-380-037-M010
FCCP	Tocris Bioscience	Cat#0453
Antimycin A from Streptomyces sp.	Sigma-Aldrich	Cat#A8674
Rotenone	Tocris Bioscience	Cat#3616
Seahorse XF DMEM Medium pH 7.4	Agilent	Cat#103575-100
Ames Medium w/L-Glutamine	United States Biological Corporation	Cat#A1372-25
Ames' Medium	Sigma-Aldrich	Cat#A1420
Tetrodotoxin	Alomone Labs	Cat#T-550
Kynurenate	Sigma-Aldrich	Cat#K3375-5G
Picrotoxin	Tocris	Cat#1128
D,L-AP4	Tocris	Cat#0103
Strychnine	Sigma-Aldrich	Cat#S0532-100G
OptiPrep - Density Gradient Media (Iodixanol)	Cosmo Bio USA	Cat#AXS-1114542-5
Metabolomics Amino Acid Mix Standard	Cambridge Isotope Laboratories	Cat#MSK-A2-1.2
Digitonin	Sigma-Aldrich	Cat#D141
Protease Inhibitor Cocktail Set III, EDTA-Free	Millipore Sigma	Cat#539134-1ML
Dynabeads™ Protein A for Immunoprecipitation	Invitrogen	Cat#10001D
EGTA (1 M, pH 7.4)	Boston BioProducts	Cat#BM-723
Carbonyl Cyanide m-Chlorophenylhydrazone (CCCP)	Sigma-Aldrich	Cat#C2759
DMSO	Sigma-Aldrich	Cat#D2650
Critical commercial assays		
Pierce™ BCA Protein Assay Kits	Thermo Scientific	Cat#A55864
Q5® Site-Directed Mutagenesis Kit	NEB	Cat#E0554S
Q5® Hot Start High-Fidelity 2X Master Mix	NEB	Cat#M0494L
Gibson Assembly® Master Mix	NEB	Cat#E2611L
QIAprep Spin Miniprep Kit	Qiagen	Cat#27106
HiSpeed Plasmid Maxi Kit	Qiagen	Cat#12663
QIAquick Gel Extraction Kit	Qiagen	Cat#28706
QIAquick PCR Purification Kit	Qiagen	Cat#28106
RNeasy Mini Kit	Qiagen	Cat#74104
DNeasy Blood & Tissue Kit	Qiagen	Cat#69504
iScript™ Reverse Transcription Supermix	Bio-Rad	Cat#1708841
PowerUp™ SYBR™ Green Master Mix for qPCR	Thermo Fisher Scientific	Cat#A25741
TransIT-LT1 Transfection Reagent	Mirus Bio	Cat#MIR 2300
Seahorse FluxPaks	Agilent	Cat#103793-100
Experimental models: Cell lines		
Human: HEK293T	ATCC	Cat#CRL-11268
WT and *Opa1*$^{R290Q/+}$ embryonic fibroblasts	This paper	N/A
WT and *Opa1*$^{R290Q/+}$ cortical neurons	This paper	N/A
Experimental models: Organisms/strains		

Mouse: C57BL/6J	The Jackson Lab	RRID: IMSR_JAX:000664
Mouse: *Opa1*$^{R290Q/+}$	This paper	N/A
Mouse: B6.129X1-*Sarm1*^{tm1Aidi}/J	The Jackson Lab	RRID: IMSR_JAX:018069
Oligonucleotides		
Primers, the OPA1 guide RNA and repair template are included in Supplemental Tables	This paper	N/A
Recombinant DNA		
pEGFP-N1-OPA1 iso1 WT	This paper	N/A
pEGFP-N1-OPA1 iso1 R290Q	This paper	N/A
pLVX-EF1a-MitoDsRed2	Addgene	Cat#174541
pLVX-CMV-mouse SARM1-3×HA	This paper	N/A
FCIV-DN SARM1-Venus	Geisler et al. (82)	N/A
psPax2	Addgene	Cat#12260
pMD2.G	Addgene	Cat#12259
Software and algorithms		
Fiji (ImageJ)	Schindelin et al. (116)	https://imagej.net/software/fiji/
PRISM	Garphpad Software	https://www.graphpad.com/
Biorender	N/A	https://www.biorender.com/
Image Studio Lite	LICORbio	https://www.licor.com/bio/image-studio/
Python	Python Software Foundation	https://www.python.org
Igor Pro	WaveMetrics	https://www.wavemetrics.com/
RELION5	Burt el al. (104)	https://relion.readthedocs.io/en/release-5.0/
AreTomo	Zheng et al. (106)	https://drive.google.com/drive/folders/1Z7pKVEdgMoNaUmd_cOFhlt-QCcfcwF3_
CTFFIND4	Rohou et al. (105)	https://grigoriefflab.umassmed.edu/ctffind4
AMIRA	Thermo Fisher Scientific	https://www.thermofisher.com/us/en/home/electron-microscopy/products/software-em-3d-vis/amira-software/cell-biology.html

Surface morphometrics	Barad et al. (57)	https://github.com/GrotjahnLab/surface_morphometrics
Membrain-seg	Lamm et al. (111)	https://github.com/teamtomo/membrain-seg
cryoCARE	Buchholz et al. (108) and Buchholz et al. (109)	https://github.com/juglab/cryoCARE_pip
isoNET	Liu et al. (107)	https://github.com/IsoNet-cryoET/IsoNet
AutoTEM	Thermo Fisher Scientific	https://www.thermofisher.com/us/en/home/electron-microscopy/products/software-em-3d-vis/autotem-5-software.html
EPU TOMO	Thermo Fisher Scientific	https://www.thermofisher.com/us/en/home/electron-microscopy/products/software-em-3d-vis/tomography-software.html
Dynamo	Castano-Diez et al. (110)	https://www.dynamo-em.org/w/index.php?title=Installation
Other		
GG-12-1.5-Pre, 12mm dia.#1.5 thick 90 pc Pre-treated German coverslips	neuVitro	Cat#GG-12-15-Pre
Fisherbrand™ Disposable PES Filter Units	Fisherscientific	Cat#FB12566503
24 Well glass bottom plate with high performance #1.5 cover glass	Cellvis	Cat#P24-1.5H-N
35 mm Glass bottom dish with 20 mm micro-well #1.5 cover glass	Cellvis	Cat#D35-20-1.5-N
Round German Coverslip 25mm #1	Bellco Glass, Inc	Cat#1943-10025A
Celeris ERG/VEP system	Diagnosys	Cat#D430
Patch Clamp Glass Capillaries	World Precision Instruments	Cat#PG10165-4
Drummond Scientific Calibrated Micropipets	Drummond Scientific	Cat#2-000-210
Thin Wall Glass Capillaries	World Precision Instruments	Cat#TW100-6
Ostro Protein Precipitation & Phospholipid Removal Plate	Waters	Cat#186005518

Supplemental Table 1
guide RNA and repair template for OPA1 R290Q CRISPR

Opa1 R290Q guide RNA	Sequence 5'-3'
Opa1 -R290Q-crRNA	AAGATCACCTACCACGGGTA
PAM	AGG
gRNA location	chr16:29607753-29607775
Opa 1 R290Q HDR template	Sequence 5'-3'
Opa1 -R290Q-HDR	ttcttccaaaacatatatatttgcttttatggtctccaaaataaggaacgaa<br>gcaatgctttgtatctgaggtatcatgggaaatacagaaattactatgaca<br>caaggaaagaactttcctgctgacagcaggtcctggtctattttccccttac<br>CtGTGGTAGGTGATCTTGTGTATTGTAACTGGCATCATA<br>ATCAGAAAGAACATCAAGAACTTCAGAATACATATCAAT<br>CAAAGATTTctgcaaaaaaattaaaattagcaaaagtcagtgaaaat<br>aaaacaaagaccacagatacatgaagagaaaatgtagatacattcaaat<br>ttgcattg
Genomic PCR screening primers	Sequence 5'-3'
Opa1 screen-F	GTTGGTCACCTGACAGTGTATTTG
Opa1 screen-R	GGTCTCCAAAATAAGGAACGAAGC
Genomic PCR sequencing primers	Sequence 5'-3'
Opa1 seq-F	CTCTTCATGTATCTGTGGTC
Opa1 seq-R	GAACGAAGCAATGCTTTG
Validation PCR and sequencing	Sequence 5'-3'
Opa1 screen-F	GTTGGTCACCTGACAGTGTATTTG
Opa1 screen-R2	TGATCTCCAACCACAACAACCT

Supplemental Table 2
List of PCR primers

Opa1 genotyping primers	Sequence 5'-3'
Opa1 screen-F	GTTGGTCACCTGACAGTGTATTTG
Opa1 screen-R2	TGATCTCCAACCACAACAACCT

Sarm1 genotyping primers	Sequence 5'-3'
Common	GAAATGCATGGAGGGGTTG
Wild type Reverse	CCACCAAACGTGTCCAATC
Mutant Reverse	TGTGGTTTCCAAATGTGTCAG

Primers for *Opa1* isoform abundanc	Sequence 5'-3'
Opa1 -exon4-Fwd	AGCTCAGAAGACCTTGCCAGTTTAG
Opa1 -exon9-Rev	CCTGAGCAATCATTTCCAGCAC

Primers for *ActB*	Sequence 5'-3'
Mouse *ActB* -Fwd	GGCTGTATTCCCCTCCATCG
Mouse *ActB* -Rev	CCAGTTGGTAACAATGCCATGT

Primers for *Sarm1* RT-qPCR	Sequence 5'-3'
Sarm1 -RT-PCR-Fw	TCGCAATTTTGTCCTGGTG
Sarm1 -RT-PCR-Rv	AGCTTAAAGCAGTCACAATCTCC

Primers for *Gapdh* RT-qPCR	Sequence 5'-3'
Gapdh -qPCR-Fw	TGACCTCAACTACATGGTCTACA
Gapdh -qPCR-Rv	CTTCCCATTCTCGGCCTTG

mtDNA and nDNA primers	Sequence 5'-3'
Mttl1 -Fwd	CGAAAGGACAAGAGAAATAGAG
Mttl1 -Rev	GAACAAGGTTTTAAGTCTTACGCA
16s rRNA-Fwd	CCGCAAGGGAAAGATGAAAGAC
16s rRNA-Rev	TCGTTTGGTTTCGGGGTTTC
Nd1 -Fwd	CTAGCAGAAACAAACCGGGC
Nd1-Rev	CCGGCTGCGTATTCTACGTT
Gapdh -Fwd	TGACCTCAACTACATGGTCTACA
Gapdh -Rev	CTTCCCATTCTCGGCCTTG

References

102. Aida T, et al. Cloning-free CRISPR/Cas system facilitates functional cassette knock-in in mice. *Genome Biol*. 2015;16(1):87.

103. Hagen WJH, et al. Implementation of a cryo-electron tomography tilt-scheme optimized for high resolution subtomogram averaging. *J Struct Biol*. 2017;197(2):191-198.

104. Burt A, et al. An image processing pipeline for electron cryo-tomography in RELION-5. *FEBS Open Bio*. 2024;

105. Rohou A, Grigorieff N. CTFFIND4: Fast and accurate defocus estimation from electron micrographs. *J Struct Biol*. 2015;192(2):216-21.

106. Zheng S, et al. AreTomo: An integrated software package for automated marker-free, motion-corrected cryo-electron tomographic alignment and reconstruction. *J Struct Biol X*. 2022;6:100068.

107. Liu YT, et al. Isotropic reconstruction for electron tomography with deep learning. *Nat Commun*. 2022;13(1):6482.

108. Buchholz TO, et al. Cryo-CARE: Content-Aware Image Restoration for Cryo-Transmission Electron Microscopy Data. 2019:502-506.

109. Buchholz TO, et al. Content-aware image restoration for electron microscopy. *Methods Cell Biol*. 2019;152:277-289.

110. Castano-Diez D, et al. Dynamo: a flexible, user-friendly development tool for subtomogram averaging of cryo-EM data in high-performance computing environments. *J Struct Biol*. 2012;178(2):139-51.

111. Lamm L, et al. MemBrain: A deep learning-aided pipeline for detection of membrane proteins in Cryo-electron tomograms. *Comput Methods Programs Biomed*. 2022;224:106990.

112. Liu A, et al. Encoding of environmental illumination by primate melanopsin neurons. *Science*. 2023;379(6630):376-381.

113. Do MT, et al. Photon capture and signalling by melanopsin retinal ganglion cells. *Nature*. 2009;457(7227):281-7.

114. Govardovskii VI, et al. In search of the visual pigment template. *Vis Neurosci*. 2000;17(4):509-28.

115. Smeds L, et al. Paradoxical Rules of Spike Train Decoding Revealed at the Sensitivity Limit of Vision. *Neuron*. 2019;104(3):576-587 e11.

116. Schindelin J, et al. Fiji: an open-source platform for biological-image analysis. *Nat Methods*. 2012;9(7):676-82.

117. Valente AJ, et al. A simple ImageJ macro tool for analyzing mitochondrial network morphology in mammalian cell culture. *Acta Histochem*. 2017;119(3):315-326.

118. Kiefer P, et al. eMZed: an open source framework in Python for rapid and interactive development of LC/MS data analysis workflows. *Bioinformatics*. 2013;29(7):963-4.

119. Do MTH. Melanopsin and the Intrinsically Photosensitive Retinal Ganglion Cells: Biophysics to Behavior. *Neuron*. 2019;104(2):205-226.

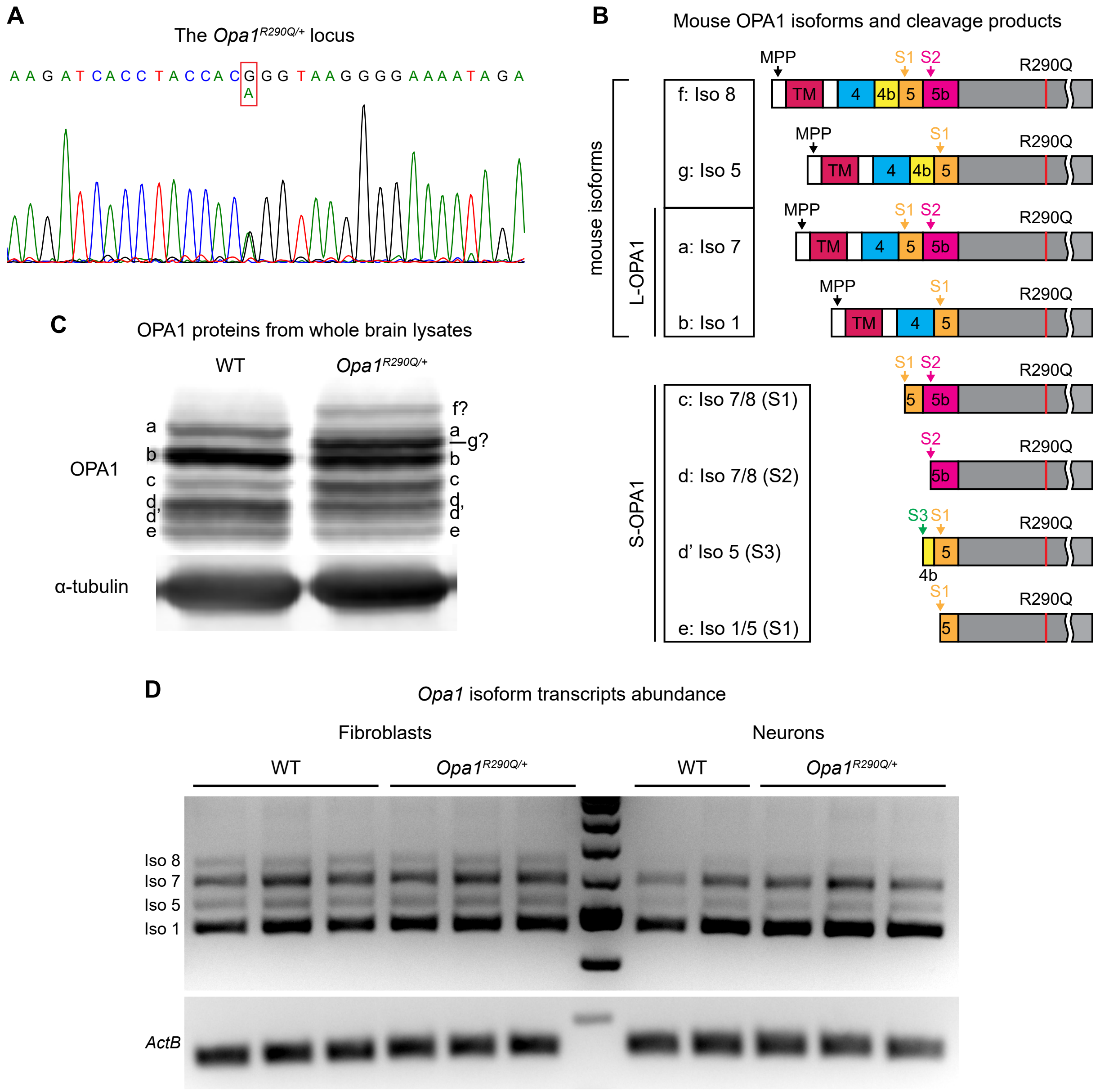

Supplemental Figure 1 - related to Figure 1. Impact of the R290Q point mutation on *Opa1* isoform abundance and post-translational processing
(**A**) The mouse *Opa1*$^{R290Q/+}$ locus and a representative Sanger sequencing result from PCR amplification of genomic DNA.
(**B**) Schematic representation of mouse OPA1 protein isoforms based on previous studies (22). The mouse expresses four *Opa1* isoforms. In WT cells, isoforms 5 and 8 are fully cleaved by OMA1 at site S1 and YME1L at site S2, while isoforms 1 and 7 undergo partial cleavage. The R290Q mutation falls within the common region present in all isoforms. MPP, mitochondrial processing peptidase; S1, OMA1 cleavage site; S2, YME1L cleavage site; S3, another YME1L cleavage site.
(**C**) Western blot analysis of OPA1 in brain lysates from post-natal day 0 WT and *Opa1*$^{R290Q/+}$ mice. In WT, the OPA1 pattern aligns with previous reports (46). In *Opa1*$^{R290Q/+}$ mice, two additional longer bands, likely corresponding to full-length isoforms 5 and 8, were observed. The intensity of other bands also changed, though the abundance of total OPA1 proteins remained similar.
(**D**) PCR analysis of *Opa1* isoform abundance in cDNAs extracted from fibroblasts (n = 3 WT and 3 *Opa1*$^{R290Q/+}$ passages) and neuronal cultures (n = 2 WT and 3 *Opa1*$^{R290Q/+}$ brains). *ActB* was used as the control. 4 splicing variants arose via the inclusion or exclusion of exons 4b and 5b and no appreciable changes due to R290Q were detected.

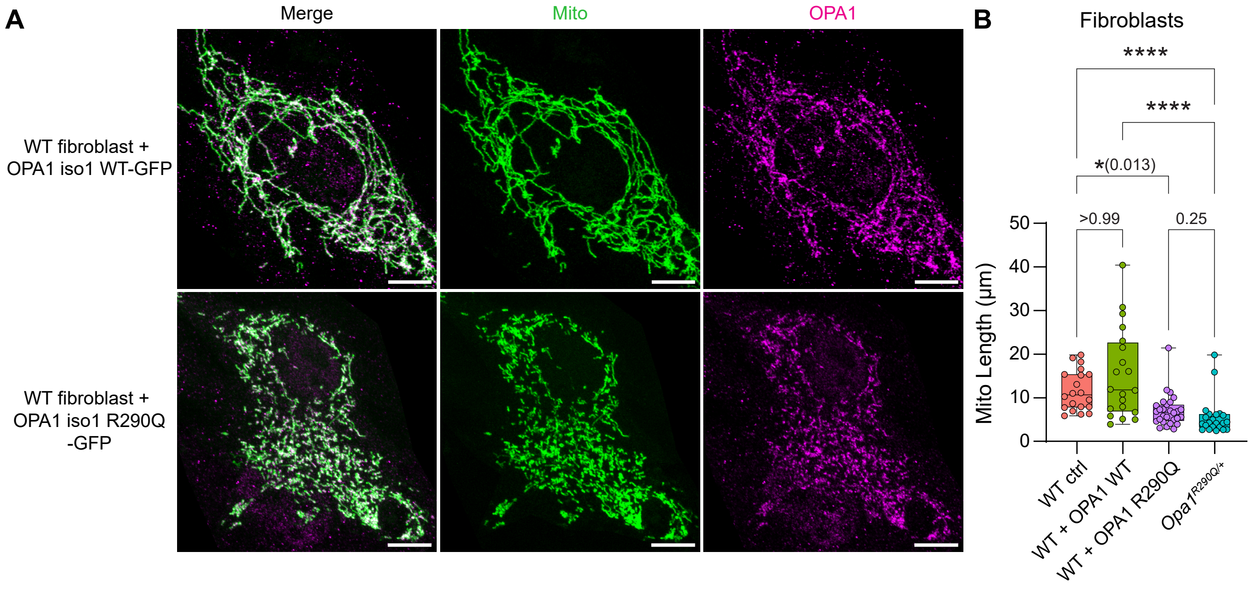

Supplemental Figure 2 - related to Figure 1. The OPA1 R290Q transgene causes mitochondrial fragmentation upon overexpression

(**A**) Representative images of WT fibroblasts expressing transgenes encoding either OPA1 isoform 1 WT-GFP or OPA1 isoform1 R290Q-GFP. Mitochondria (green) were labeled by expression of MitoDsRed and OPA1 (magenta) was labeled with GFP staining. Scale bars = 10 µm.

(**B**) Quantification of mitochondrial length in fibroblasts overexpressing OPA1 as in (**A**). Overexpression of OPA1 R290Q, but not WT OPA1, in WT fibroblasts resulted in mitochondrial fragmentation comparable to that observed in heterozygous fibroblasts. N = 20, 20, 28, and 22 cells for each condition, respectively, from one experiment. Kruskal-Wallis test with Dunn's multiple comparisons.

Box plots denote minimum, first quartile, median, third quartile, and maximum values. *$P < 0.05$; ****$P < 0.0001$.

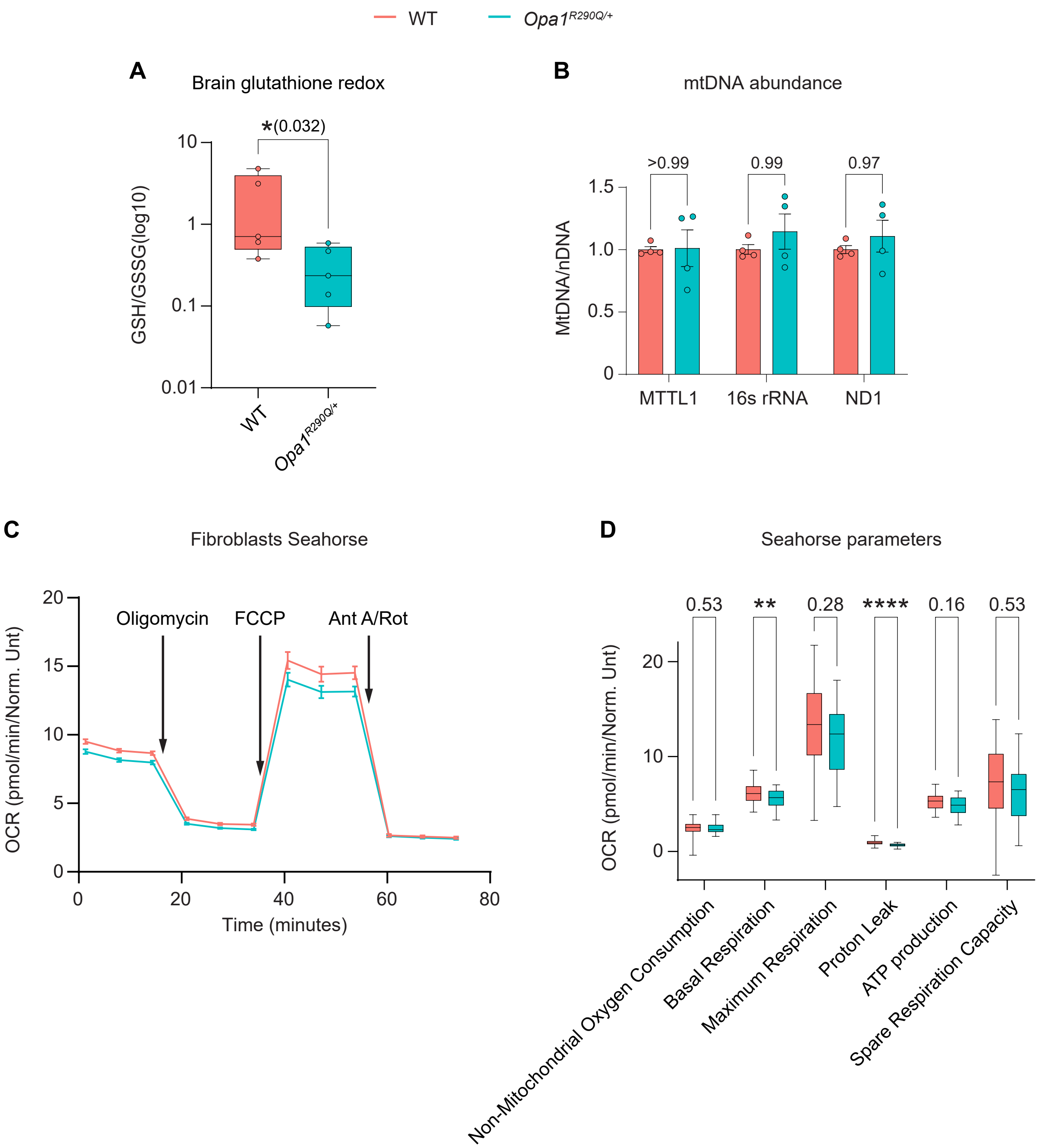

Supplemental Figure 3 - related to Figure 1. The *Opa1*$^{R290Q/+}$ mutation causes oxidative stress but has minimal impact on oxygen consumption and mitochondrial DNA stability in fibroblasts

(**A**) GSH/GSSG ratios in whole brain tissues as measured by LC-MC, as also shown in fibroblasts (Figure 1F). N = 5 WT brains and 5 *Opa1*$^{R290Q/+}$ brains from one experiment. Mann-Whitney test.

(**B**) Mitochondrial DNA abundance was measured by qPCR in DNA extracted from fibroblasts. GAPDH DNA was used as the nuclear DNA reference for normalization. N = 4 WT and 4 *Opa1*$^{R290Q/+}$ samples from different passages. Mean ± SEM. Multiple Mann-Whitney tests with Holm-Sidak multiple comparisons.

(**C**) Oxygen consumption rates of fibroblasts, as measured by the Seahorse assay. N = 62 WT wells and 58 *Opa1*$^{R290Q/+}$ wells from 4 experiments. Mean ± SEM.

(**D**) Parameters of mitochondrial function derived from the measurements in (**C**). Multiple unpaired t tests with Holm-Sidak multiple comparisons.

Box plots denote minimum, first quartile, median, third quartile, and maximum values. **P* < 0.05; ***P* < 0.01; *****P* < 0.0001.

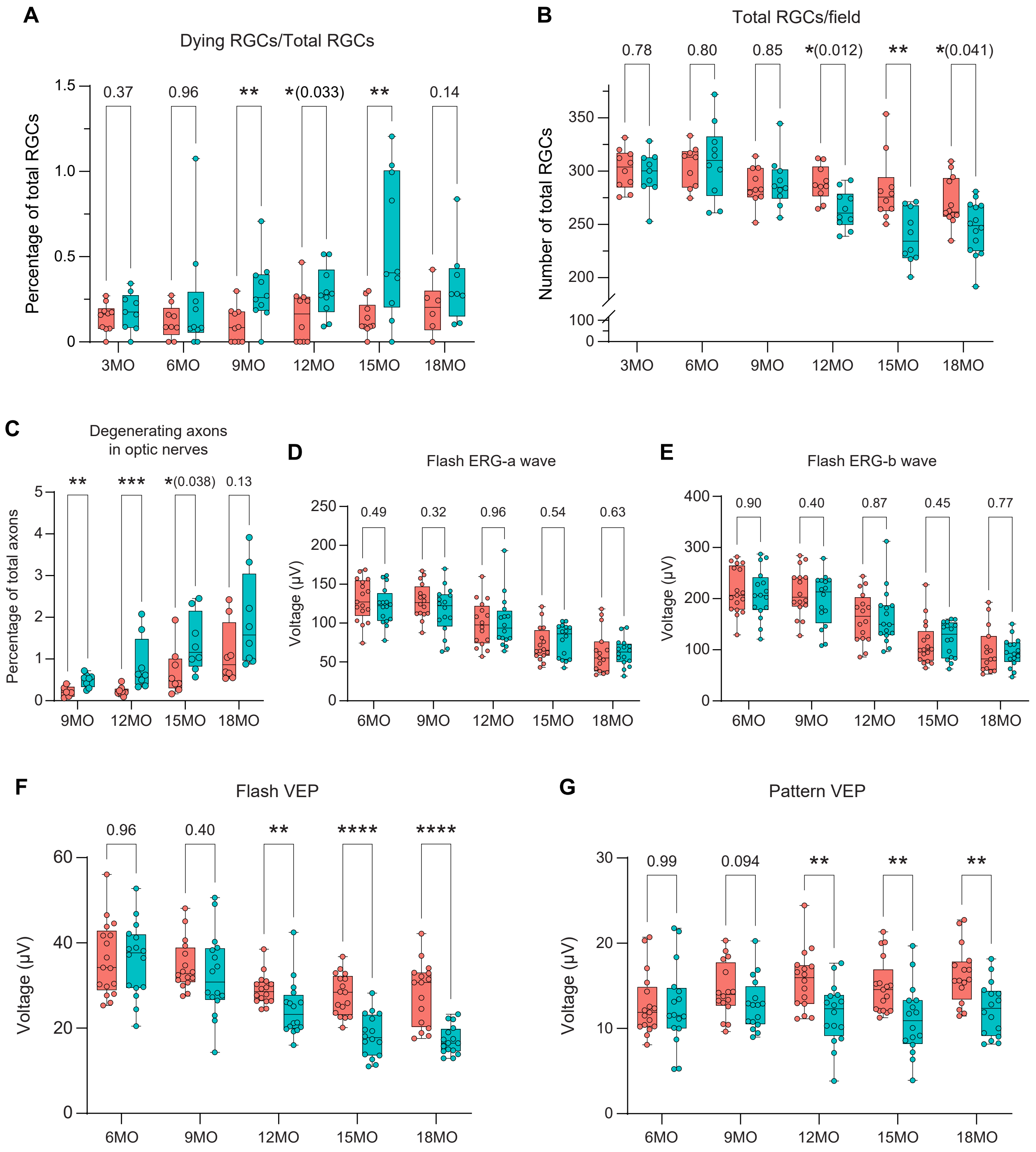

Supplemental Figure 4 - related to Figures 2 and 3. Quantifications of RGC degeneration and electrophysiology without normalization to wildtype values.

(**A**) Quantification of Dying RGCs over time. Each dot represents one retina. The counts of dying RGCs were averaged across the four quadrants of each retina and divided by the average RGC number of those quadrants. N = 9-12 WT and 9-14 *Opa1*$^{R290Q/+}$ retinas per age group.

(**B**) Quantification of total RGCs over time. Each dot represents one retina. RGC counts were averaged across the four quadrants of each retina. N = 6-10 WT and 8-10 *Opa1*$^{R290Q/+}$ retinas per age group.

(**C**) Quantification of degenerating axons by EM. Each dot represents one optic nerve. The counts of degenerating axons were averaged across the nine fields of each cross-section of an optic nerve and divided by the average axon number of those fields. N = 6-8 WT and 8 *Opa1*$^{R290Q/+}$ retinas per age group. An increase in axon degeneration with age was observed in WT controls, but the degeneration was greater in *Opa1*$^{R290Q/+}$ nerves.

(**D** and **E**) Amplitudes of a-wave (**D**) and b-wave (**E**) of flash ERG across age. Each dot represents one animal. N = 15-17 WT and 16 *Opa1*$^{R290Q/+}$ mice per age group. A similar age-dependent decrease was observed in both genotypes, likely due to progressively worsening electrode-cornea contact.

(**F** and **G**) N1 amplitudes of flash VEP (**F**) and pattern VEP (**G**) across age. Each dot represents one animal. N = 16-17 WT and 16 *Opa1*$^{R290Q/+}$ mice per age group. A slight increase in pVEP amplitudes with age was observed in WT controls but was absent in *Opa1*$^{R290Q/+}$ mice.

Box plots denote minimum, first quartile, median, third quartile, and maximum values. **P* < 0.05; ***P* < 0.01; ****P* < 0.001; *****P* < 0.0001 determined by Mann-Whitney test for each age group.

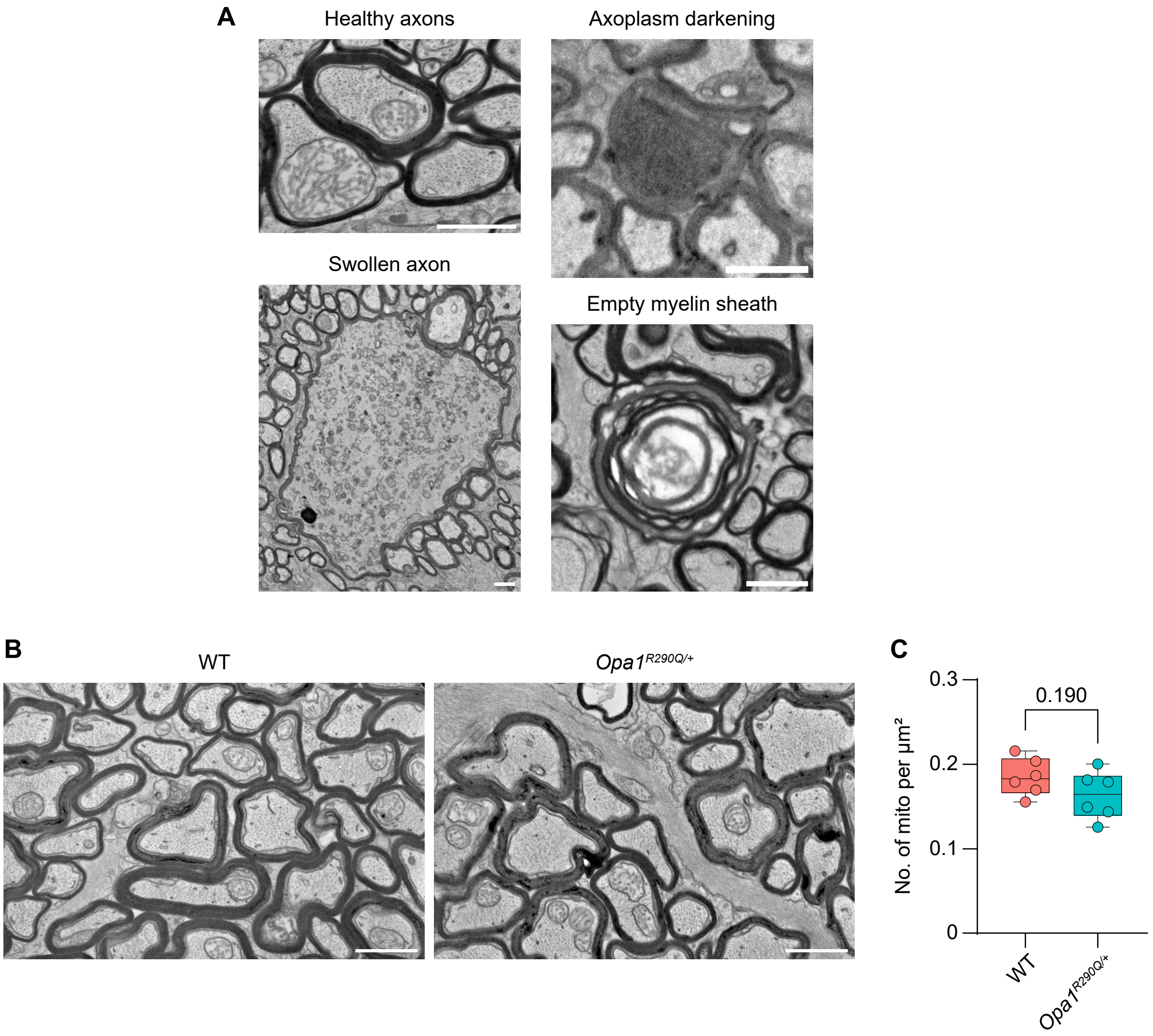

Supplemental Figure 5 - related to Figure 2. Degenerating axons and mitochondria in EM images of optic nerves
(**A**) Examples of healthy and degenerating axons. Healthy axons have compact myelin sheaths and microtubules in the axoplasm. Degenerating axons were classified into three categories: swollen axons with accumulation of organelles or neurofilaments, dark axons in the late stage of degeneration, and empty myelin sheath with completely degenerated axons. Scale bars = 1 µm.
(**B**) Mitochondria in optic nerves of 12MO animals. Scale bars = 1 µm.
(**C**) Mitochondrial density in optic nerves of 12MO animals. N = 6 WT and 6 *Opa1*$^{R290Q/+}$ optic nerves. Mann-Whitney test. Box plots denote minimum, first quartile, median, third quartile, and maximum values.

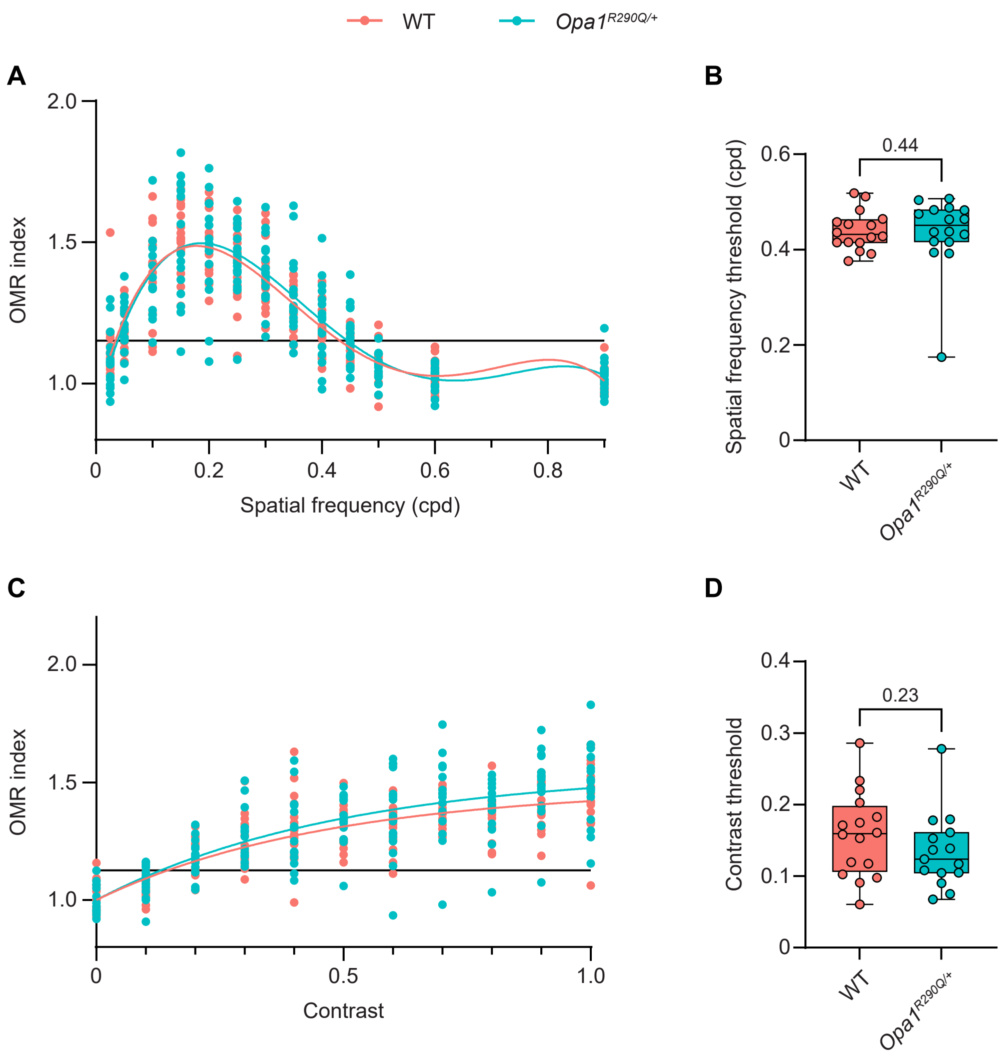

Supplemental Figure 6 - related to Figure 3. *Opa1*$^{R290Q/+}$ mice do not show significant decrease in the optomotor reflex (OMR)
(**A**) OMR index plotted as a function of spatial frequency (cycles per degree). The data was fitted with a 4th degree polynomial function. The horizontal line represents the OMR response threshold, set at 30% of the maximal response above a baseline of 1.
(**B**) Spatial frequency thresholds determined by the second intersection of the horizontal line in (**A**) with the fitted curve for each animal. N = 16 WT and 16 *Opa1*$^{R290Q/+}$ mice. Mann-Whitney test.
(**C**) OMR index plotted as a function of contrast (0 to 1). The data was fitted with a one phase association function. The horizontal line represents the OMR response threshold, set at 30% of the maximal response above a baseline of 1.
(**D**) Contrast thresholds determined by the intersection of the horizontal line in (**C**) with the fitted curve for each animal. N = 16 WT and 15 *Opa1*$^{R290Q/+}$ mice. Mann-Whitney test.
Box plots denote minimum, first quartile, median, third quartile, and maximum values.

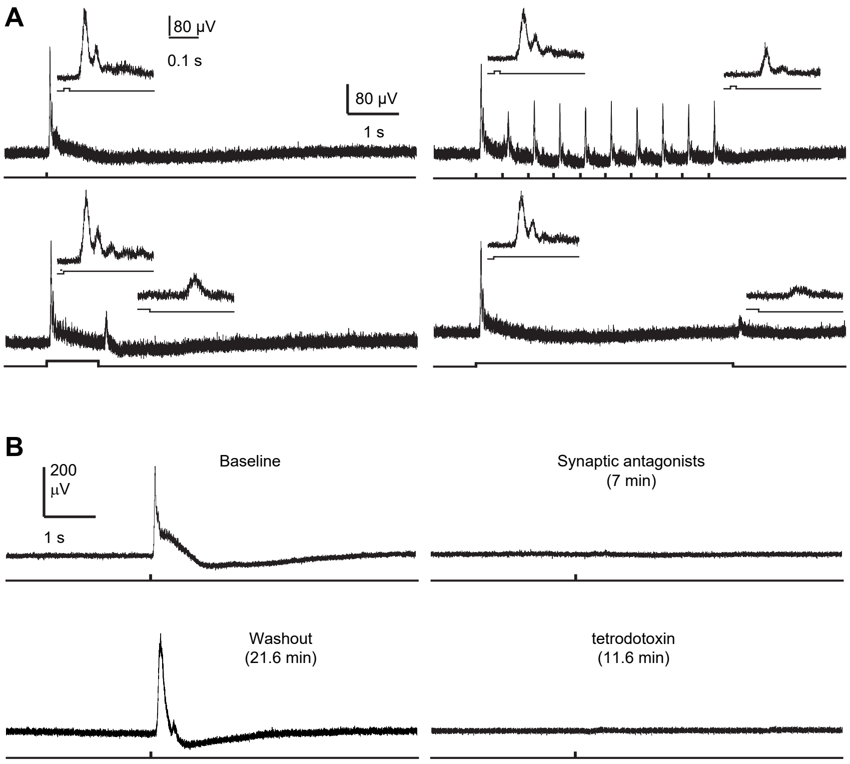

Supplemental Figure 7 - related to Figure 4. Basic features of the compound action potential
(**A**) Responses to flashes and steps of light.
(**B**) Block by pharmacological antagonists of synaptic transmission (top, 3 mM kynurenate, 100 µM picrotoxin, 100 µM D,L-AP4, and 10 µM strychnine) and action potentials (bottom). These effects were reversible (not shown). The intrinsic photosensitivity of melanopsin-expressing RGCs (119) was not evident, perhaps due to the low abundance of these neurons. Stimulus monitors are shown at the bottom.

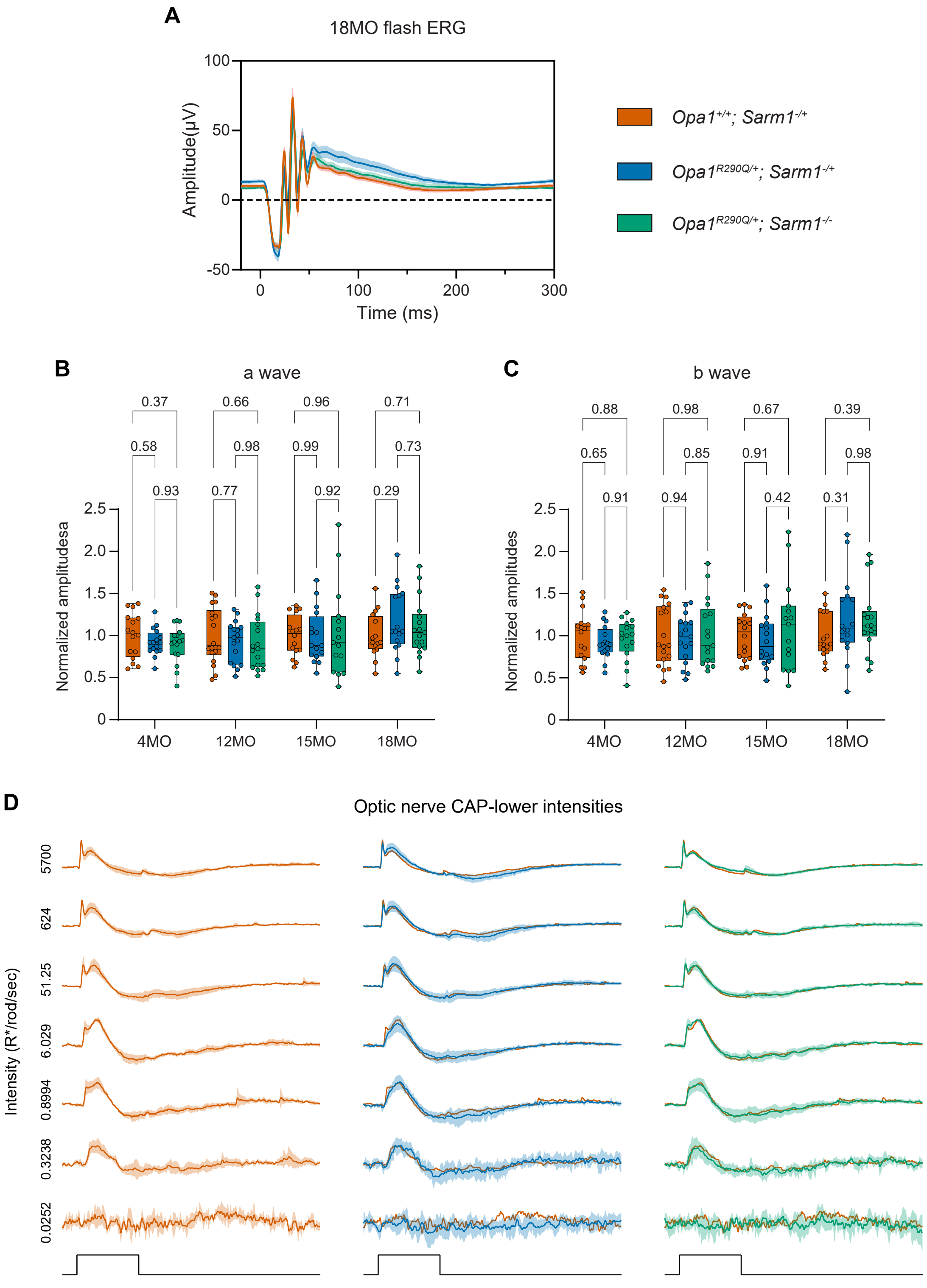

Supplemental Figure 8 - related to Figure 6. Flash ERG responses are not appreciably impacted by either the *OPA1$^{R290Q/+}$* mutation or *Sarm1* KO

(**A**) Average flash ERG traces in 18MO animals of the indicated genotypes. N = 16 *Opa1$^{+/+}$; Sarm1$^{-/+}$*, 16 *Opa1$^{R290Q/+}$; Sarm1$^{-/+}$*, and 17 *Opa1$^{R290Q/+}$; Sarm1$^{-/-}$* mice. Mean ± SEM.

(**B** and **C**) Amplitudes of a-wave (**B**) and b-wave (**C**) of flash ERG at the indicated ages of the cohorts. Each dot represents one animal. Amplitudes were normalized to WT average at each age. N = 16-18 *Opa1$^{+/+}$; Sarm1$^{-/+}$*, 16-17 *Opa1$^{R290Q/+}$; Sarm1$^{-/+}$*, and 15-17 *Opa1$^{R290Q/+}$; Sarm1$^{-/-}$* mice per age group. One-way ANOVA followed by Tukey's multiple comparisons for each age group. Box plots denote minimum, first quartile, median, third quartile, and maximum values.

(**D**) Similar to Figure 6E, but at lower light intensities. Mean ± SD. Stimulus monitor at the bottom. The *Opa1$^{+/+}$; Sarm1$^{-/+}$* traces were superimposed onto the other two groups for comparisons. For the lowest two intensities, distinct peaks were poorly discriminated (average Z-score < 10) and were not included in analyses.

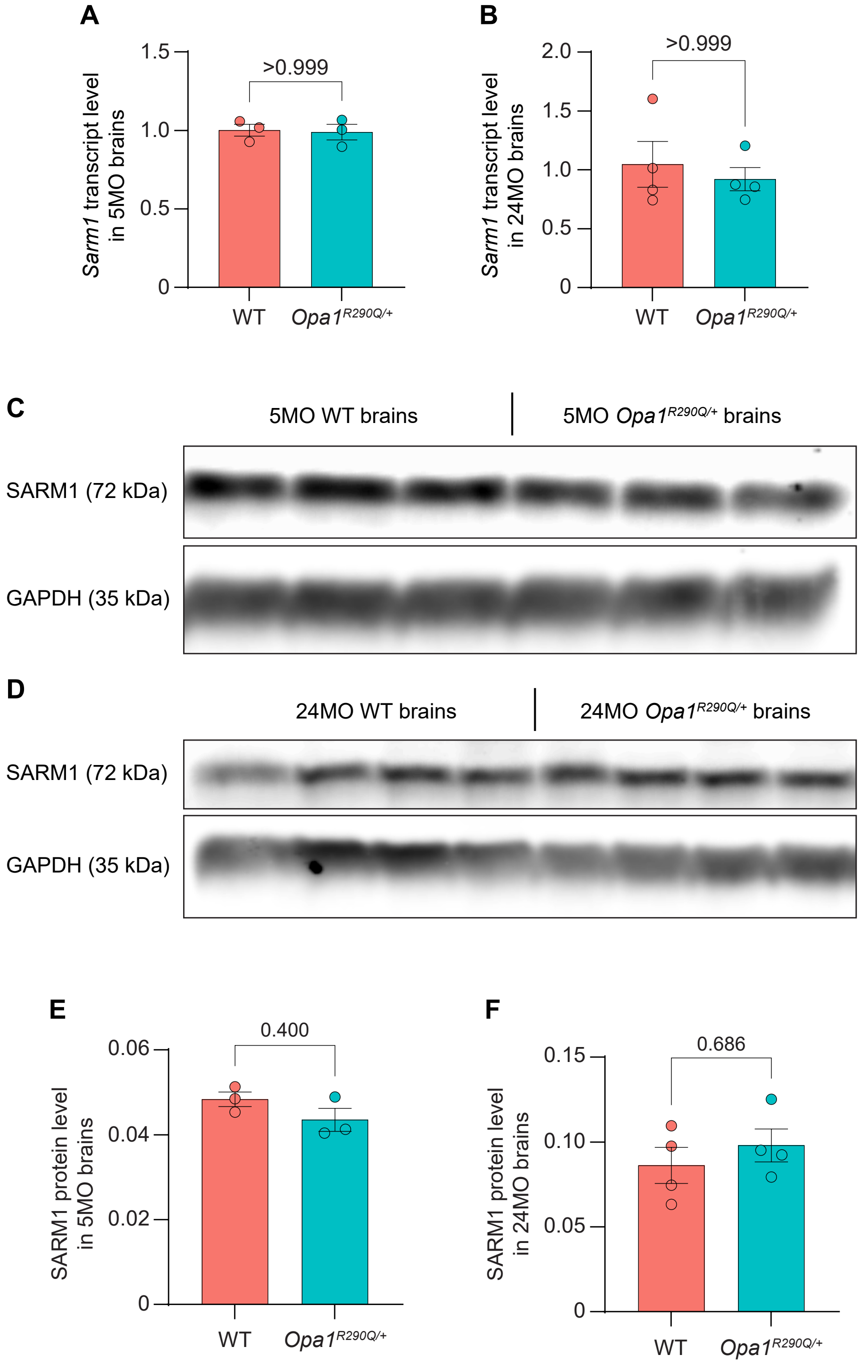

Supplemental Figure 9 - related to Figure 7. SARM1 expression levels are similar between WT and *Opa1*$^{R290Q/+}$ neurons
(**A** and **B**) *Sarm1* mRNA levels in brain samples from 5MO (**A**) and 24MO (**B**) mice were measured by RT-qPCR and quantified using the $2^{-\Delta\Delta CT}$ method. *Gapdh* was used as the control transcript. N = 3 WT and 3 *Opa1*$^{R290Q/+}$ (5MO) mice, and 4 WT and 4 *Opa1*$^{R290Q/+}$ (24MO) mice. Mean ± SEM. Mann-Whitney test.
(**C** and **D**) Western blot of SARM1 and GAPDH in whole brain lysates from 5MO (**C**) and 24MO (**D**) mice. Each lane is lysate from a different mouse of the indicated genotypes.
(**E** and **F**) SARM1 protein levels in (**C** and **D**) were quantified and normalized to GAPDH. N = 3 WT and 3 *Opa1*$^{R290Q/+}$ (5MO) mice, 4 WT and 4 *Opa1*$^{R290Q/+}$ (24MO) mice. Mean ± SEM. Mann-Whitney test.

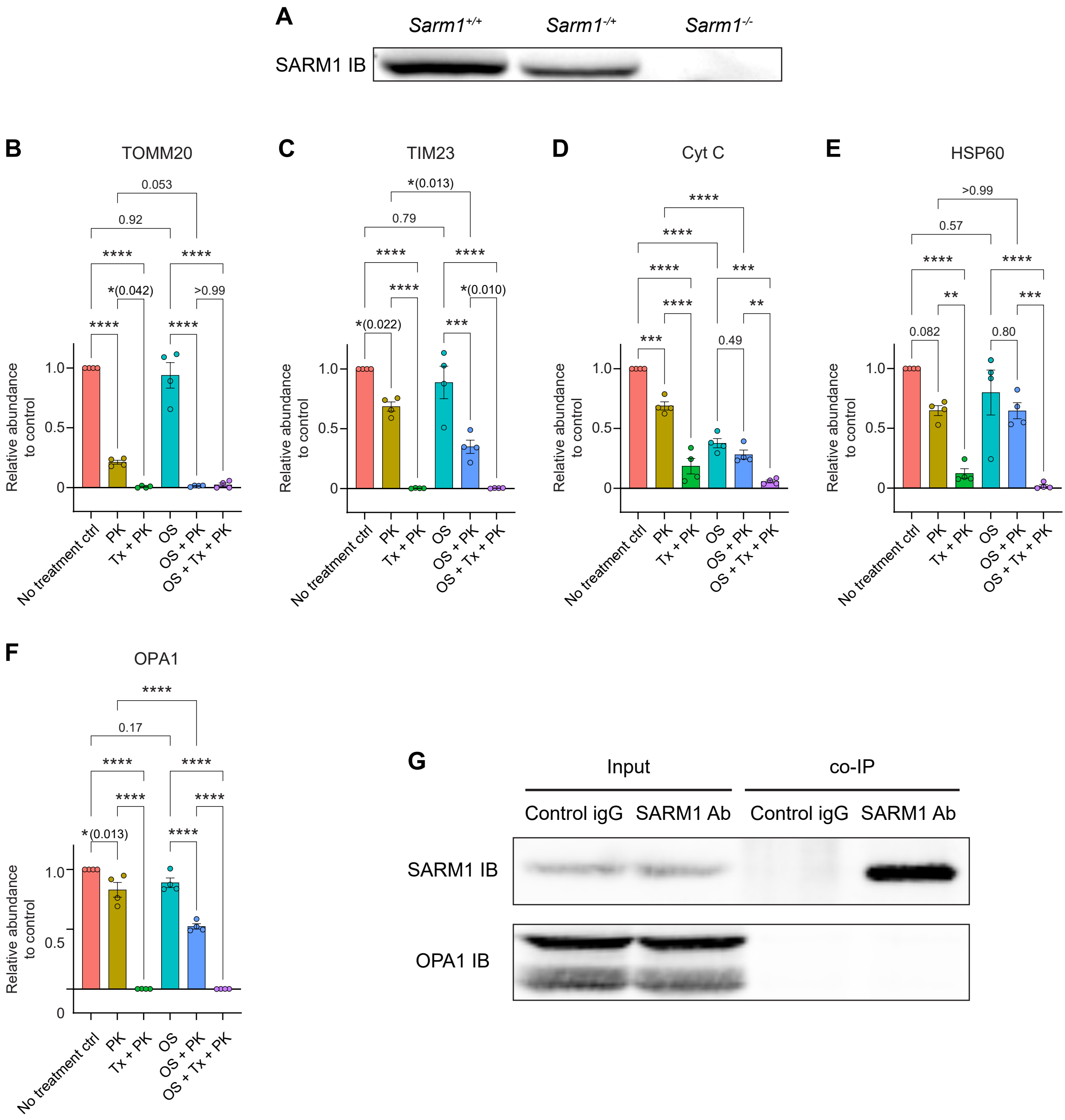

Supplemental Figure 10 - related to Figure 7. Western blot analysis of SARM1 and mitochondrial proteins
(**A**) Validation of the SARM1 antibody in *Sarm1*$^{+/+}$, *Sarm1*$^{-/+}$ and *Sarm1*$^{-/-}$ whole brain lysates.
(**B-F**) Quantification of TOMM20, TIM23, Cyt C, HSP60, and OPA1 in the Proteinase K protection assay from the experiments shown in Figures 7D. N = 4 mice from 4 experiments. Mean ± SEM. One-way ANOVA followed by Tukey's multiple comparisons. *P < 0.05; **P < 0.01; ***P < 0.001; ****P < 0.0001.
(**G**) Endogenous OPA1 was not co-precipitated when endogenous SARM1 was immunoprecipitated from mitochondria extracted from WT whole brains.

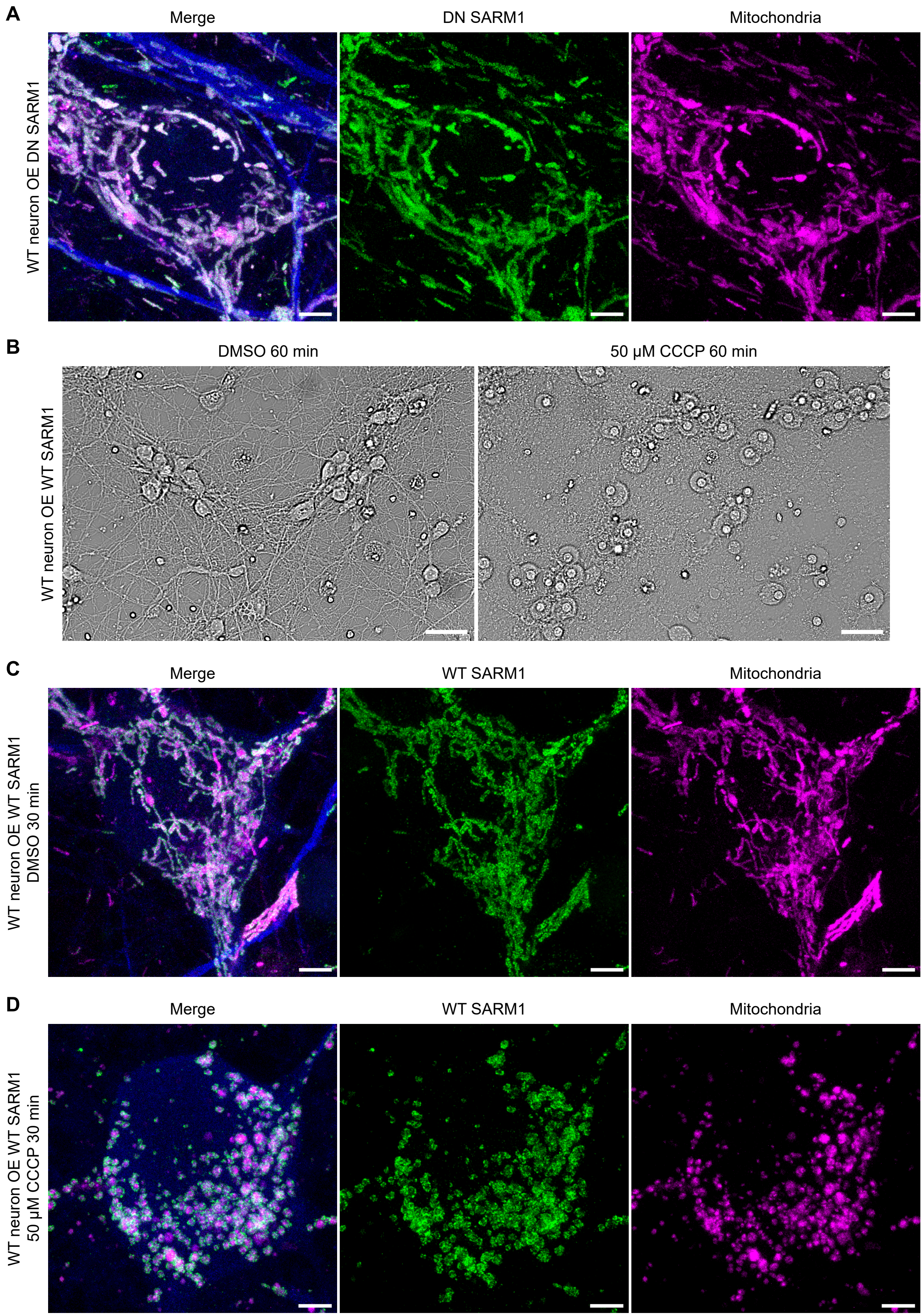

Supplemental Figure 11 - related to Figure 7. Both inactive and active SARM1 localize to mitochondria
(**A**) Overexpressed dominant negative SARM1 localized to mitochondria in DIV9 WT cortical neurons. MAP2 staining is shown in blue. Scale bars = 10 μm.
(**B**) Treatment with 50 μM CCCP for 60 minutes induced axon loss and soma rounding in DIV9 WT cortical neurons overexpressing WT SARM1. DMSO was used as the negative control. Scale bars = 50 μm.
(**C** and **D**) SARM1 still localized to mitochondria in DIV9 WT cortical neurons following 30 minutes of DMSO (**C**) or 50 μM CCCP (**D**) treatment. MAP2 staining is shown in blue. Scale bars = 10 μm.

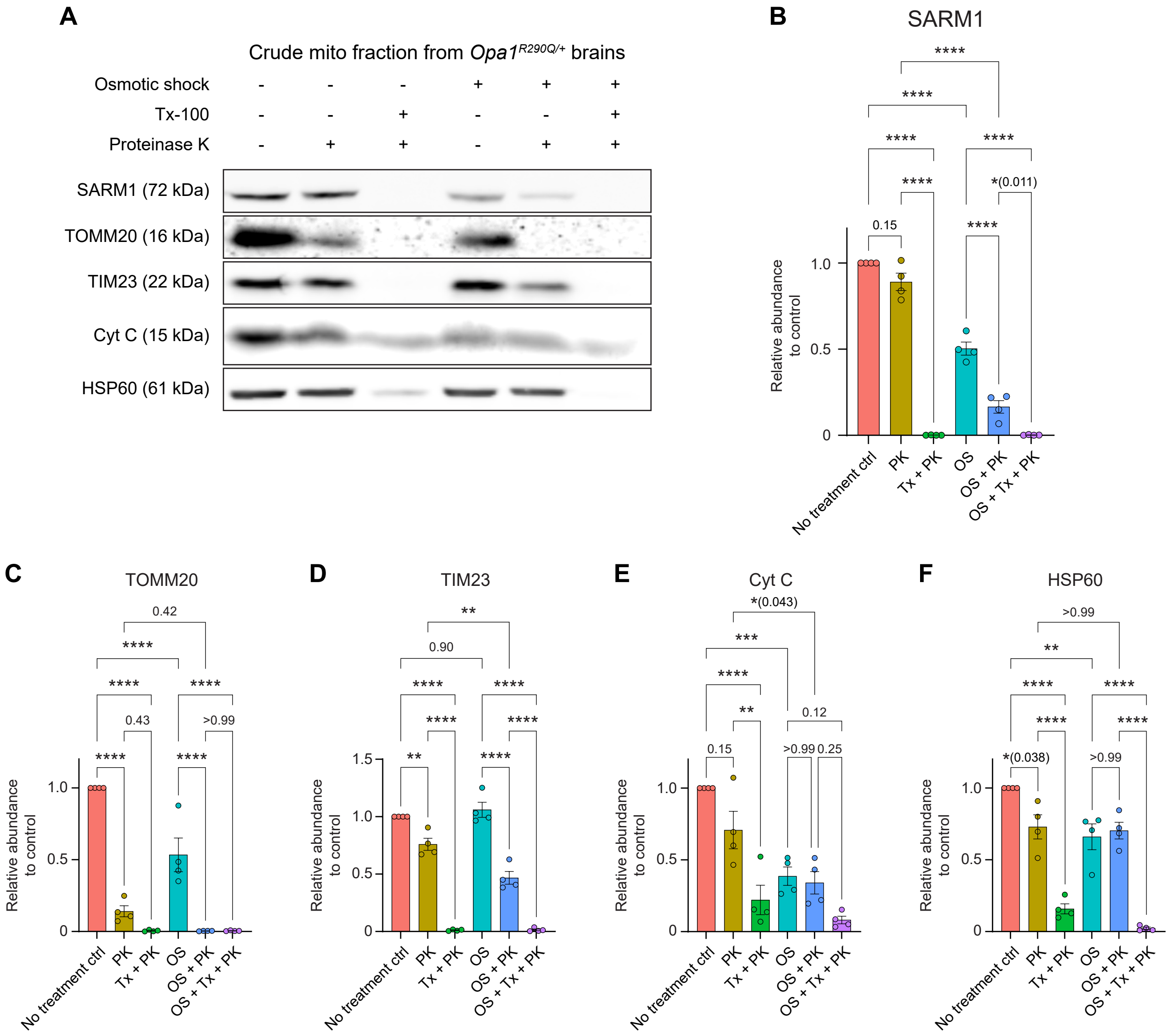

Supplemental Figure 12 - related to Figure 7. SARM1 is present in the mitochondrial IMS and IMM in the *Opa1*$^{R290Q/+}$ mutant
(**A**) Proteinase K protection assay on crude mitochondria fractions from *Opa1*$^{R290Q/+}$ whole brain samples.
(**B-F**) Quantification of SARM1, TOMM20, TIM23, Cyt C and HSP60 in the Proteinase K protection assay. N = 4 mice from 4 experiments. Mean ± SEM. One-way ANOVA followed by Tukey's multiple comparisons. *P < 0.05; **P < 0.01; ***P < 0.001; ****P < 0.0001.
