# Supplementary material for: SARM1 loss protects retinal ganglion cells in a mouse model of autosomal dominant optic atrophy
Source: J Clin Invest. 2025 May 9;135(12):e191315. doi: 10.1172/JCI191315 (PMC12165793; doi:10.1172/JCI191315)

Full unedited blot for Figure 7B

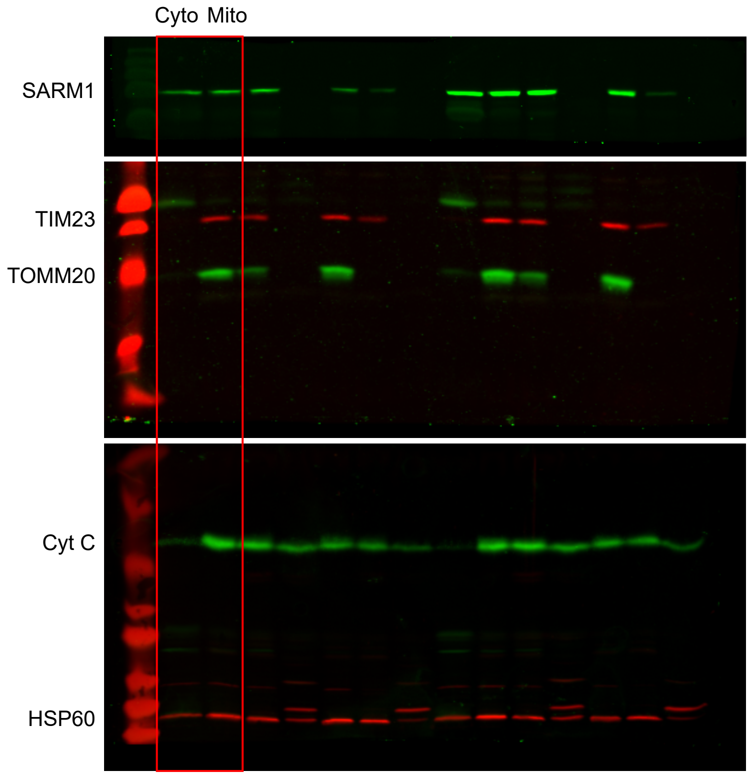

Full unedited blot for Figure 7D

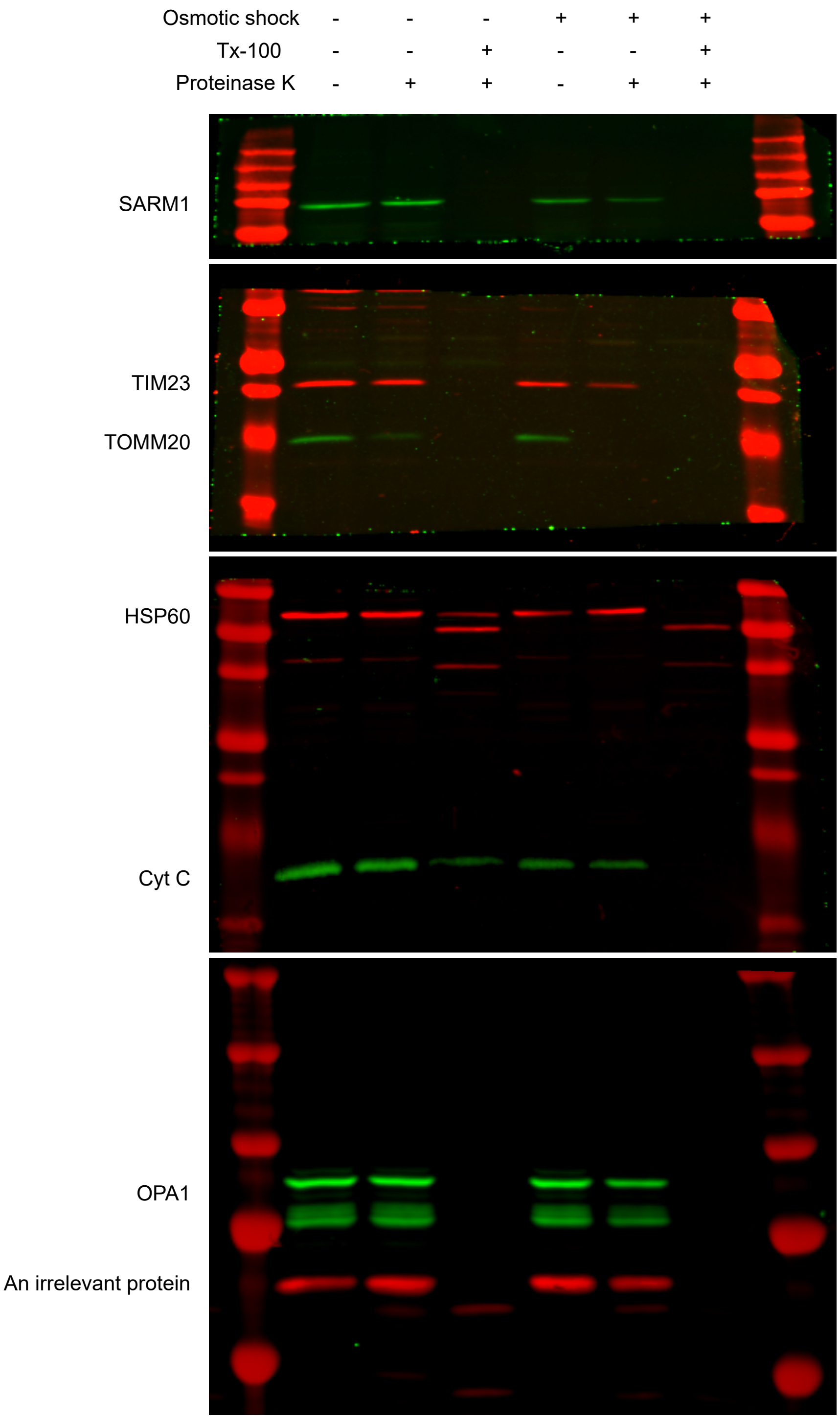

Full unedited blot for Supplemental Figure 1C

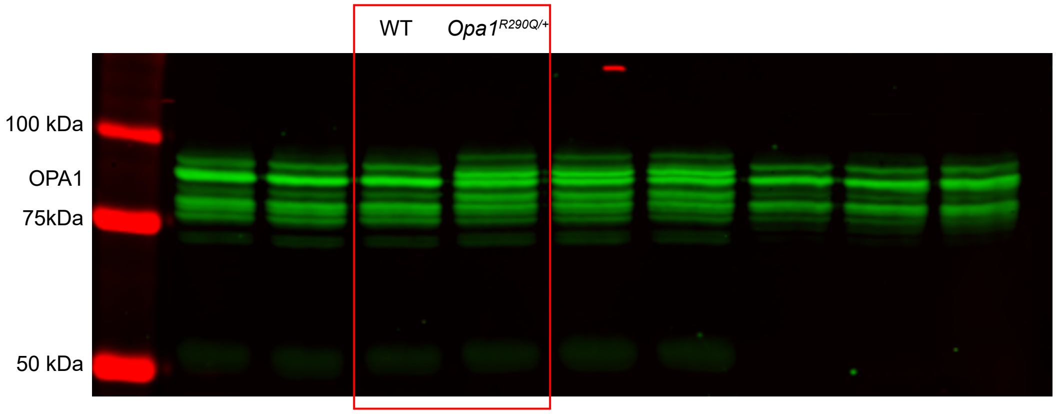

Full unedited gel for Supplemental Figure 1D

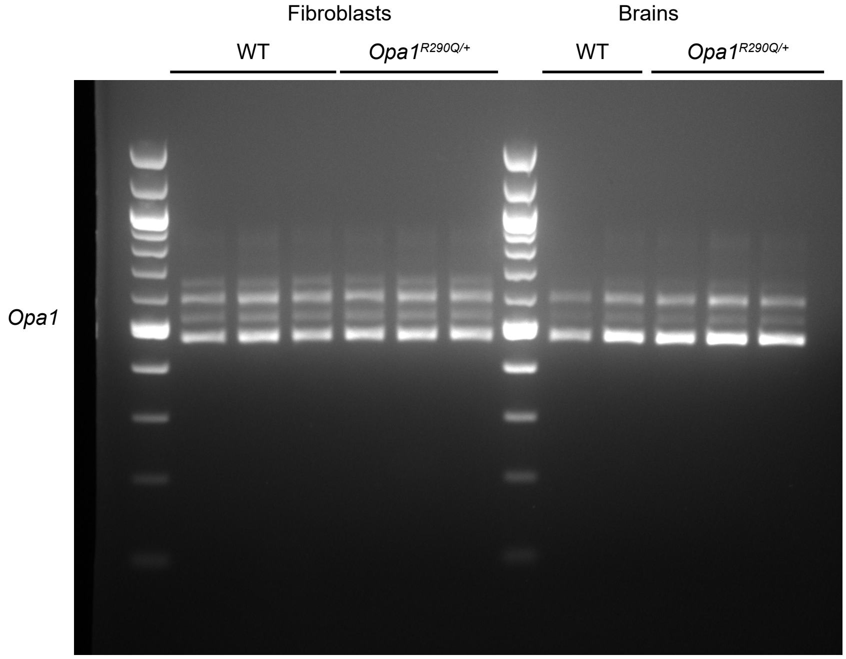

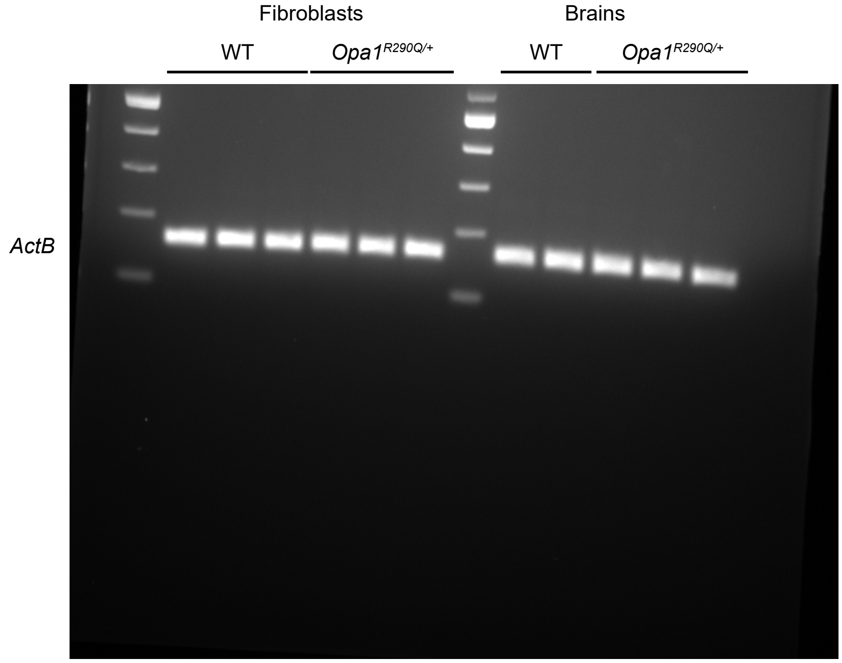

Full unedited blot for Supplemental Figure 9, C and D

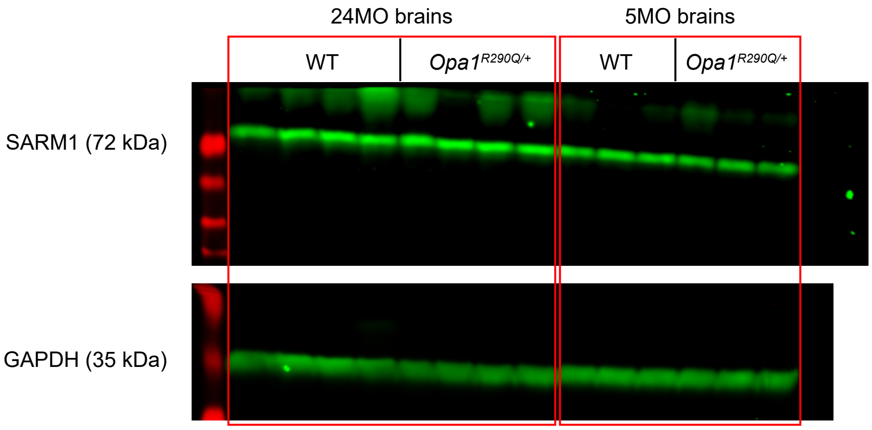

Full unedited blot for Supplemental Figure 10A

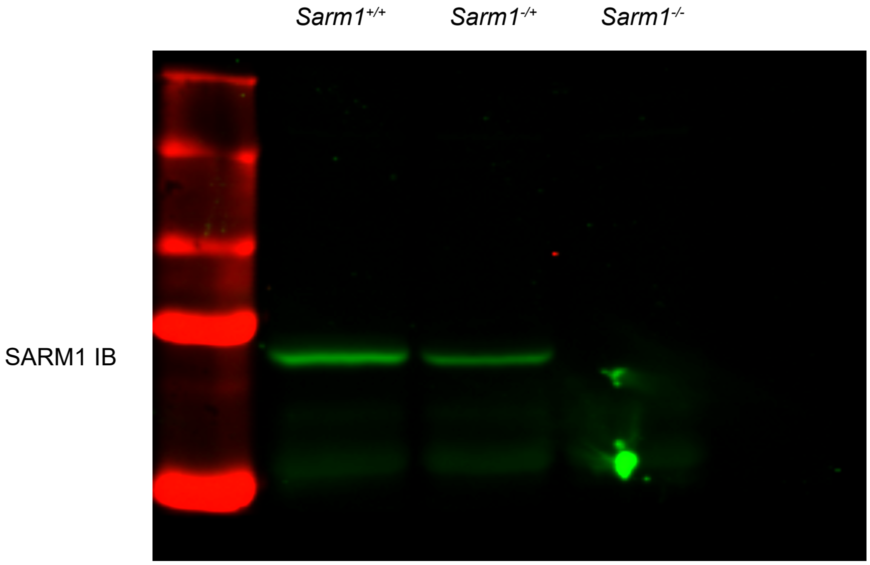

Full unedited blot for Supplemental Figure 10G

Input | co-IP

Control igG | SARM1 Ab | Control igG | SARM1 Ab

OPA1 IB (green)
Red is an irrelevant protein

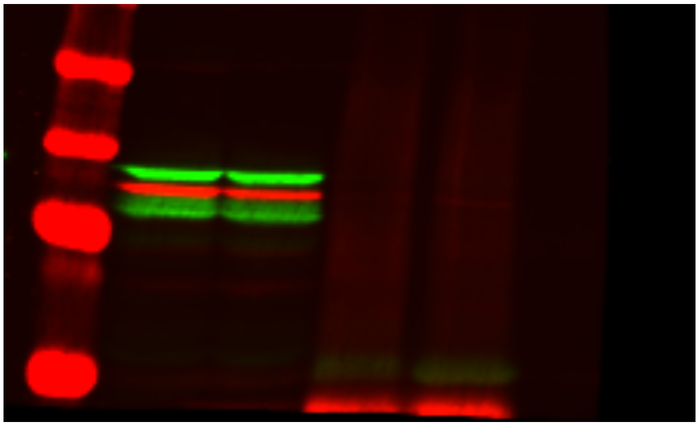

Input | co-IP

Control igG | SARM1 Ab | Control igG | SARM1 Ab

SARM1

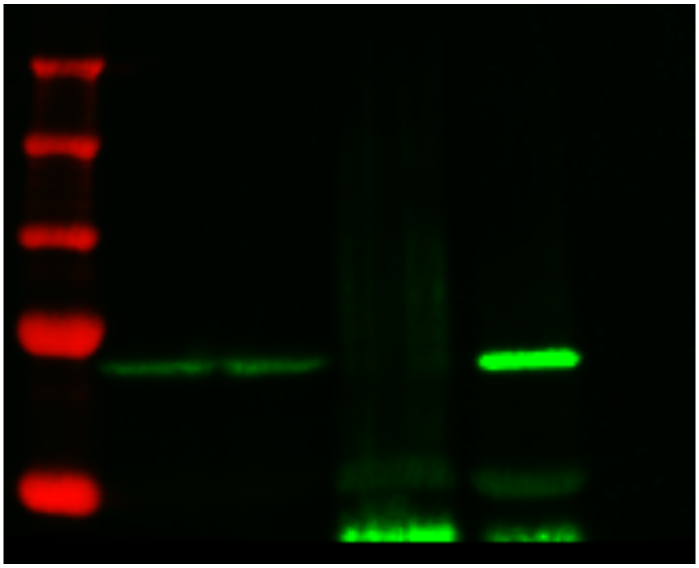

Full unedited blot for Supplemental Figure 12A

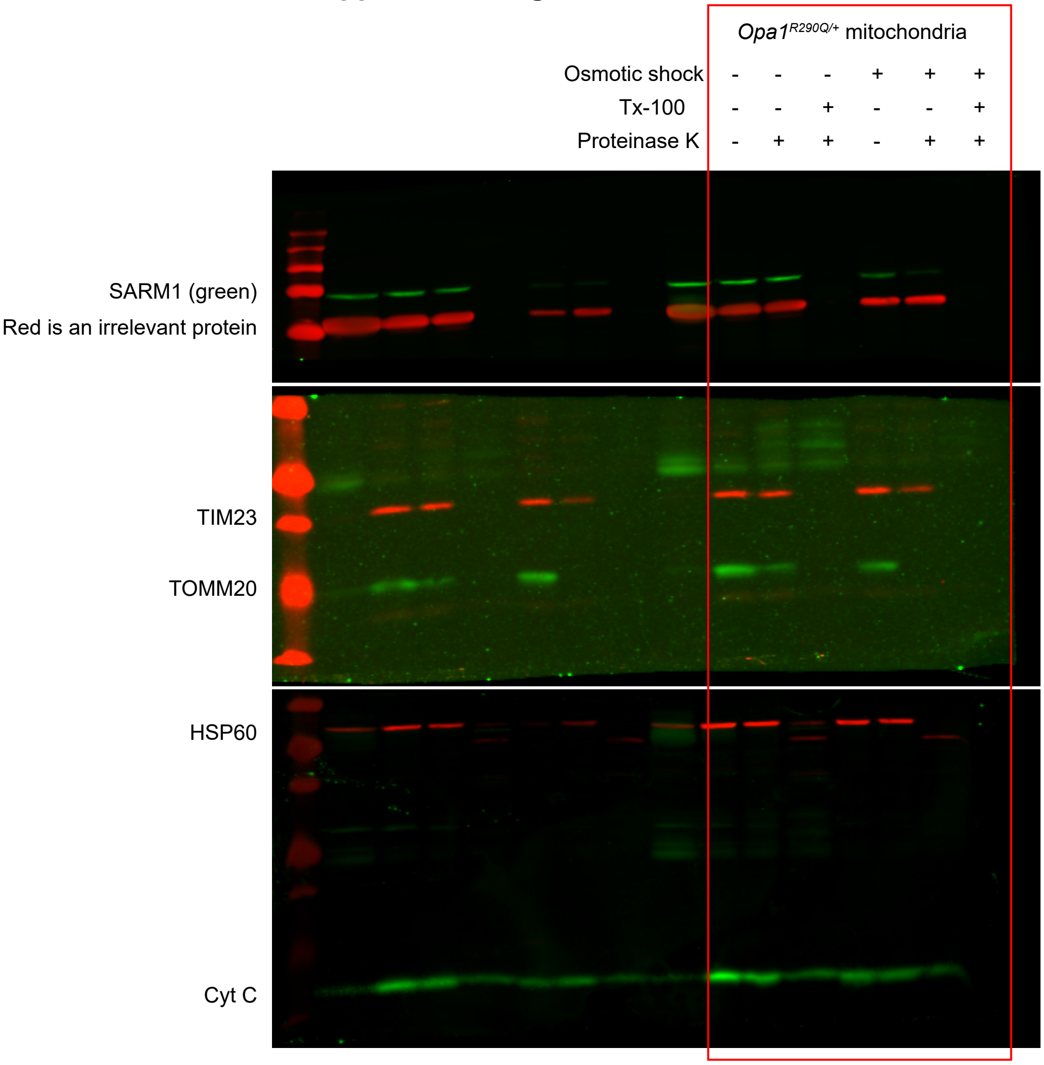

Supplement: Unedited blot and gel images [file jci-135-191315-s128.pdf]
